# Small RNA-Omics for Plant Virus Identification, Virome Reconstruction, and Antiviral Defense Characterization

**DOI:** 10.3389/fmicb.2018.02779

**Published:** 2018-11-20

**Authors:** Mikhail M. Pooggin

**Affiliations:** Institut National de la Recherche Agronomique, UMR BGPI, Montpellier, France

**Keywords:** small RNA, RNA interference, antiviral defense, siRNA, next generation sequencing, bioinformatics, virus/viroid diagnostics, virome reconstruction

## Abstract

RNA interference (RNAi)-based antiviral defense generates small interfering RNAs that represent the entire genome sequences of both RNA and DNA viruses as well as viroids and viral satellites. Therefore, deep sequencing and bioinformatics analysis of small RNA population (small RNA-ome) allows not only for universal virus detection and genome reconstruction but also for complete virome reconstruction in mixed infections. Viral infections (like other stress factors) can also perturb the RNAi and gene silencing pathways regulating endogenous gene expression and repressing transposons and host genome-integrated endogenous viral elements which can potentially be released from the genome and contribute to disease. This review describes the application of small RNA-omics for virus detection, virome reconstruction and antiviral defense characterization in cultivated and non-cultivated plants. Reviewing available evidence from a large and ever growing number of studies of naturally or experimentally infected hosts revealed that all families of land plant viruses, their satellites and viroids spawn characteristic small RNAs which can be assembled into contigs of sufficient length for virus, satellite or viroid identification and for exhaustive reconstruction of complex viromes. Moreover, the small RNA size, polarity and hotspot profiles reflect virome interactions with the plant RNAi machinery and allow to distinguish between silent endogenous viral elements and their replicating episomal counterparts. Models for the biogenesis and functions of small interfering RNAs derived from all types of RNA and DNA viruses, satellites and viroids as well as endogenous viral elements are presented and discussed.

## Introduction

Viral small RNAs have been discovered in *Nicotiana benthamiana* plants inoculated with potato virus X (genus *Potexvirus*, family *Alphaflexiviridae*) using RNA blot hybridization, and their abundance was found to gradually increase in the time course of viral infection (Hamilton and Baulcombe, 1999). A pioneering work of Kreuze et al. (2009) and the follow-up studies listed in Supplementary Tables S1, S2 and discussed below have established that both RNA and DNA viruses as well as viral satellites and viroids can be identified and their genomes partially or fully reconstructed by deep sequencing and bioinformatic analysis of small RNA population (small RNA-ome) from an infected plant. Likewise, small RNA deep sequencing can be used for virus detection and assembly of viral genomes from fungi (Vainio et al., 2015; Yaegashi et al., 2016; Donaire and Ayllón, 2017; Velasco et al., 2018) and from invertebrate animals (Wu et al., 2010; Aguiar et al., 2015; Fung et al., 2018), including insect vectors of the plant viruses transmitted in a propagative manner (Xu et al., 2012; Fletcher et al., 2016; de Haro et al., 2017). Such universality of the small RNA-omics approach for virus diagnostics is based on evolutionary conservation of the small RNA-generating RNA interference (RNAi) and gene silencing machinery that regulates gene expression and defends against invasive nucleic acids such as transposons, transgenes and viruses in most eukaryotes (Ghildiyal and Zamore, 2009; Nayak et al., 2013; tenOever, 2016). In the case of mammals where an interferon system has evolved to limit viral infections, potential contribution of RNAi to antiviral defenses remains a matter of debates (Jeffrey et al., 2017; tenOever, 2017). Nonetheless, virus-derived small RNAs generated by RNAi and/or other mechanisms are detectable by deep sequencing and could be used for virus identification in mammals and other vertebrates (Parameswaran et al., 2010; Wang F. et al., 2016).

This review article compiles and summarizes growing evidence demonstrating a universal power of small RNA-omics for diagnostics of all types of plant viruses and for exhaustive reconstruction of viromes of various cultivated and non-cultivated plants. Plant virus diagnostics and genome assembly by high-throughput sequencing of other target nucleic acids such as long single-stranded or double-stranded RNA and, in the case of DNA viruses, circular viral DNA enriched by a rolling circle amplification method have been previously reviewed (Boonham et al., 2014; Roossinck et al., 2015; Wu Q. et al., 2015; Jones et al., 2017; Roossinck, 2017; Maliogka et al., 2018; Jeske, 2018). Furthermore, this review illustrates mechanisms of biogenesis and function of small interfering RNAs (siRNAs) derived from plant RNA and DNA viruses, which have been dissected by deep sequencing and bioinformatics analysis of viral small RNA profiles in the model plant *Arabidopsis thaliana* (family *Brassicaceae*) and its RNAi-deficient mutant lines, and discusses conservation of these mechanisms in other plant species and families. Finally, the review presents models for biogenesis and possible functions of siRNAs derived from endogenous viral elements (EVEs) which represent host genome-integrated counterparts of extant or ancient episomal DNA viruses of the families *Caulimoviridae* and *Geminiviridae* and of some RNA viruses, and highlights characteristic differences in siRNA size, polarity and hotspot profiles between a silent EVE and its episomal copy that can potentially be released from the host genome to cause systemic and transmissible infection.

## All Families of Land Plant Viruses and Viroids Spawn Small Rnas in Infected Hosts

Reviewing a large number of studies that applied small RNA deep sequencing for virus identification and antiviral defense characterization in naturally or experimentally infected host plants (compiled in Supplementary Tables S1, S2) revealed that all 26 families of land plant viruses (Supplementary Table S1 and Supplementary List S1) and 2 families of viroids (Supplementary Table S2) spawn small RNAs that can be assembled into contigs representing partial or complete virus/viroid genomes. Such characteristic small RNAs, whose size-class and polarity profiles (analyzed in many but not all cases) are consistent with those of *bona fide* siRNAs generated by the plant RNAi machinery (described below), have been reported for circular single-stranded (ss)RNA viroids replicating in both chloroplasts (*Avsunviroidae*) and nuclei (*Pospiviroidae*) and for viral families with six types of genomes (Baltimore classification): (i) positive-sense ssRNA [(+)ssRNA: *Alphaflexiviridae, Benyviridae, Betaflexiviridae, Bromoviridae, Closteroviridae, Luteoviridae, Potyviridae, Secoviridae, Solemoviridae, Tombusviridae, Tymoviridae, Virgaviridae*], (ii) negative-sense ssRNA [(-)ssRNA: *Aspiviridae, Fimoviridae, Phenuiviridae, Rhabdoviridae, Tospoviridae*], (iii) double-stranded RNA [dsRNA: *Amalgaviridae, Endornaviridae, Partitiviridae, Reoviridae*], (iv) positive-sense ssRNA replicating via DNA intermediate by reverse transcription [ssRNA-RT: *Metaviridae, Pseudoviridae*], (v) single-stranded DNA [ssDNA: *Geminiviridae, Nanoviridae*] and (vi) double-stranded DNA replicating via RNA intermediate by reverse transcription [dsDNA-RT: *Caulimoviridae*] (Supplementary Tables S1, S2 and Supplementary List S1). Likewise, siRNAs have been reported for ssRNA satellites (unassigned family) associated with several helper (+)ssRNA viruses from *Bromoviridae, Secoviridae, Tombusviridae*, and *Virgaviridae* as well as for ssDNA betasatellites (*Tolecusatellitidae*) associated with helper ssDNA viruses of *Geminiviridae* (Supplementary Table S1 and Supplementary List S1). So far, ssDNA alphasatellites (*Alphasatellitidae*) associated with helper ssDNA viruses of *Geminiviridae* and *Nanoviridae* (see Supplementary List S1) have not been analyzed by small RNA sequencing. It is conceivable that alphasatellites are also targeted by the plant RNAi machinery like their helper viruses. Same assumption applies to those genera (and species) within the above-listed plant virus/satellite/viroid families for which small RNA deep sequencing data are not available yet (Supplementary List S1, genera highlighted in red).

The host plant species that accumulated viral small RNAs sufficient for virus, satellite or viroid identification belong to 53 botanical families of the kingdom Plantae, with 49 families from the clade Angiosperms (Supplementary List S2 and Supplementary Tables S1, S2). Within angiosperms the families of eudicots (*n* = 37), monocots (*n* = 10) and ANITA grade basal angiosperms (*n* = 2) are represented with at least one plant species. The basal angiosperms are represented with *Amborella trichopoda* (family *Amborellaceae*) that generated small RNAs from three endogenous caulimovirids of a tentative genus *Florendovirus* (*Caulimoviridae*) and water lily (*Nymphaeaceae*) that accumulated small RNAs from (i) cucumber mosaic virus (*Cucumovirus, Bromoviridae*), (ii) an uncharacterized virus from the genus *Cytorhabdovirus* (*Rhabdoviridae*) and (iii) an uncharacterized, likely endogenous, caulimovirid (Kreuze, 2014). Outside angiosperms the representatives of only few plant families have been characterized by the presence by viral small RNAs. These include queen sago palm (*Cycadaceae*), moss *Physcomitrella patens* (*Funariaceae*) and green algae *Chara coralline* (*Characeae*), which all accumulated small RNAs from putative endogenous caulimovirids (Kreuze, 2014), and European water clover (*Marsileaceae*) that accumulated small RNAs from turnip yellows virus (*Polerovirus, Luteoviridae*) (Kreuze, 2014).

Only a few viruses that can infect land plant species outside the clade Angiosperms have been identified so far, including a few viruses and virus-like agents identified in gymnosperms and ferns (Hull, 2014). Recently, EVEs of the family *Caulimoviridae* have been identified in the genomes of gymnosperms, ferns and club mosses (Diop et al., 2018; Gong and Han, 2018). Taking into consideration EVEs of the genus *Florendovirus* (*Caulimoviridae*) identified in the genomes of angiosperms (Geering et al., 2014), almost every vascular land plant (Tracheophyta) contains endogenous caulimovirids, some of which can potentially give rise to episomal viruses as reported for EVEs from the genera *Badnavirus, Solendovirus*, and *Petuvirus* (Ndowora et al., 1999; Lockhart et al., 2000; Richert-Pöggeler et al., 2003; discussed below). Since all land plants and green algae possess the small RNA-generating RNAi machinery (You et al., 2017), it is conceivable that gymnosperms, ferns and club mosses can potentially accumulate siRNAs derived from endogenous caulimovirids and/or their extant not-yet-identified episomal counterparts. Likewise, genomes of all land plants contain long terminal repeat (LTR) retrotransposons of the families *Metaviridae* (Ty3/Gypsy) and *Pseudoviridae* (Ty1/Copia) which can give rise to siRNAs as has been reported for the angiosperms *A. thaliana* (Creasey et al., 2014; Masuta et al., 2017), strawberry (Šurbanovski et al., 2016), mangrove (Wang Y. et al., 2018), maize (Alejandri-Ramírez et al., 2018) and wheat (Sun et al., 2013); transposon-derived small RNAs were also reported for gymnosperms such as *Picea glauca* (Liu and El-Kassaby, 2017) and *Cryptomeria japonica* (Ujino-Ihara et al., 2018).

Eukaryotic algae host large dsDNA viruses of the family *Phycodnaviridae* and a few species of RNA viruses (Hull, 2014). The presence of the small RNA-generating RNAi machinery in green and red algae (You et al., 2017; Lee et al., 2018) argues for its possible role in antiviral defenses. A recent study of the brown alga *Fucus serratus* has revealed predominantly 21-nucleotide (nt) small RNAs with 5′U derived from both strands of a (-)ssRNA bunya/phlebo-like virus (unassigned *Bunyavirales*) and from both strands of an LTR Copia retrotransposon (*Pseudoviridae*), which is indicative of a functional antiviral RNAi response (Waldron et al., 2018).

## Toward More Exhaustive Reconstruction of Complex Viromes by Deep Small Rna Sequencing and Bioinformatics

In a few reported cases, virus-derived small RNAs were below detection by small RNA sequencing, although the corresponding virus could be identified by other methods. For instance, in a co-infected rice plant, PCR-positive for rice tungro spherical virus (RTSV, *Waikavirus, Secoviridae*) and rice tungro bacilliform virus (RTBV, *Tungrovirus, Caulimoviridae*), only RTBV-derived siRNAs could be readily identified by deep sequencing, while RTSV-specific reads were negligible and comparable to those in a control “virus-free” plant (Zarreen et al., 2018). Since another waikavirus and viral species from other genera of *Secoviridae* are readily identified by small RNA sequencing (see Supplementary Table S1 and references therein) and because rice plants can generate siRNAs from many types of RNA and DNA viruses (Yan et al., 2010; Jiang et al., 2012; Xu et al., 2012; Kreuze, 2014; Rajeswaran et al., 2014a; Hong et al., 2015; Wu J. et al., 2015; Xu and Zhou, 2017; Yang et al., 2017; Jimenez et al., 2018; Lan et al., 2018; see Supplementary Table S1 and Supplementary List S1), the failure to identify RTSV-specific siRNAs can be explained by low titer of the virus. In this and other similar cases, deeper sequencing of small RNA-ome could have helped to uncover viral siRNAs. Indeed, a recent systematic study has established that, under low sequencing depths, most bioinformatic pipelines that use *de novo* assembly of small RNAs fail to identify low-titer persistent viruses in apple and grapevine, while those viruses can be readily identified at higher sequencing depths (Massart et al., 2018). In another study that compared small RNA-seq with long RNA-seq, a member of the genus *Cytorhabdovirus* (*Rhabdoviridae*) could be identified by *de novo* assembly only in the long RNA dataset, because of low abundance of the viral reads in the small RNA dataset (Pecman et al., 2017). Notably, in the same study, 14 distinct viral/viroid species representing various viral families could be readily identified by sequencing small RNAs from eight different hosts (Pecman et al., 2017; see Supplementary Tables S1, S2). Furthermore, viral species representing all four genera of *Rhabdoviridae* including *Cytorhabdovirus* have been identified by small RNA sequencing from 10 different plant species (Roy et al., 2013b,c; Kreuze, 2014; Hartung et al., 2015; Yan et al., 2015; Verdin et al., 2017; Xu et al., 2017; Yang et al., 2017; Mwaipopo et al., 2018) (see Supplementary Table S1).

It has been speculated that occasional failure to detect viral small RNAs is due to virus-encoded silencing suppressor proteins that block viral siRNA biogenesis. However, available evidence indicates the contrary. For example, wild-type RNA viruses of the families *Bromoviridae* (cucumber mosaic virus), *Potyviridae* (turnip mosaic virus) and *Virgaviridae* (oilseed rape mosaic virus) spawn highly abundant siRNAs constituting 30–70% of total (viral + host) small RNA sequencing reads, whereas their suppressor protein-deficient mutant derivatives spawn siRNAs of much lower abundance that correlates with strongly reduced titers of viral genomic RNA (Garcia-Ruiz et al., 2010; Wang et al., 2010, 2011; Malpica-López et al., 2018). The most extreme example of an RNA virus spawning highly abundant siRNAs is pelargonium line pattern virus (*Pelarspovirus, Tombusviridae*) whose siRNAs constituted ca. 90% of total small RNA reads in symptomless *N. benthamiana* (Pérez-Cañamás et al., 2017). Among DNA viruses, cauliflower mosaic virus (*Caulimovirus, Caulimoviridae*)-derived siRNAs constitute ca. 50% of total small RNA reads in *A. thaliana* at late stages of infection (Blevins et al., 2011), which could allow for *de novo* assembly of viral siRNA reads into a single terminally redundant contig representing the entire circular viral genome of 8 kb (Seguin et al., 2014).

The universal power of small RNA sequencing for identification and reconstruction of known and unknown RNA and DNA viruses in single and mixed infections can be further exemplified by several comprehensive studies. Xu et al. (2017) have reconstructed the virome of tomatoes in China by sequencing small RNAs from 170 samples and identified 22 viruses representing 12 genera of RNA and DNA viruses and 2 viroids (see “Xu et al., 2017” in Supplementary Tables S1, S2): the complete genomes were reconstructed from small RNAs by *de novo* and reference-based assembly for 13 of the 22 viruses and near complete ones (>90% genome sequence) for another 5 viruses. Likewise, small RNA-omics has been successfully used for reconstruction of the complex viromes of vegetatively cultivated sweet potatoes (Kreuze et al., 2009; Cuellar et al., 2011; Kashif et al., 2012; De Souza et al., 2013; Mbanzibwa et al., 2014; Cuellar et al., 2015; Untiveros et al., 2016; Cao et al., 2017; see Supplementary Tables S1 and Supplementary List S1) and grapevines (Pantaleo et al., 2010; Zhang et al., 2011, 2014; Alabi et al., 2012; Giampetruzzi et al., 2012; Wu et al., 2012; Glasa et al., 2014, 2015; Seguin et al., 2014; Maliogka et al., 2015; Chiumenti et al., 2016a; Eichmeier et al., 2016; Barrero et al., 2017; Czotter et al., 2018; see Supplementary Tables S1, S2 and Supplementary List S1), which led to identification of novel RNA and DNA viruses and viroids among other virome components. Mwaipopo et al. (2018) have identified 15 viral species from 10 genera of RNA and DNA viruses in the virome of common bean (see Supplementary Table S1 and Supplementary List S1). Verdin et al. (2017) have used small RNA-omics to survey 46 species of ornamental plants and identify multiple known and unknown viruses representing 22 genera of RNA and DNA viruses and 2 viroids (see Supplementary Tables S1, S2), albeit some of the novel viruses were represented only with short contigs.

In effort to improve identification and reconstruction of different types of viral genomes from various hosts, Barrero et al. (2017) have developed bioinformatics algorithms for small RNA assembly using selected 21-nt and 22-nt versus 24-nt reads which represent most abundant size-classes of viral siRNAs (discussed below). This work followed earlier studies where a host genome filtering step had been employed to allow for assembling viral small RNAs in longer contigs (Li et al., 2012; Seguin et al., 2014) that, in some cases, represented the complete genomes of both RNA and DNA viruses and viroids (Seguin et al., 2014). It should be noted that the host genome filtering step removes from a small RNA-ome not only the host-derived endogenous small RNAs but also, in some cases, viral small RNA reads occasionally matching the host genome, which may lead to incomplete assembly of a viral genome (Seguin et al., 2014). This would also be the case for those viruses that have EVE counterparts integrated in the host genome. Nonetheless, for representatives of both DNA (*Caulimoviridae* and *Geminiviridae*) and RNA (*Virgaviridae*) viruses the host genome filtering allowed for *de novo* assembly of complete viral genomes, albeit this step was implemented after small RNA assembly into contigs by a short read assembler, followed by assembly of all the unmapped contigs by a long read assembler (Seguin et al., 2014). A recently developed tool VirusDetect (Zheng et al., 2017a) implements for small RNA-seq datasets both filtering through a host genome and mapping onto a database of viral reference genomes to enrich for viral reads before assembly. The latter approach may not be applicable for identification of novel viruses with low homology to any known virus in the reference database. Other issues concerning reliability, reproducibility and sensitivity of virus diagnostics by small RNA sequencing and bioinformatics analysis have been recently investigated and discussed by Massart et al. (2018).

Importantly, the sensitivity of small RNA-seq for detection of potyviruses (*Potyviridae*) in *N. tabacum* is reportedly 10 times higher than that of quantitative RT-PCR (Santala and Valkonen, 2018). Likewise, sweet potato feathery mottle virus (*Potyvirus, Potyviridae*) with extremely low titer (below immune detection) and symptomless in sweet potato could be assembled by sequencing viral small RNAs that covered the complete reference genome at an average 470 reads per nucleotide (Kreuze et al., 2009). Notably, *Potyviridae* is among those viral families for which many species from various hosts have been identified by small RNA-seq (see Supplementary Table S1 and references therein).

Concerning identification of novel viruses, analysis of small RNA contigs using BlastN and BlastX is recommended (Massart et al., 2018). In this case, any bioinformatics tricks making viral contigs longer, such as those described above and additional ones in Massart et al. (2018), should help to identify a virus or virus-like agent with very low homology to known viral sequences available in the NCBI GenBank or other databases.

A notable limitation of the small RNA-omics approach is its inability to fully and reliably reconstruct from mixed infections genome sequences of two (or more) strains or genetic variants of the same virus when they share high sequence identity. Thus, two strains of *Potato virus Y* (*Potyvirus, Potyviridae*) coinfecting a potato plant could be *de novo* assembled into strain-specific small RNA contigs only for a 1 kb 5′-portion of the virus genomes which share 75% nucleotide identity. The remaining portions of the genomes sharing >87% identity merged into one chimeric small RNA contig and could be separated only by a reference-based approach (Turco et al., 2018). Likewise, two strains of *Pepino mosaic virus* (*Potexvirus, Alphaflexiviridae*) with 82–87% identity in co-infected tomatoes (Li et al., 2012; Turco et al., 2018) and two strains of *Potato virus X* (*Potexvirus*) with 80% identity in a co-infected potato (Kutnjak et al., 2014) could be distinguished by small RNA sequencing, but their (near-)complete genome sequences were reconstructed only by using reference-based approaches. In these and similar cases, however, recombinant viral genomes potentially present in the mixed virome quasispecies population cannot be reliably reconstructed from small RNA reads. Such reconstruction can possibly be achieved by single long molecule sequencing (e.g., using PacBio or Nanopore technology) in combination with small RNA-seq. It is worth noting that viral small RNAs faithfully represent a consensus master genome sequence and minor variants in the quasispecies populations of both RNA and DNA viruses (Seguin et al., 2014, 2016; Kutnjak et al., 2015). Thus, small RNA sequencing-based identification and subsequent correction of three single nucleotide cloning errors in the genome of an RNA tobamovirus (*Virgaviridae*) enabled construction of a fully biologically active infectious clone of the virus (Seguin et al., 2014; Malpica-López et al., 2018).

Since viral siRNAs are relatively more stable than long RNA and DNA molecules, small RNA deep sequencing can be applied in paleovirology. Thus, Smith et al. (2014) have reported identification and reconstruction of an ancient isolate of *Barley stripe mosaic virus* (*Hordeivirus, Virgaviridae*) by sequencing small RNAs extracted from a 700 years-old seed of barley, with 99.4% of the contemporary virus reference genome being covered by small RNA contigs. Likewise, Hartung et al. (2015) have used small RNA-seq and reference-based assembly to reconstruct citrus leprosis virus cytoplasmic type 2 (*Cilevirus*, unassigned family) and citrus leprosis virus nuclear type (*Dichorhavirus, Rhabdoviridae*) from herbarium specimens of orange fruit peals collected in 1967 and 1948, respectively. The genome sequences of these isolates were found to be respectively 99% and 80% identical to those of contemporary isolates of the two viruses that had previously been identified by sequencing and *de novo* assembly of small RNAs from fresh citrus samples (Roy et al., 2013a,b,c) (Supplementary Table S1).

## Components of the Plant Small Rna-Generating Rnai and Gene Silencing Machinery

Components and functionality of the plant small RNA-generating RNAi machinery in endogenous gene silencing pathways have been dissected using combined genetic, small RNA-seq, long RNA-seq and biochemical approaches for the model eudicot *A. thaliana* using a comprehensive collection of its RNAi-deficient mutant lines. Similar but less comprehensive studies have also been performed for other angiosperms such as the eudicot *N. benthamiana* and the monocot *Oryza sativa*, and also for non-angiosperms such as the moss *P. patens*. Based on these studies as well as small RNA-omics, transcriptomics and phylogenomics analyses of other plant species, the endogenous small regulatory RNAs have been divided into miRNAs and siRNAs which are generated from stem-and-loop ssRNA and dsRNA precursors, respectively, by Dicer-like (DCL) family proteins. Both miRNAs and siRNAs are then loaded onto Argonaute (AGO) family proteins to guide the resulting RNA-induced silencing complexes (RISCs) to complementary target RNA and/or DNA molecules (reviewed in Rogers and Chen, 2013; Borges and Martienssen, 2015; Fang and Qi, 2016). Plant endogenous siRNAs are further subdivided in several classes including inverted repeat hairpin-derived siRNAs (hpsiRNAs), natural antisense transcript siRNAs (natsiRNAs), double-strand-break-induced siRNAs (diRNAs), secondary siRNAs and heterochromatic siRNAs (hcsiRNAs) (Borges and Martienssen, 2015). Secondary siRNAs include phased siRNAs (phasiRNAs), *trans*-acting siRNAs (tasiRNAs) and epigenetically activated siRNAs (easiRNAs) and their biogenesis requires miRNA-directed cleavage of target mRNA or other Pol II transcripts and then involves complementary RNA synthesis by RNA-dependent RNA polymerase (RDR) family protein(s) (RDR6 or RDR1), followed by DCL4- and/or DCL2-mediated processing of the resulting dsRNA into 21-nt and 22-nt siRNAs. Secondary siRNAs are loaded onto AGO1 or AGO2 clade proteins to post-transcriptionally repress their target RNAs through *in cis* or *in trans* cleavage and degradation (Borges and Martienssen, 2015). HcsiRNAs are produced from multiple heterochromatic loci with cytosine-methylated DNA at CG, CHG, and CHH sites and modified histones, which include inactive transposons and repetitive DNA elements, by a mechanism that involves plant-specific DNA-dependent RNA polymerase IV (Pol IV) transcribing methylated DNA, RDR2 converting the Pol IV transcripts into dsRNA and DCL3 processing the resulting dsRNA into 24-nt siRNAs (Blevins et al., 2015). HcsiRNAs are loaded onto AGO4/6/9 clade proteins to maintain heterochromatic state through siRNA-directed DNA methylation (RdDM) of cognate DNA loci mediated by *de novo* methyltransferase DRM2. Maintenance of RdDM also requires another plant-specific DNA-dependent polymerase, Pol V (Liu et al., 2018), while initiation of RdDM likely involves Pol II (reviewed in Borges and Martienssen, 2015; Matzke et al., 2015).

**FIGURE 1:**
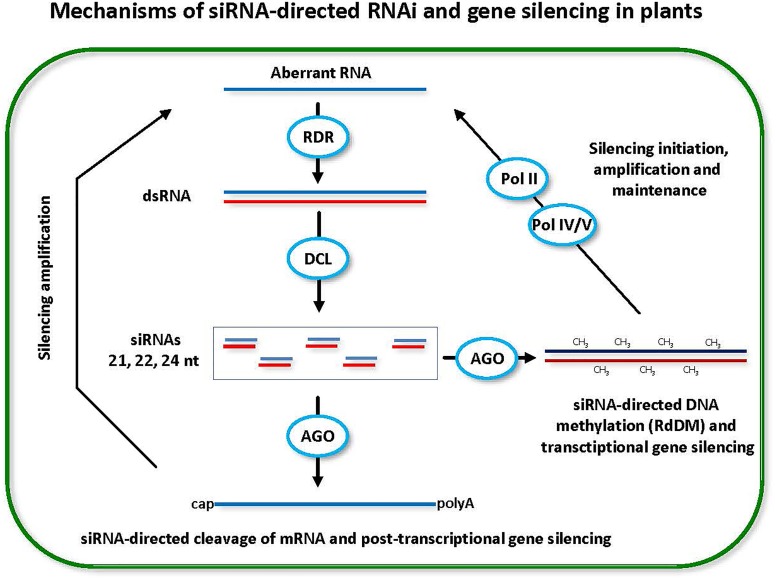
Small interfering RNA (siRNA)-directed RNA interference (RNAi) and gene silencing in plants. Double-stranded RNA (dsRNA) is a trigger of RNAi and gene silencing at both post-transcriptional and transcriptional levels (PTGS and TGS). Dicer-like (DCL) family enzymes catalyze processing of dsRNA into siRNA duplexes. One of the duplex strands gets associated with an Argonaute (AGO) family protein and the resulting RNA-induced silencing complex targets cognate genes for PTGS through target mRNA cleavage and/or TGS through target DNA methylation (CH_3_). Positive feedback loops reinforce both PTGS and TGS. In PTGS, the mRNA cleavage products (aberrant RNAs) are converted by RNA-dependent RNA polymerase (RDR) activity into dsRNA precursors of siRNAs. In TGS, the methylated DNA and the target (to-be-methylated) DNA are transcribed by Pol IV and Pol V, respectively. The Pol IV transcripts are converted by RDR activity into dsRNA precursors of siRNAs, while the Pol V transcripts serve as scaffolds that interact with siRNA-AGO complexes and recruit DNA methyltransferase that mediates *de novo* RNA-directed DNA methylation (RdRM). Pol II is likely involved in initiation of RdDM and TGS by producing aberrant transcripts that are recognized by RDR and converted into dsRNA precursors of siRNAs.

Plant miRNAs of 21 and 22 nts in length, produced from the *MIR* genes Pol II transcripts by DCL1 and loaded onto AGO1, act post-transcriptionally through cleavage or translational repression of endogenous target mRNAs (reviewed in Rogers and Chen, 2013) and do not contribute directly to the repression of RNA or DNA viruses mediated by siRNA-generating RNAi machinery (Figure 1; described below). However, several miRNAs are involved in antiviral defense via regulation of plant defense genes and components of the RNAi machinery (reviewed in Carbonell and Carrington, 2015; Pooggin, 2016). Moreover, some miRNAs are involved in recognition of transcriptionally active LTR (and other) transposons and initiation of RDR6- and DCL4-dependent biogenesis of abundant 21-nt and 22-nt easiRNAs (Creasey et al., 2014). Production and activity of easiRNAs likely leads to a transition from post-transcriptional to transcriptional silencing when Pol II transcription would be replaced by Pol IV/V transcription, switching from 21-nt or 22-nt to 24-nt siRNA production and epigenetic silencing by RdDM (Borges and Martienssen, 2015). Similar mechanisms might be involved in recognition and repression of EVEs upon their initial integration into the host genome or after stress-dependent activation of silent EVEs (discussed below).

All classes of plant endogenous (and viral) small RNAs are methylated at 2′OH of 3′-terminal nucleotide by the methyltransferase HEN1, which protects small RNA from degradation. Notably, suppressor proteins of some (+)ssRNA viruses (*Tobamovirus, Potyvirus, Tombusvirus*) can interfere with HEN1-mediated methylation of miRNAs and viral and endogenous siRNAs, likely through binding and sequestering small RNA duplexes produced by DCLs (Akbergenov et al., 2006; Blevins et al., 2006; Lózsa et al., 2008; Malpica-López et al., 2018). Such suppressor activity, however, does not lead to decreased accumulation of viral siRNAs (Malpica-López et al., 2018). Representatives of dsDNA-RT (Cauliflower mosaic virus, *Caulimovirus, Caulimoviridae*) and ssDNA (Cabbage leaf curl virus, *Begomovirus, Geminiviridae*) viruses do not interfere with HEN1 activities in *A. thaliana* (Blevins et al., 2006).

Loading of endogenous (and viral) small RNAs onto specific AGOs is determined largely by the small RNA 5′-terminal nucleotide and length, but also other factors (reviewed in Borges and Martienssen, 2015; Carbonell and Carrington, 2015; Fang and Qi, 2016). Thus, 21-nt and 22-nt small RNAs with 5′U are predominantly loaded onto AGO1 (or, in some cases, AGO10), those with 5′C onto AGO5, and those with 5′A onto AGO2 (or, in some cases, AGO7), whereas 24-nt small RNAs with 5′A are preferentially loaded onto AGO4/6/9 clade proteins. It is not known which AGO (if any) is specific for small RNAs with 5′G such as, e.g., abundant 20-nt 5′G-RNAs identified in *Musa acuminata* banana (Rajeswaran et al., 2014b).

Interestingly, infections of *A. thaliana* with cucumber mosaic virus (*Cucumovirus, Bromoviridae*) and turnip mosaic virus (*Potyvirus, Potyviridae*) lead to activation of DCL4- and RDR1-dependent production of endogenous 21-nt siRNAs from multiple protein-coding genes. These virus-activated siRNAs (vasiRNAs) are incorporated into AGO1 and AGO2 RISCs and silence the corresponding host genes post-transcriptionally (Cao et al., 2014). This and other observations indicate that viral infections can dramatically perturb the endogenous plant small RNA pathways.

The small RNA-generating RNAi machinery has evolved in the common ancestor of land plants and green algae (You et al., 2017). DCLs, RDRs, and AGOs have diversified early in the land plants. Interestingly, although the four distinct classes of DCLs that generate miRNAs (DCL1) and various types of endogenous and viral siRNAs (DCL2, DCL3, DCL4) are present in all angiosperms, some (but not all) of the three siRNA-generating DCL genes may be occasionally absent in some species of non-angiosperm lineages (gymnosperms, monilophytes, lycophytes, bryophytes, or ferns), with DCL2 being most frequently lost (Ma et al., 2015; You et al., 2017). Likewise, Pol IV/V or some other components of the angiosperm RdDM machinery appear to be occasionally missing in some species of the non-angiosperm lineages (Ma et al., 2015; You et al., 2017).

## Biogenesis and Function of Viral siRnas

**FIGURE 2:**
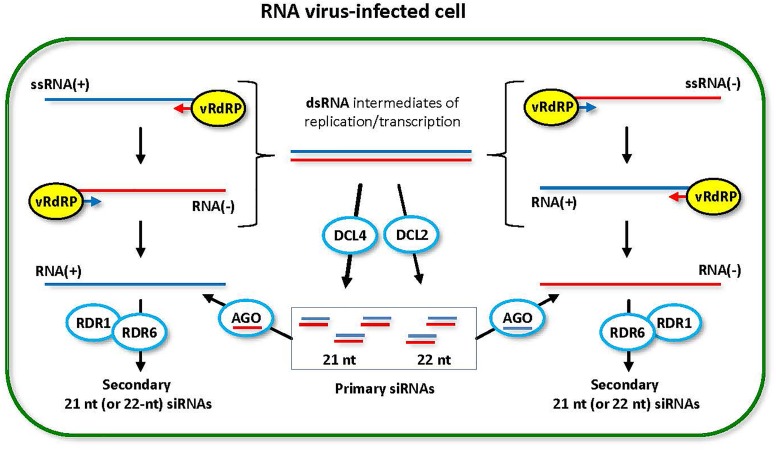
Model for the biogenesis and action of RNA virus-derived siRNAs. During replication (and transcription) of RNA viruses with all types of genomes, (+)ssRNA, dsRNA and (–)ssRNA, viral RNA-dependent RNA polymerase (vRdRP) generates negative strand RNA [RNA(–)] or positive strand RNA [RNA(+)] on the RNA(+) and the RNA(–) templates, respectively. Both processes produce dsRNA intermediates that are recognized by host DCL enzymes. DCL4 is a primary dicer that catalyzes processing of viral dsRNA into 21-nt siRNA duplexes. DCL2 is a secondary dicer that generates 22-nt siRNA duplexes. The resulting primary viral siRNAs are associated with available AGO1/5/10 clade or AGO2/3/7 clade proteins to form RNA-induced silencing complexes containing either sense or antisense strand of the siRNA duplex. Both sense and antisense siRNA-AGO complexes can potentially target the complementary viral RNA(–) and RNA(+), respectively, for cleavage. RDR6 (or RDR1) can potentially convert the cleavage products into dsRNA precursors of secondary viral siRNAs, which reinforces antiviral RNAi. This model was adopted and extended from Pooggin (2016).

**FIGURE 3:**
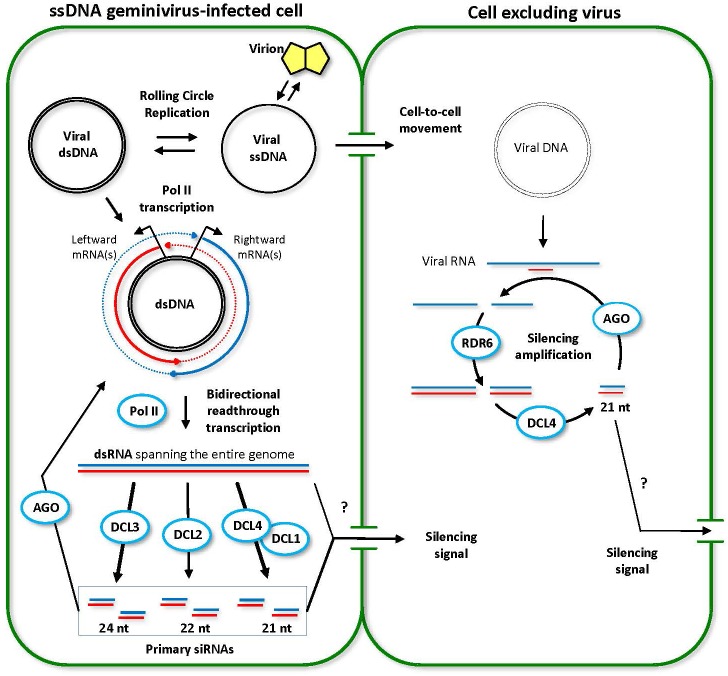
Model for the biogenesis and action of ssDNA geminivirus-derived siRNAs. Two adjoining plant cells are shown schematically. The initially infected cell (left) contains high viral titers. Viral circular single-stranded DNA (ssDNA) released from the virion is converted to double-stranded DNA (dsDNA) that serves as a template for rolling circle replication and Pol II-mediated bidirectional transcription of viral leftward and rightward mRNAs. Double-stranded RNA (dsRNA) molecules representing the entire circular viral genome arise from Pol II-mediated read-through transcription beyond the poly(A) signals in both directions (dotted lines). Every DCL processes the resulting dsRNAs into viral siRNAs of 21 (DCL4 or DCL1), 22 (DCL2) and 24 (DCL3) nucleotides in length. Both viral DNA and viral siRNAs (or long dsRNA) move into the neighboring cell. However, the viral titer remains low, because the RDR6/DCL4 pathway amplifies incoming siRNA signal and digests viral transcripts. This model was adopted and extended from Blevins et al. (2006) and Pooggin (2016).

**FIGURE 4:**
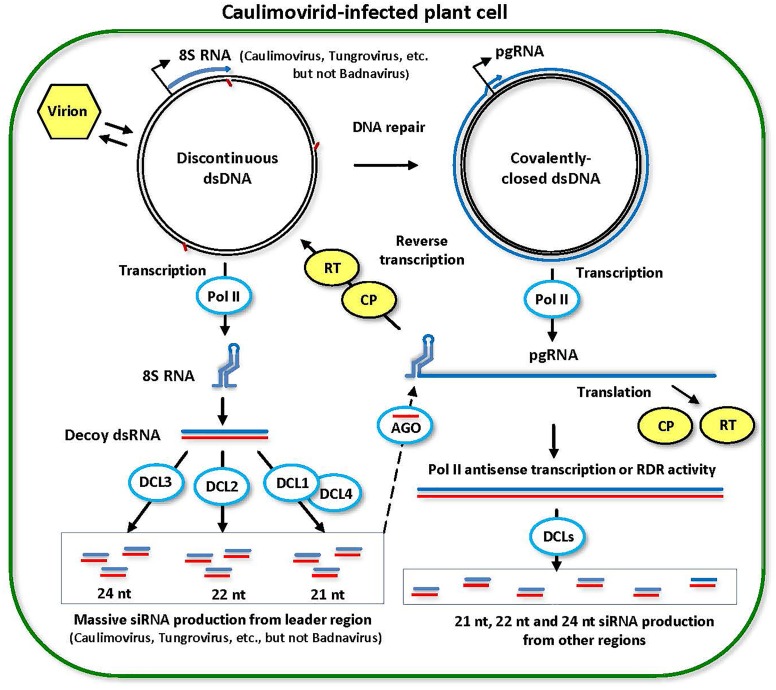
Model for the biogenesis and action of episomal caulimovirid-derived siRNAs. Viral DNA is released from the virion into the nucleoplasm. Gaps in this discontinuous dsDNA left after reverse transcription are repaired by the host repair enzymes to create covalently closed dsDNA. Both repaired and unrepaired forms of viral dsDNA are transcribed by host Pol II. The repaired dsDNA gives rise to pregenomic RNA (pgRNA). Then, pgRNA (and its spiced versions in some genera of *Caulimoviridae*) is transported to cytoplasm to serve as a polycistronic mRNA for coat protein (CP) and reverse transcriptase (RT). pgRNA also serves as a template for reverse transcription by viral RT within a pre-virion made of CP. On the unrepaired dsDNA, abrupt termination of Pol II transcription at the (–)strand DNA gap (the Met-tRNA primer binding site) results in production of 8S RNA, as demonstrated for members of *Caulimovirus* and *Tungrovirus*, or a much shorter RNA in the case of *Badnavirus*. 8S RNA forms a viroid-like secondary structure that is converted into dsRNA (likely by Pol II). The resulting dsRNA serves as a decoy to engage all DCLs in massive production of 21-, 22-, and 24-nt viral siRNAs, which are then associated with AGO family proteins. Stable secondary structure of the pgRNA leader sequence interferes with complementary interaction of viral siRNA-AGO complexes with pgRNA. Less abundant viral siRNAs are also produced from dsRNA precursors representing other regions of the viral genome, which are likely produced by aberrant antisense transcription of viral circular dsDNA mediated by Pol II or from viral sense RNAs by RDR activity. This model was adopted and extended from Blevins et al. (2011) and Pooggin (2016).

The biogenesis and functionalities of 21-nt, 22-nt and 24-nt siRNAs in plant antiviral defenses, dissected mostly in the eudicot *A. thaliana* using its mutants deficient in core components of the RNAi machinery (DCLs, AGOs, RDRs, HEN1, Pol IV/V, etc.) and, to a much lesser extent, in the eudicot *N. benthamiana* and the monocot *Oryza sativa*, have been reviewed in great detail (Pooggin, 2013, 2016; Carbonell and Carrington, 2015). Likewise, viral strategies of suppression and evasion of antiviral RNAi have been reviewed (Pooggin, 2013, 2016, 2017; Csorba et al., 2015). This review illustrates the mechanisms of viral siRNA biogenesis and action in the aforementioned angiosperm plant species infected with RNA and DNA viruses (Figures 2–4) and, based on analysis of the available small RNA-seq data (Supplementary Tables S1, S2 and Supplementary List S1), discusses possible conservation of these mechanisms in other angiosperm and non-angiosperm plants infected with all types of viruses, satellites, and viroids.

### RNA Virus-Derived siRNAs

The hallmark of plant infections with RNA viruses is accumulation of 21-nt and 22-nt viral siRNAs that are generated by DCL4 and DCL2, respectively (Xie et al., 2004; Akbergenov et al., 2006; Blevins et al., 2006; Bouché et al., 2006; Fusaro et al., 2006; Garcia-Ruiz et al., 2010; Wang et al., 2010). RNA viruses with all types of genomes, (+)ssRNA, (-)ssRNA and dsRNA, spawn predominantly 21-nt and 22-nt small RNA (see Supplementary Table S1 and references therein). The relative abundance of 21-nt vs. 22-nt viral siRNAs differs depending on host plant species or other factors (e.g., specific activities of viral silencing suppressors expressed by different types of RNA viruses in targeting different components of RNAi machinery; Csorba et al., 2015) and likely reflects the relative expression levels of DCL4 and DCL2 and/or their relative activities in accessing and processing siRNA precursors (reviewed in Pooggin, 2016). When both DCL4 and DCL2 activities are diminished by knockout or knockdown mutations in *A. thaliana* (Blevins et al., 2006; Bouché et al., 2006; Garcia-Ruiz et al., 2010; Wang et al., 2010) and *N. benthamiana* (Qin et al., 2017), DCL3 takes over to produce RNA virus-derived 24-nt siRNAs, albeit much less efficiently than DCL4 or DCL2, likely because of predominantly nuclear activities of DCL3 in endogenous RdDM and in defense against DNA viruses (discussed below). Nonetheless, in rare cases, 24-nt small RNAs are detectable (together with more abundant 21-nt and 22-nt siRNAs) in wild-type plants infected with some (+)ssRNA and (-)ssRNA viruses in some hosts (see Supplementary Table S1 and references therein).

For all types of RNA viruses [(+)ssRNA, (-)ssRNA, dsRNA], 21-nt and 22-nt small RNAs are produced from both strands of the viral genome without any strong bias in most cases (Supplementary Table S1 and references therein), suggesting that those small RNAs are *bona fide* siRNAs processed by DCL4 and DCL2 from perfect dsRNA precursors in the form of 21-nt and 22-nt duplexes, respectively. However, a strong (+)strand bias of viral small RNAs has been reported for some (+)ssRNA viruses such as cucumber mosaic virus (*Cucumovirus, Bromoviridae*) in tomato and *N. benthamiana* but not *N. tabacum*, and citrus tristeza virus (*Closterovirus, Closteroviridae*) in four out of nine different *Citrus* species hosts, and for some members of *Tombusviridae* and *Virgaviridae* in some hosts. On the other hand, (+)ssRNA viruses of *Tymoviridae* often exhibit (-)strand bias in viral siRNAs (see Supplementary Table S1 and references therein). Interestingly, preferential accumulation of (+)strand-derived small RNAs has been reported for a satellite RNA of beet black scorch virus (*Betanecrovirus, Tombusviridae*) but not for the helper virus itself that spawned 22-nt and 21-nt siRNAs from both strands in *N. benthamiana* (Xu et al., 2016). A strong (+)strand bias for cymbidium ringspot virus (*Tombusvirus, Tombusviridae*)-derived small RNAs in *N. benthamiana* was initially attributed to DCL-mediated processing of secondary structures of single-stranded viral genomic RNA (Molnár et al., 2005; Donaire et al., 2009; Szittya et al., 2010). However, among DCLs only DCL1 has evolved to process hairpin-like secondary structures of miRNA precursor transcripts, while optimal substrates for other DCLs (DCL2, DCL3 and DCL4) are (i) perfect dsRNAs produced by RDR activities (i.e., precursors of tasiRNAs, phasiRNAs, easiRNAs and hcsiRNAs), (ii) perfect dsRNAs formed via annealing sense and antisense transcripts from the same DNA locus (natsiRNAs) or (iii) perfect and near-perfect dsRNAs formed through folding back inverted-repeat transcripts (hpsiRNAs) (reviewed in Borges and Martienssen, 2015). Interestingly, viral small RNAs produced in oilseed rape mosaic virus (*Tobamovirus, Virgaviridae*)-infected *A. thaliana* *dcl2 dcl3 dcl4* triple mutant plants have a strong (+) bias and a much broader size range, unlike those predominantly 21-nt and 22-nt viral siRNAs produced from both strands in control wild-type plants (Malpica-López et al., 2018). This finding has led to a hypothesis that, in conditions when siRNA-generating DCLs are absent or their activities are diminished, DCL1 or other RNase III enzymes (Shamandi et al., 2015; Elvira-Matelot et al., 2016) can access and inefficiently process secondary structures of highly abundant viral genomic and subgenomic RNAs (Malpica-López et al., 2018). A more trivial reason for the observed strand biases could be technical issues with cDNA library preparation protocols as was more recently demonstrated for the aforementioned cymbidium ringspot virus-*N. benthamiana* pathosystem, leading to a conclusion that viral siRNAs are produced from perfect dsRNA precursors rather than viral ssRNA secondary structures and therefore do not exhibit any strand bias (Harris et al., 2015).

Taking together the above described findings as well as other findings and considerations (Carbonell and Carrington, 2015; Pooggin, 2016), a current model for the biogenesis and function of RNA-virus derived siRNAs (Figure 2) states that both DCL4 and DCL2 can access dsRNA intermediates of viral replication and transcription (mediated by viral RNA-dependent RNA polymerase, vRdRP) and process them into 21-nt and 22-nt siRNA duplexes, respectively. These duplexes are loaded onto available AGO proteins from AGO1/5/10 and AGO2/3/7 clades, and one of the duplex strands (sense or antisense) remains in mature RISC complexes. The viral siRNA-AGO RISCs can potentially access cognate viral ssRNA of both sense and antisense polarities through complementary interactions and the AGO slicer activity would result in cleavage of target viral ssRNAs (Carbonell et al., 2012; Schuck et al., 2013; Garcia-Ruiz et al., 2015). The cleavage products can potentially be recognized by available host RDR(s) which would generate dsRNA precursors of secondary viral siRNAs (Figure 2). A proportion of RDR-dependent secondary siRNAs in total viral siRNA population is likely very low, because production of RDR6- and/or RDR1-dependent siRNAs could be revealed only for some RNA virus mutant derivatives lacking functional suppressor proteins [*Cucumovirus* (Wang et al., 2010, 2011) and *Potyvirus* (Garcia-Ruiz et al., 2010), but not *Tobamovirus* (Malpica-López et al., 2018)], whereas wild type virus-derived siRNAs are usually RDR-independent (reviewed in Pooggin, 2016).

The requirements of particular DCLs for biogenesis of viral siRNAs and involvement of other components of the RNAi machinery in biogenesis or action of viral siRNAs have so far been investigated only for (+)ssRNA, ssDNA and dsDNA-RT viruses. Nonetheless, analysis of the viral siRNA size, strand bias and coverage profiles for (-)ssRNA and dsRNA viruses revealed similarities to those of (+)ssRNA viruses (see Supplementary Table S1 and references therein), suggesting that the mechanism presented in Figure 2 likely operates in defense against all types of RNA viruses and in all land plants that possess the depicted components of the RNAi machinery.

Coverage of both strands of an RNA virus genome with siRNA sequencing reads is usually uniform, although local hot and cold spots of siRNAs are evident for all types of RNA viruses, which may reflect DCL preferences for certain dsRNA sequences and/or preferential stabilization of certain siRNA sequences by AGOs. Relative abundance of dsRNAs derived from genomic vs. subgenomic RNAs may also account for non-uniform global distribution of siRNAs along the virus genome (e.g., Ruiz-Ruiz et al., 2011; Turco et al., 2018). In the case of segmented or multipartite viruses with RNA or DNA genomes, differences in relative abundance of siRNAs derived from different genomic components (e.g., Wang et al., 2010; Aregger et al., 2012; Zheng et al., 2017b; Patil and Arora, 2018) may reflect their unequal replication/transcription in a sampled plant tissue. A dsRNA decoy strategy of silencing evasion evolved by pararetroviruses (*Caulimoviridae*; Blevins et al., 2011; Rajeswaran et al., 2014a; discussed below) and perhaps some other viruses can also account for major hotspots of siRNA production.

### Episomal DNA Virus-Derived siRNAs

The hallmark of DNA virus infections of land plants is accumulation of 24-nt viral siRNAs, in addition to 21-nt and 22-nt siRNAs (see Supplementary Table S1 and references therein). So far the biogenesis and function of DNA virus-derived siRNAs have been dissected for the ssDNA virus Cabbage leaf curl virus (bipartite *Begomovirus, Geminiviridae*) and the dsDNA-RT virus Cauliflower mosaic virus (*Caulimovirus, Caulimoviridae*) using combined blot hybridization, small RNA-seq and biochemical approaches in *A. thaliana* and its RNAi-deficient mutant lines (Akbergenov et al., 2006; Blevins et al., 2006, 2011; Shivaprasad et al., 2008; Aregger et al., 2012; Seguin et al., 2014). Likewise, beet curly top virus (*Curtovirus, Geminiviridae*) and its suppressor-deficient mutants have been investigated with a main focus on involvement of components of RdDM and histone modification machinery in antiviral responses (Raja et al., 2014; Jackel et al., 2015, 2016; Coursey et al., 2018), albeit small RNA sequencing was not employed in those studies. For other DNA viruses, much less information is available (reviewed in Pooggin, 2013, 2016). Based on the available evidence, models shown in Figures 3, 4 have been proposed earlier (Pooggin, 2013, 2016) and now updated with a few details and generalized by omitting subgenomic RNAs and specific viral suppressor proteins present in some but on all genera within *Geminiviridae* (Hanley-Bowdoin et al., 2013; Pooggin, 2013, 2016; Ramesh et al., 2017) and *Caulimoviridae* (Pooggin, 2013, 2016; Pooggin and Ryabova, 2018).

In ssDNA geminivirus (*Geminiviridae*)-infected cells (Figure 3), bidirectional transcription mediated by Pol II generates read-through sense and antisense transcripts that form perfect dsRNA precursors of the primary viral siRNAs representing the entire circular virus genome (including the promoter region not covered with viral mRNAs). These dsRNA precursors are processed preferentially by DCL4, DCL2, and DCL3 generating 21-nt, 22-nt, and 24-nt siRNAs, respectively, while DCL1 much less efficiently contributes to generation of 21-nt siRNAs (Blevins et al., 2006; Aregger et al., 2012). All viral siRNA classes likely associate with AGO proteins to target viral transcripts for cleavage, as was deduced from virus-induced gene silencing studies using derivatives of cabbage leaf curl virus in *A. thaliana* single, double, and triple DCL mutants (Blevins et al., 2006; Aregger et al., 2012). AGO-viral siRNA complexes have not been characterized yet for any ssDNA virus. RDR6- and DCL4-dependent secondary siRNAs represent only a minor proportion of 21-nt viral siRNAs, but appear to play a role in cell-to-cell spread and amplification of antiviral silencing (Blevins et al., 2006; Aregger et al., 2012) (Figure 3). Notably, Pol IV, Pol V and RDR2, being involved in endogenous 24-nt siRNA-directed DNA methylation (RdDM) pathways (Borges and Martienssen, 2015; Matzke et al., 2015), are not required for the biogenesis of ssDNA (and dsDNA) virus-derived siRNAs (Blevins et al., 2006; Aregger et al., 2012; Jackel et al., 2016), indicating that episomal DNA viruses evade RdDM and transcriptional silencing of viral genes in the nucleus, despite accumulation of highly abundant 24-nt viral siRNAs (Pooggin, 2013). Consistent with the evasion of transcriptional gene silencing, Pol II bidirectional promotor and terminator regions of bipartite and monopartite *Geminiviridae* are usually devoid of siRNA hotspots and the hotspots of 21-nt, 22-nt, and 24-nt viral siRNAs of both polarities are usually concentrated within viral ORFs (Aregger et al., 2012; Fuentes et al., 2016; see other references in Supplementary Table S1).

In episomal dsDNA-RT virus (*Caulimoviridae*)-infected cells (Figure 4), Pol II monodirectional transcription generates pregenomic RNA (pgRNA) which is transported from nucleus to cytoplasm for translation on ribosomes and reverse transcription mediated by viral reverse transcriptase (RT) in pre-virions composed of viral coat protein (CP) (reviewed in Pooggin and Ryabova, 2018). Discontinuous dsDNA produced via reverse transcription of pgRNA is transported back to nucleus where the discontinuities on both strands are sealed by the host DNA repair machinery, creating covalently closed dsDNA templates for Pol II transcription. If the discontinuity on (-)strand at the Met-tRNA RT primer binding site is not repaired, Pol II produces run-off transcript (so-called 8S RNA) which is converted into perfect dsRNA likely by Pol II-mediated synthesis of complementary RNA, generating dsRNA precursor of viral siRNAs (Figure 4). The latter hypothesis is based on genetic evidence, small RNA-seq and precise transcript mapping for cauliflower mosaic virus (*Caulimovirus*) in *A. thaliana* (Blevins et al., 2011) and small RNA-seq and transcript mapping for RTBV (*Tungrovirus*) in *O. sativa* (Rajeswaran et al., 2014a). The 8S dsRNA of ca. 600 bp serves as a decoy engaging all four DCLs in massive production of 21-nt (DCL1 and DCL4), 22-nt (DCL2), and 24-nt (DCL3) siRNAs (Blevins et al., 2011) (Figure 4), thereby protecting from repressive siRNAs other regions of virus genome (ca. 8 kbp) including the Pol II promotor. The dsRNA precursors of much less abundant 21-nt, 22-nt, and 24-nt siRNAs generated by respective DCLs from non-decoy regions are likely produced via aberrant Pol II mediated antisense transcription on covalently closed circular viral dsDNA, because possible involvement of RDR1, RDR2, RDR6, Pol IV, or Pol V was ruled out for cauliflower mosaic virus in *A. thaliana* (Blevins et al., 2006, 2011). Note that RDR gamma-clade genes present in the *A. thaliana* genome (RDRs 3a, 3b, and 3c) and genomes of other plant species have not been conclusively shown to be involved in biogenesis of endogenous or viral siRNAs, although a tomato Ty-1/Ty-3 RDR gene is implicated in defense against tomato yellow leaf curl virus (*Begomovirus, Geminiviridae*) and amplification of viral siRNAs (Butterbach et al., 2014). Based on a position of the (-)strand discontinuity downstream of the Pol II transcription start site and the viroid-like, strong secondary structure of pgRNA leader, the 8S RNA-like decoy strategy of silencing evasion has been predicted for most but not all genera of *Caulimoviridae* (Pooggin and Ryabova, 2018). In particular, members of the genus *Badnavirus* have the (-)strand discontinuity at a very short distance from the Pol II start site, which may not be compatible with efficient production of a dsRNA decoy (Pooggin and Ryabova, 2018). Indeed, small RNA-seq data obtained for six episomal species of *Badnavirus* in persistently infected *Musa acuminata* banana plants have revealed that hotspots of viral 21-nt, 22-nt, and 24-nt siRNAs are not concentrated in the pgRNA leader region and highly abundant siRNAs occur within ORFs (Rajeswaran et al., 2014b). Similar results have been obtained for other species of the genus *Badnavirus* in raspberry (Kalischuk et al., 2013), pagoda (Wang et al., 2014), taro (Kazmi et al., 2015) and grapevine (Howard and Qiu, 2017). Nonetheless, the Pol II promoter region is devoid of siRNA hotspots in five of the six banana badnaviruses and viral circular dsDNA is not methylated in all the six viruses (Rajeswaran et al., 2014b), indicating that badnaviruses can evade RdDM and transcriptional silencing similar to other genera of *Caulimoviridae* and ssDNA viruses (Pooggin, 2013). Consistent with RdDM evasion, highly abundant 24-nt viral siRNAs accumulating in cauliflower mosaic virus-infected *A. thaliana* were barely detectable on AGO4 protein (using immunoprecipitation followed by RNA blot hybridization), whereas AGO1-associated 21-nt and 22-nt viral siRNAs were readily detectable in this pathosystem (Blevins et al., 2011).

In the case of multipartite circular ssDNA viruses of the family *Nanoviridae*, very little information is available on viral siRNAs (see Supplementary Table S1 and references therein). It is conceivable that all four DCLs including DCL3 are involved in viral siRNA biogenesis and defense against *Nanoviridae*, because of their similarities to *Geminiviridae* in nucleus-based rolling circle replication and to betasatellites of *Geminiviridae* in small size and monodirectional transcription (reviewed in Gronenborn, 2004; Mandal, 2010). Indeed, 21-nt, 22-nt, and 24-nt siRNAs represent both virion and complementary strands of tomato yellow leaf curl China betasatellite (*Tolecusatellitidae*) and cotton leaf curl Multan betasatellite (*Tolecusatellitidae*), similar to their helper viruses of *Geminiviridae* (Yang et al., 2011; Wang J. et al., 2016; see Supplementary Table S1). In the case of *Geminiviridae* and *Nanoviridae* and their satellites (*Alphasatellitidae* and *Tolecusatellitidae*), dsRNA precursors of viral siRNAs may also arise from pervasive sense and antisense transcription mediated by Pol II on multimeric linear dsDNA byproducts of recombination-dependent replication (see Pooggin, 2013).

### Endogenous Viral Element (EVE)-Derived siRNAs

Besides LTR retrotransposons of the families *Metaviridae* (Ty3/Gypsy, ssRNA-RT) and *Pseudoviridae* (Ty1/Copia, ssRNA-RT) that spawn 21-nt, 22-nt, and 24-nt siRNAs as discussed above, EVEs of the families *Caulimoviridae* (dsDNA-RT), *Geminiviridae* (ssDNA) and *Bromoviridae* [(+)ssRNA] have been characterized by small RNA sequencing (see Supplementary Table S1 and references therein).

Among EVEs of *Caulimoviridae*, certain integrants from the genera *Badnavirus, Solendovirus*, and *Petuvirus* can give rise to episomal virus infections upon abiotic stress and/or inter-specific hybridization (Ndowora et al., 1999; Lockhart et al., 2000; Richert-Pöggeler et al., 2003; reviewed in Chabannes and Iskra-Caruana, 2013). In the case of petunia vein clearing virus (PVCV, *Petuvirus*), some of its EVE copies integrated in the genome of *Petunia hybrida* are arranged in tandem repeats, which allows for Pol II transcription of complete pgRNA followed by reverse transcription, giving rise to episomal viral dsDNA (Richert-Pöggeler et al., 2003). The release of episomal PVCV upon abiotic stress correlates with accumulation abundant 21–22-nt viral siRNAs and less abundant 24-nt viral siRNAs, while in the absence of episomal virus, EVE-derived siRNAs are barely detectable by blot hybridization (Noreen et al., 2007). Furthermore, the integrated PVCV sequences are methylated and associated with modified histones, suggesting that epigenetic silencing through RdDM is involved in repression of these EVEs (Richert-Pöggeler et al., 2003; Noreen et al., 2007). Small RNA deep sequencing analysis of *Citrus* sp. has revealed that the *Petuvirus*-like EVEs representing *Citrus endogenous pararetrovirus* spawn predominantly 24-nt siRNAs along with less abundant 21-nt and 22-nt siRNAs (Barrero et al., 2017), further supporting the involvement of RdDM. Likewise, the *Petuvirus*-like EVE sequences at the centromeres of *Fritillaria imperialis* are associated with predominantly 24-nt siRNAs and methylated cytosines at both symmetric (CG, CHG) and asymmetric (CHH) sites, which is a clear signature of RdDM (Becher et al., 2014). Furthermore, EVEs of the genus *Florendovirus* spawn predominantly 24-nt siRNAs of low abundance in *Eucalyptus grandis* (Marcon et al., 2017). 21–24 nt siRNAs derived from both strands of *Florendovirus* EVEs have also been reported for other angiosperms such as grapevine and *Amborella trichopoda* (Geering et al., 2014), although relative levels of 24-nt vs. 21-nt and 22-nt species were not analyzed. Small RNA sequencing has not been reported so far for the infective endogenous caulimovirids from the genus *Badnavirus* integrated in the *Musa balbisiana* diploid B genome or the B genome of *Musa acuminata × balbisiana* hybrid species (Ndowora et al., 1999; Chabannes et al., 2013) and from the genus *Solendovirus* integrated in the genomes of *Nicotiana* sp. (Lockhart et al., 2000). In the case of six episomal viruses of the genus *Badnavirus* in *Musa acuminata* (with triploid AAA genome), virus-derived siRNAs of 21-nt and 22-nt classes are more abundant than those of 24-nt class (Rajeswaran et al., 2014b).

Based on conserved methylation pattern of non-infective *Solendovirus* EVEs in *Nicotiana* *glutinosa* and some other *Nicotiana* species, it has been proposed that those EVEs might confer resistance to exogenous viral counterparts through an epigenetic mechanism (Mette et al., 2002; Gregor et al., 2004). Likewise, endogenous caulimovirid sequences integrated in the genomes of *Solanum* species and up to 83% identical to *Tobacco vein clearing virus* (the infective *Solendovirus* integrated in *Nicotiana* sp.; Lockhart et al., 2000) are methylated at non-symmetrical cytosines (CHH) and associated with predominantly 24-nt siRNAs (as estimated by blot hybridization), which is the hallmark of RdDM and transcriptional silencing (Staginnus et al., 2007). In this and other cases, the silent EVE-derived siRNAs can potentially maintain RdDM (24-nt siRNA), preventing the release of infective EVE copies (if any), and, at the same time, targeting any cognate (incoming) episomal virus in sequence-specific manner at both posttranscriptional (21–22 nt siRNAs) and transcriptional (24-nt siRNA) levels.

Similar to caulimovirids, ssDNA viruses of the family *Geminiviridae* have also been able to get integrated in their host plant genomes (Murad et al., 2004; Filloux et al., 2015; and reference therein). However, unlike endogenous caulimovirids, geminiviral EVEs have not so far been reported to give rise to episomal viruses. Interestingly, geminiviral EVEs integrated in the genomes of water yam (*Dioscorea alata*) and other *Dioscorea* species appear to express the viral replication protein Rep (Filloux et al., 2015). In *D. alata*, one of the two EVEs possessing uninterrupted ORFs for Rep and replication enhancer (but not coat) proteins spawns siRNAs covering both strands of the integrated sequence, with 21-nt and 24-nt species being the first and the second most abundant (Filloux et al., 2015), indicative of post-transcriptional and transcriptional silencing.

**FIGURE 5:**
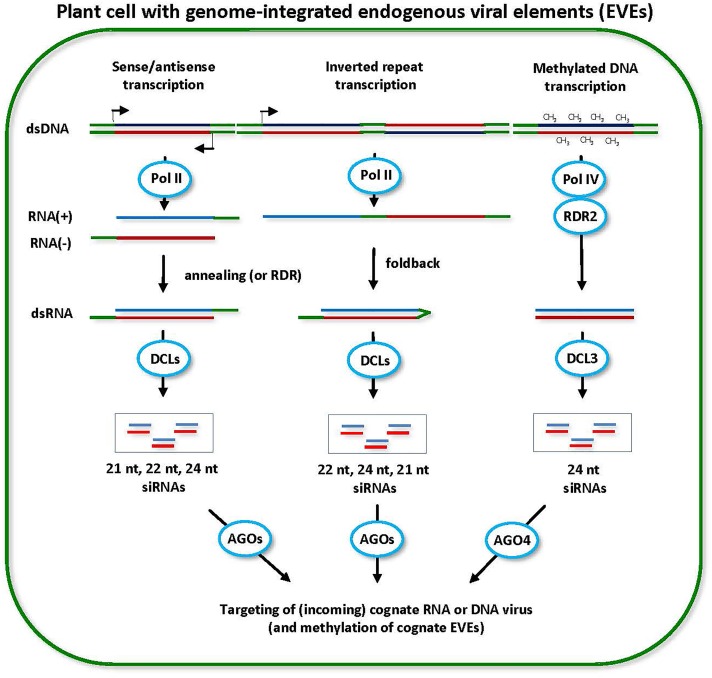
Model for the biogenesis and action of endogenous viral element (EVE)-derived siRNAs. EVEs integrated in the plant genome can potentially be transcribed by Pol II in sense or antisense direction. dsRNA precursors of siRNAs can arise either via annealing of Pol II sense and antisense transcripts produced from the same EVE region or via RDR-mediated synthesis of complementary RNA on sense or antisense transcripts (scheme on the left). The resulting dsRNAs can potentially be processed into 21-nt, 22-nt, and 24-nt siRNAs by DCL4, DCL2, and DCL3, respectively. Likewise, Pol II can potential transcribe the EVE loci containing inverted repeats and the resulting transcript can fold back to form the hairpin dsRNA precursor of siRNAs (scheme in the middle). The hairpin dsRNA is preferentially processed by DCL2 into 22-nt siRNAs and less efficiently by other DCLs into 24-nt (DCL3) and 21-nt (DCL1 or DCL4) siRNAs. Pol II-dependent siRNAs can potentially direct DNA methylation at cognate EVE sequences. The methylated EVE sequences are then transcribed by Pol IV and the resulting transcripts are converted by RDR2 into dsRNA, followed by DCL3 processing into 24-nt siRNAs (scheme on the right). Pol IV-dependent 24-nt siRNAs mediate maintenance of RdDM at cognate EVEs and, in the case of infective EVEs, prevent their release from the genome as an episomal and transmissible virus. All types of siRNAs derived from EVEs can potentially target a cognate episomal virus released from the genome or an incoming exogenous virus.

In rare cases, EVEs representing plant RNA viruses have been reported. Interestingly, an EVE representing RNA1 of the segmented (+)ssRNA virus Cucumber mosaic virus (CMV, *Cucumovirus, Bromoviridae*) is integrated in the genome of *Glycine max* (soybean) (Da Fonseca et al., 2016), while EVEs representing a satellite RNA of this virus are integrated in the genomes of *Nicotiana* species (Zahid et al., 2015). In both cases, these EVEs spawn 24-nt siRNAs, indicative of epigenetic silencing through RdDM (Zahid et al., 2015; Da Fonseca et al., 2016), which is in contrast to the replicating CMV and its satellite that both spawn almost exclusively 21-nt and 22-nt siRNAs in infected host plants (Shimura et al., 2011; Fang et al., 2015; Shen et al., 2015; see other references in Supplementary Table S1). Notably, the CMV RNA1 is integrated in the soybean genome as an inverted repeat of its near-complete 3.4 kb sequence, interrupted with an intervening 0.5 kb sequence of plant origin, and siRNAs are derived exclusively from the invertedly repeated viral sequence, with 22-nt siRNAs being the most abundant, followed by 21-nt and 24-nt classes (Da Fonseca et al., 2016). Based on the similarities to an inverted repeat *IR-71* in *A. thaliana* spawning DCL2-, DCL3-, and DCL4-dependent 22-nt, 24-nt, and 21-nt hpsiRNAs (Henderson et al., 2006) and the integration just downstream of an LTR Copia retrotransposon promotor (Da Fonseca et al., 2016), the CMV RNA1 EVE inverted repeat region is likely transcribed by Pol II and the resulting transcript with invertedly repeated sequence folds back to form a hairpin dsRNA precursor of siRNAs processed by soybean DCL2, DCL3, and DCL4 (Da Fonseca et al., 2016) (Figure 5). It can be further speculated that the Pol IV- and RDR2-dependent pathway generating 24-nt siRNAs is also involved in this case and other cases of EVE loci containing inverted repeats (Figure 5).

As discussed above for transcriptionally active LTR transposons (reviewed in Borges and Martienssen, 2015), the switch from Pol II transcription to Pol IV/Pol V transcription can, in a similar manner, lead to the establishment and maintenance of epigenetic silencing at EVE loci. In the case of EVEs lacking inverted repeats, Pol II transcripts might be recognized and converted to dsRNA by RDR6 (or RDR1), followed by DCL4- and DCL2-mediated production of 21-nt and 22-nt siRNAs and post-transcriptional silencing. Likewise, Pol II bidirectional sense and antisense transcription at some EVE loci might presumably generate dsRNA precursors of 21-nt and 22-nt siRNAs (Figure 5). Potential access of DCL3 to both RDR-dependent and RDR-independent dsRNAs produced at EVE loci in the nucleus would lead to production of 24-nt siRNAs and eventual switch to RdDM and epigenetic silencing maintained by the Pol IV/RDR2/DCL3 pathway (Figure 5). The relative abundance of 21-nt and 22-nt vs. 24-nt siRNAs would reflect relative contribution of post-transcriptional versus transcriptional silencing pathways targeting a particular EVE. In most cases, EVEs are expected to be targeted by the RdDM machinery maintaining epigenetic silencing and generating predominantly 24-nt siRNAs, which would prevent release of those infective EVEs described above for *Caulimoviridae*.

Virus infections (like other stress factors) and especially activities of viral suppressor proteins might potentially perturb the RdDM machinery and lead to activation and release of transposons and infective EVEs which would contribute to disease severity. For the purpose of virus diagnostics and virome reconstruction the distinguishing features of episomal dsDNA and ssDNA viruses would be (i) high abundance viral siRNAs of both 21–22 nt and 24-nt classes, reflecting activities of the components of both post-transcriptional and transcriptional silencing machineries, and (ii) coverage of the entire circular viral genome sequences with siRNAs with characteristic hotspots within ORFs rather than Pol II promoter regions (as discussed above; also see Supplementary Table S1 and references therein for episomal infections of *Caulimoviridae* and *Geminiviridae*). The distinguishing features of silent EVEs of *Caulimoviridae* and *Geminiviridae* as well as RNA viruses would be predominantly 24-nt siRNAs of lower abundance which might be more broadly distributed along the reference genomes of their episomal counterparts and which would represent only those sequences of a reference episomal virus genome that are integrated in the host genome. Furthermore, siRNAs derived from inverted repeats of EVEs (if any) are expected to be more abundant and likely enriched in 22-nt and 21-nt classes in addition to 24-nt class. Further research using small RNA deep sequencing is needed to investigate size, 5′-nt identity, polarity, and hotspot profiles of EVE-derived siRNAs as well as the mechanisms of epigenetic silencing and activation of the infective EVEs giving rise to episomal viruses.

Putative protective potential of EVE-derived siRNAs against cognate virus infections proposed earlier (Mette et al., 2002; Bertsch et al., 2009; Da Fonseca et al., 2016) and discussed above is further supported by the studies of transgenic plants expressing siRNAs from hairpin RNA transgenes carrying invertedly repeated viral sequences. Such RNAi-transgenic plants are resistant and, in some cases, immune to infection with the corresponding virus, and the immunity correlates with transgenic production of phloem-mobile 24-nt siRNAs (reviewed in Pooggin, 2017). Interestingly, abundance of transgene-derived 24-nt siRNAs appears to correlate with immunity or increased resistance not only to DNA viruses such as tomato yellow leaf curl virus (*Begomovirus, Geminiviridae*) in tomato (Leibman et al., 2015; Fuentes et al., 2016; see Pooggin, 2017), but also RNA viruses such as zucchini yellow mosaic virus (*Potyvirus, Potyviridae*) in cucurbits (Leibman et al., 2011), prunus necrotic ring spot virus (*Ilarvirus, Bromoviridae*) in sweet cherry (Zhao and Song, 2014a,b) and lettuce infectious yellows virus (*Crinivirus, Closteroviridae*) in *N. benthamiana* (Qiao et al., 2018).

### Viroid-Derived siRNAs

The hallmark of viroids of the family *Avsunviroidae* that replicate in chloroplasts is accumulation of almost exclusively 21-nt and 22-nt siRNAs, whereas the hallmark of viroids of the family *Pospiviroidae* that replicate in nuclei is accumulation of 24-nt siRNAs, in addition to 21-nt and 22-nt siRNAs (see Supplementary Table S2 and references therein). For both types of viroids, siRNAs cover both strands of the entire circular viroid genome, suggesting that DCLs recognize and process perfect dsRNA intermediates of viroid replication or dsRNAs generated by RDR activities.

Because RNAi activity was not reported in chloroplasts or other plastids, Di Serio et al. (2009) have proposed that peach latent mosaic viroid (*Pelamoviroid, Avsunviroidae*)-derived 21-nt siRNAs and less abundant 22-nt siRNAs are generated by DCL4 and DCL2, respectively, in the cytoplasm of infected peach cells. Likewise, DCL4 and DCL2 have been proposed to mediate the biogenesis of predominantly 21-nt siRNAs and less abundant 22-nt siRNAs derived from both strands of apple hammerhead viroid (tentative *Pelamoviroid*) in apple (Zhang et al., 2014). The mechanism of biogenesis of chloroplastic viroid-derived siRNAs remains to be further investigated.

In the case of nuclear viroids, their replication in the nucleus via an asymmetric rolling circle mechanism (likely mediated by Pol II; reviewed in Katsarou et al., 2015; Rao and Kalantidis, 2015) generates perfect dsRNA replicative intermediates that might be accessed by DCL3 to generate 24-nt siRNAs. RNA blot hybridization analysis of *N. benthamiana* and its DCL knockdown lines infected with potato spindle tuber viroid (PSTVd, *Pospiviroid, Pospiviroidae*) has revealed that 21-nt, 22-nt, and 24-nt viroid siRNAs are generated by DCL4, DCL2 and DCL3, respectively (Dadami et al., 2013; Katsarou et al., 2016). DCL1 may also contribute to the biogenesis of 21-nt viroid siRNAs, as evident in *dcl2 dcl3 dcl4* triple knockdown lines (Katsarou et al., 2016). This is reminiscent of DNA viruses targeted by all four DCLs in *A. thaliana*, with both DCL4 and DCL1 contributing to production of 21-nt viral siRNAs (Blevins et al., 2006, 2011; Aregger et al., 2012). Interestingly, DCL1 plays a major role in processing 21-nt siRNAs from the dsRNA decoy likely generated by Pol II from viroid-like 8S RNA of cauliflower mosaic virus (Blevins et al., 2011) (Figure 4). It has been demonstrated using small RNA sequencing and blot hybridization that RDR6 is not required for the biogenesis of PSTVd-derived siRNAs of any size-class, although viroid accumulation was increased at the earlier (but not late) time point in *N. benthamiana* *rdr6* knockdown plants (Di Serio et al., 2010). This supports the hypothesis that dsRNA intermediates of viroid replication are processed by DCLs. PSTVd-derived siRNAs are sorted by multiple AGOs based on 5′-nt identity and size, with AGO1, AGO2, and AGO3 being preferentially associated with 21-nt and 22-nt siRNAs, while AGO4, AGO5, and AGO9 additionally bound to 24-nt siRNAs, as demonstrated by immunoprecipitation of *A. thaliana* AGOs agro-expressed in *N. benthamiana* followed by small RNA sequencing (Minoia et al., 2014). This indicates that viroid siRNAs form active RISCs that can potentially mediate both post-transcriptional and transcriptional silencing. Indeed, siRNAs derived from both nuclear and chloroplastic viroids can direct silencing of the host plant mRNAs through sequence-specific cleavage and degradation (Navarro et al., 2012; Adkar-Purushothama et al., 2015, 2018; Adkar-Purushothama and Perreault, 2018), although viroids can also indirectly impact on the host gene expression at both post-transcriptional and transcriptional levels (Castellano et al., 2015; Tsushima et al., 2015; Torchetti et al., 2016; Zheng et al., 2017c). Notably, nuclear viroids can also trigger *de novo* RNA-directed DNA methylation of the transgenes containing viroid sequences (Wassenegger et al., 1994; Dalakouras et al., 2016). Whether or not viroid-derived 24-nt siRNAs are involved in this RdDM process remains unclear (Dalakouras et al., 2016).

Interestingly, *Dianthus caryophyllus retroviroid*-like element integrated in the genome of carnation could be identified by small RNA sequencing and assembly (Verdin et al., 2017), suggesting that it is potentially silenced epigenetically like other EVEs.

## Concluding Remarks and Outlook

As described above, all families of land plant viruses and viroids spawn characteristic small RNAs whose deep sequencing and bioinformatics analysis allows for virus identification and virome reconstruction. The small RNA size, polarity and hotspot profiles are indicative of virus interactions with components of the plant small RNA-generating RNAi machinery and allow to distinguish between exogenous viruses and silent EVEs, some of which can potentially be released from the plant genome to establish systemic and transmissible infections. Based on the conservation of the small RNA-generating RNAi machinery in all land plants and eukaryotic algae, the small RNA-omics approach is universal for diagnostics of known viruses, identification of viruses or virus-like agents associated with diseases of unknown etiology, and exhaustive reconstruction of viromes of any plant species. Specialized but partially redundant functions of DCLs, RDRs, and AGOs in both endogenous and antiviral siRNA pathways revealed in the model plant *A. thaliana* imply that in those species of land plants that lack some of the paralogs of the DCL, RDR, or AGO family genes (Ma et al., 2015; You et al., 2017), other remaining paralogs can still function in defense against viruses, EVEs and LTR retrotransposons. Further research of non-model plants is needed to characterize components of the RNAi machinery and their roles in biogenesis and function of viral siRNAs. This research will certainly be facilitated by CRISPR-based gene editing technology, as for example recently implemented in tomato to reveal the role of one of the four paralogs of DCL2 in 22-nt siRNA biogenesis and antiviral defense (Wang T. et al., 2018). As mentioned above, combination of small RNA sequencing with direct sequencing of long RNA and DNA molecules using PacBio and Nanopore technologies should enable reconstruction of complete viral genomes and discovery of their mutant and recombinant variants in mixed virome quasispecies. The successful application of small RNA sequencing for reconstruction of viruses from the old seed and dried plant tissues described above opens up great opportunities for more exhaustive virome reconstruction from herbaria and other collections of dried plant materials used in paleovirology.

Notably, viral siRNA size and hotspot profiles of those propagative RNA viruses that replicate both in the plant and the insect vector differ substantially (Xu et al., 2012; Fletcher et al., 2016; de Haro et al., 2017). Unlike plants, insects possess Dicer-independent, PIWI-interacting RNA (piRNA)-generating machinery that may contribute to the antiviral defenses mediated by Dicer-dependent siRNA-generating machinery (Mongelli and Saleh, 2016; Sattar and Thompson, 2016). It will be interesting to investigate if transmission of propagative and non-propagative plant viruses by their insect or other eukaryotic vectors (Hull, 2014) is regulated by the vector siRNA and/or piRNA pathways.

## Author Contributions

MP wrote the manuscript, prepared the figures, and the Supplementary Tables and Lists.

## Conflict of Interest Statement

The author declares that the research was conducted in the absence of any commercial or financial relationships that could be construed as a potential conflict of interest.

## References

[B1] Adkar-Purushothama C. R. Brosseau C. Giguère T. Sano T. Moffett P. Perreault J. P. (2015). Small RNA derived from the virulence modulating region of the *Potato spindle tuber viroid* Silences *callose synthase* genes of tomato plants. *Plant Cell* 27 2178–2194. 10.1105/tpc.15.00523 26290537 PMC4568511

[B2] Adkar-Purushothama C. R. Perreault J. P. (2018). Alterations of the viroid regions that interact with the host defense genes attenuate viroid infection in host plant. *RNA Biol.* 22 1–12. 10.1080/15476286.2018.1462653 29683389 PMC6161676

[B3] Adkar-Purushothama C. R. Sano T. Perreault J. P. (2018). Viroid derived small RNA induces early flowering in tomato plants by RNA silencing. *Mol. Plant Pathol.* 19 2446–2458. 10.1111/mpp.12721 30011126 PMC6637976

[B4] Aguiar E. R. Olmo R. P. Paro S. Ferreira F. V. de Faria I. J. Todjro Y. M. (2015). Sequence-independent characterization of viruses based on the pattern of viral small RNAs produced by the host. *Nucleic Acids Res.* 43 6191–6206. 10.1093/nar/gkv587 26040701 PMC4513865

[B5] Akbergenov R. Si-Ammour A. Blevins T. Amin I. Kutter C. Vanderschuren H. (2006). Molecular characterization of geminivirus-derived small RNAs in different plant species. *Nucleic Acids Res.* 34 462–471. 10.1093/nar/gkj447 16421273 PMC1342034

[B6] Alabi O. J. Zheng Y. Jagadeeswaran G. Sunkar R. Naidu R. A. (2012). High-throughput sequence analysis of small RNAs in grapevine (*Vitis vinifera* L.) affected by grapevine leafroll disease. *Mol. Plant Pathol.* 13 1060–1076. 10.1111/j.1364-3703.2012.00815.x 22827483 PMC6638782

[B7] Alejandri-Ramírez N. D. Chávez-Hernández E. C. Contreras-Guerra J. L. Reyes J. L. Dinkova T. D. (2018). Small RNA differential expression and regulation in Tuxpeño maize embryogenic callus induction and establishment. *Plant Physiol. Biochem.* 122 78–89. 10.1016/j.plaphy.2017.11.013 29197696

[B8] Aregger M. Borah B. K. Seguin J. Rajeswaran R. Gubaeva E. G. Zvereva A. S. (2012). Primary and secondary siRNAs in geminivirus-induced gene silencing. *PLoS Pathog.* 8:e1002941. 10.1371/journal.ppat.1002941 23028332 PMC3460622

[B9] Barrero R. A. Napier K. R. Cunnington J. Liefting L. Keenan S. Frampton R. A. (2017). An internet-based bioinformatics toolkit for plant biosecurity diagnosis and surveillance of viruses and viroids. *BMC Bioinformatics* 18:26. 10.1186/s12859-016-1428-4 28077064 PMC5225587

[B10] Becher H. Ma L. Kelly L. J. Kovarik A. Leitch I. J. Leitch A. R. (2014). Endogenous pararetrovirus sequences associated with 24 nt small RNAs at the centromeres of *Fritillaria imperialis* L. (Liliaceae), a species with a giant genome. *Plant J.* 80 823–833. 10.1111/tpj.12673 25230921

[B11] Bertsch C. Beuve M. Dolja V. V. Wirth M. Pelsy F. Herrbach E. (2009). Retention of the virus-derived sequences in the nuclear genome of grapevine as a potential pathway to virus resistance. *Biol. Direct* 4:21. 10.1186/1745-6150-4-21 19558678 PMC2714080

[B12] Blevins T. Podicheti R. Mishra V. Marasco M. Wang J. Rusch D. (2015). Identification of Pol IV and RDR2-dependent precursors of 24 nt siRNAs guiding de novo DNA methylation in Arabidopsis. *eLife* 4:e09591. 10.7554/eLife.09591 26430765 PMC4716838

[B13] Blevins T. Rajeswaran R. Aregger M. Borah B. K. Schepetilnikov M. Baerlocher L. (2011). Massive production of small RNAs from a non-coding region of *Cauliflower mosaic virus* in plant defense and viral counter-defense. *Nucleic Acids Res.* 39 5003–5014. 10.1093/nar/gkr119 21378120 PMC3130284

[B14] Blevins T. Rajeswaran R. Shivaprasad P. V. Beknazariants D. Si-Ammour A. Park H. S. (2006). Four plant Dicers mediate viral small RNA biogenesis and DNA virus induced silencing. *Nucleic Acids Res.* 34 6233–6246. 10.1093/nar/gkl886 17090584 PMC1669714

[B15] Blouin A. G. Chooi K. M. Warren B. Napier K. R. Barrero R. A. MacDiarmid R. M. (2018a). Grapevine virus I, a putative new vitivirus detected in co-infection with grapevine virus G in New Zealand. *Arch. Virol.* 163 1371–1374. 10.1007/s00705-018-3738-5 29392493

[B16] Blouin A. G. Keenan S. Napier K. R. Barrero R. A. MacDiarmid R. M. (2018b). Identification of a novel vitivirus from grapevines in New Zealand. *Arch. Virol.* 163 281–284. 10.1007/s00705-017-3581-0 29026999

[B17] Bolduc F. Hoareau C. St-Pierre P. Perreault J. P. (2010). In-depth sequencing of the siRNAs associated with peach latent mosaic viroid infection. *BMC Mol. Biol.* 11:16. 10.1186/1471-2199-11-16 20158907 PMC2830927

[B18] Boonham N. Kreuze J. Winter S. van der Vlugt R. Bergervoet J. Tomlinson J. (2014). Methods in virus diagnostics: from ELISA to next generation sequencing. *Virus Res.* 186 20–31. 10.1016/j.virusres.2013.12.007 24361981

[B19] Borges F. Martienssen R. A. (2015). The expanding world of small RNAs in plants. *Nat. Rev. Mol. Cell Biol.* 16 727–741. 10.1038/nrm4085 26530390 PMC4948178

[B20] Bouché N. Lauressergues D. Gasciolli V. Vaucheret H. (2006). An antagonistic function for *Arabidopsis* DCL2 in development and a new function for DCL4 in generating viral siRNAs. *EMBO J.* 25 3347–3356. 10.1038/sj.emboj.7601217 16810317 PMC1523179

[B21] Butterbach P. Verlaan M. G. Dullemans A. Lohuis D. Visser R. G. Bai Y. (2014). Tomato yellow leaf curl virus resistance by Ty-1 involves increased cytosine methylation of viral genomes and is compromised by cucumber mosaic virus infection. *Proc. Natl. Acad. Sci. U.S.A.* 111 12942–12947. 10.1073/pnas.1400894111 25136118 PMC4156758

[B22] Cao M. Du P. Wang X. Yu Y. Q. Qiu Y. H. Li W. (2014). Virus infection triggers widespread silencing of host genes by a distinct class of endogenous siRNAs in *Arabidopsis*. *Proc. Natl. Acad. Sci. U.S.A.* 111 14613–14618. 10.1073/pnas.1407131111 25201959 PMC4209997

[B23] Cao M. Lan P. Li F. Abad J. Zhou C. Li R. (2017). Genome characterization of sweet potato symptomless virus 1: a mastrevirus with an unusual nonanucleotide sequence. *Arch. Virol.* 162 2881–2884. 10.1007/s00705-017-3396-z 28497216

[B24] Carbonell A. Carrington J. C. (2015). Antiviral roles of plant ARGONAUTES. *Curr. Opin. Plant Biol.* 27 111–117. 10.1016/j.pbi.2015.06.013 26190744 PMC4618181

[B25] Carbonell A. Fahlgren N. Garcia-Ruiz H. Gilbert K. B. Montgomery T. A. Nguyen T. (2012). Functional analysis of three *Arabidopsis* ARGONAUTES using slicer-defective mutants. *Plant Cell* 24 3613–3629. 10.1105/tpc.112.099945 23023169 PMC3480291

[B26] Carvajal-Yepes M. Olaya C. Lozano I. Cuervo M. Castaño M. Cuellar W. J. (2014). Unraveling complex viral infections in cassava (*Manihot esculenta* Crantz) from Colombia. *Virus Res.* 186 76–86. 10.1016/j.virusres.2013.12.011 24374265

[B27] Castellano M. Martinez G. Pallás V. Gómez G. (2015). Alterations in host DNA methylation in response to constitutive expression of Hop stunt viroid RNA in *Nicotiana benthamiana* plants. *Plant Pathol.* 64 1247–1257. 10.1111/ppa.12358

[B28] Chabannes M. Baurens F. C. Duroy P. O. Bocs S. Vernerey M. S. Rodier-Goud M. (2013). Three infectious viral species lying in wait in the banana genome. *J. Virol.* 87 8624–8637. 10.1128/JVI.00899-13 23720724 PMC3719817

[B29] Chabannes M. Iskra-Caruana M. L. (2013). Endogenous pararetroviruses–a reservoir of virus infection in plants. *Curr. Opin. Virol.* 3 615–620. 10.1016/j.coviro.2013.08.012 24035682

[B30] Chai A. Chen L. D. Shi Y. Xie X. Li B. Zhang X. (2018). First report of a mixed infection of *Tomato mottle mosaic virus* and Tobacco mild green mosaic virus on Eggplants in China. *Plant Dis.* 10.1094/PDIS-04-18-0686-PDN

[B31] Chiumenti M. Mohorianu I. Roseti V. Saldarelli P. Dalmay T. Minafra A. (2016a). High-throughput-sequencing-based identification of a *Grapevine fanleaf virus* satellite RNA in *Vitis vinifera*. *Arch. Virol.* 161 1401–1403. 10.1007/s00705-016-2776-0 26873812

[B32] Chiumenti M. Morelli M. De Stradis A. Elbeaino T. Stavolone L. Minafra A. (2016b). Unusual genomic features of a badnavirus infecting mulberry. *J. Gen. Virol.* 97 3073–3087. 10.1099/jgv.0.000600 27604547

[B33] Coursey T. Regedanz E. Bisaro D. M. (2018). Arabidopsis RNA polymerase v mediates enhanced compaction and silencing of geminivirus and transposon chromatin during host recovery from infection. *J. Virol.* 92:e01320-17. 10.1128/JVI.01320-17 29321305 PMC5972885

[B34] Creasey K. M. Zhai J. Borges F. Van Ex F. Regulski M. Meyers B. C. (2014). miRNAs trigger widespread epigenetically activated siRNAs from transposons in *Arabidopsis*. *Nature* 508 411–415. 10.1038/nature13069 24670663 PMC4074602

[B35] Csorba T. Kontra L. Burgyán J. (2015). viral silencing suppressors: tools forged to fine-tune host-pathogen coexistence. *Virology* 479–480, 85–103. 10.1016/j.virol.2015.02.028 25766638

[B36] Cuellar W. J. Cruzado R. K. Fuentes S. Untiveros M. Soto M. Kreuze J. F. (2011). Sequence characterization of a Peruvian isolate of *Sweet potato chlorotic stunt virus*: further variability and a model for p22 acquisition. *Virus Res.* 157 111–115. 10.1016/j.virusres.2011.01.010 21262288 PMC3125117

[B37] Cuellar W. J. Galvez M. Fuentes S. Tugume J. Kreuze J. (2015). Synergistic interactions of begomoviruses with *Sweet potato chlorotic stunt virus* (genus *Crinivirus*) in sweet potato (*Ipomoea batatas* L.). *Mol. Plant Pathol.* 16 459–471. 10.1111/mpp.12200 25187172 PMC6638456

[B38] Czotter N. Molnar J. Szabó E. Demian E. Kontra L. Baksa I. (2018). NGS of virus-derived small RNAs as a diagnostic method used to determine viromes of hungarian vineyards. *Front. Microbiol.* 9:122 10.3389/fmicb.2018.00122

[B39] Da Fonseca G. C. de Oliveira L. F. V. de Morais G. L. Abdelnor R. V. Nepomuceno A. L. Waterhouse P. M. (2016). Unusual RNA plant virus integration in the soybean genome leads to the production of small RNAs. *Plant Sci.* 246 62–69. 10.1016/j.plantsci.2016.01.011 26993236

[B40] Dadami E. Boutla A. Vrettos N. Tzortzakaki S. Karakasilioti I. Kalantidis K. (2013). DICER-LIKE 4 but not DICER-LIKE 2 may have a positive effect on *Potato spindle tuber viroid* accumulation in *Nicotiana benthamiana*. *Mol. Plant.* 6 232–234. 10.1093/mp/sss118 23100483

[B41] Dalakouras A. Dadami E. Wassenegger M. Krczal G. Wassenegger M. (2016). RNA-directed DNA methylation efficiency depends on trigger and target sequence identity. *Plant J.* 87 202–214. 10.1111/tpj.13193 27121647

[B42] de Haro L. A. Dumón A. D. Mattio M. F. Argüello Caro E. B. Llauger G. Zavallo D. (2017). *Mal de Río Cuarto Virus* infection triggers the production of distinctive viral-derived siRNA profiles in wheat and its planthopper vector. *Front. Plant Sci.* 8:766. 10.3389/fpls.2017.00766 28539933 PMC5423983

[B43] De Souza J. Fuentes S. Savenkov E. I. Cuellar W. Kreuze J. F. (2013). The complete nucleotide sequence of sweet potato C6 virus: a carlavirus lacking a cysteine-rich protein. *Arch. Virol.* 158 1393–1396. 10.1007/s00705-013-1614-x 23358614

[B44] Di Serio F. Gisel A. Navarro B. Delgado S. Martínez de Alba A. E. Donvito G. (2009). Deep sequencing of the small RNAs derived from two symptomatic variants of a chloroplastic viroid: implications for their genesis and for pathogenesis. *PLoS One* 4:e7539. 10.1371/journal.pone.0007539 19847296 PMC2760764

[B45] Di Serio F. Martínez de Alba A. E. Navarro B. Gisel A. Flores R. (2010). RNA-dependent RNA polymerase 6 delays accumulation and precludes meristem invasion of a viroid that replicates in the nucleus. *J. Virol.* 84 2477–2489. 10.1128/JVI.02336-09 20015979 PMC2820905

[B46] Diop S. I. Geering A. D. W. Alfama-Depauw F. Loaec M. Teycheney P. Y. Maumus F. (2018). Tracheophyte genomes keep track of the deep evolution of the *Caulimoviridae*. *Sci. Rep.* 8:572. 10.1038/s41598-017-16399-x 29330451 PMC5766536

[B47] Donaire L. Ayllón M. A. (2017). Deep sequencing of mycovirus-derived small RNAs from *Botrytis* species. *Mol. Plant Pathol.* 18 1127–1137. 10.1111/mpp.12466 27578449 PMC6638239

[B48] Donaire L. Wang Y. Gonzalez-Ibeas D. Mayer K. F. Aranda M. A. Llave C. (2009). Deep-sequencing of plant viral small RNAs reveals effective and widespread targeting of viral genomes. *Virology* 392 203–214. 10.1016/j.virol.2009.07.005 19665162

[B49] Eichmeier A. Komínková M. Komínek P. Baránek M. (2016). Comprehensive virus detection using next generation sequencing in grapevine vascular tissues of plants obtained from the wine regions of bohemia and moravia (Czech Republic). *PLoS One* 11:e0167966. 10.1371/journal.pone.0167966 27959951 PMC5154529

[B50] Elvira-Matelot E. Hachet M. Shamandi N. Comella P. Sáez-Vásquez J. Zytnicki M. (2016). Arabidopsis RNASE THREE LIKE2 modulates the expression of protein-coding genes via 24-nucleotide small interfering RNA-directed DNA methylation. *Plant Cell* 28 406–425. 10.1105/tpc.15.00540 26764378 PMC4790866

[B51] Fang X. Qi Y. (2016). RNAi in plants: an argonaute-centered view. *Plant Cell* 28 272–285. 10.1105/tpc.15.00920 26869699 PMC4790879

[B52] Fang Y. Y. Smith N. A. Zhao J. H. Lee J. R. Guo H. S. Wang M. B. (2015). Cloning and profiling of small RNAs from cucumber mosaic virus satellite RNA. *Methods Mol. Biol.* 1236 99–109. 10.1007/978-1-4939-1743-3_9 25287499

[B53] Filloux D. Murrell S. Koohapitagtam M. Golden M. Julian C. Galzi S. (2015). The genomes of many yam species contain transcriptionally active endogenous geminiviral sequences that may be functionally expressed. *Virus Evol.* 1:vev002. 10.1093/ve/vev002 27774276 PMC5014472

[B54] Fletcher S. J. Shrestha A. Peters J. R. Carroll B. J. Srinivasan R. Pappu H. R. (2016). The *Tomato Spotted Wilt Virus* genome is processed differentially in its plant host *Arachis hypogaea* and its thrips vector *Frankliniella fusca*. *Front. Plant Sci.* 7:1349. 10.3389/fpls.2016.01349 27656190 PMC5013717

[B55] Fuentes A. Carlos N. Ruiz Y. Callard D. Sánchez Y. Ochagavía M. E. (2016). Field trial and molecular characterization of RNAi-transgenic tomato plants that exhibit resistance to tomato yellow leaf curl geminivirus. *Mol. Plant Microbe Interact.* 29 197–209. 10.1094/MPMI-08-15-0181-R 26713353

[B56] Fung E. Hill K. Hogendoorn K. Glatz R. V. Napier K. R. Bellgard M. I. (2018). De novo assembly of honey bee RNA viral genomes by tapping into the innate insect antiviral response pathway. *J. Invertebr. Pathol.* 152 38–47. 10.1016/j.jip.2018.01.002 29378202

[B57] Fusaro A. F. Matthew L. Smith N. A. Curtin S. J. Dedic-Hagan J. Ellacott G. A. (2006). RNA interference-inducing hairpin RNAs in plants act through the viral defence pathway. *EMBO Rep.* 7 1168–1175. 10.1038/sj.embor.7400837 17039251 PMC1679793

[B58] Garcia-Ruiz H. Carbonell A. Hoyer J. S. Fahlgren N. Gilbert K. B. Takeda A. (2015). Roles and programming of Arabidopsis ARGONAUTE proteins during *Turnip mosaic virus* infection. *PLoS Pathog.* 11:e1004755. 10.1371/journal.ppat.1004755 25806948 PMC4373807

[B59] Garcia-Ruiz H. Takeda A. Chapman E. J. Sullivan C. M. Fahlgren N. Brempelis K. J. (2010). *Arabidopsis* RNA-dependent RNA polymerases and dicer-like proteins in antiviral defense and small interfering RNA biogenesis during *Turnip Mosaic Virus* infection. *Plant Cell* 22 481–496. 10.1105/tpc.109.073056 20190077 PMC2845422

[B60] Geering A. D. Maumus F. Copetti D. Choisne N. Zwickl D. J. Zytnicki M. (2014). Endogenous florendoviruses are major components of plant genomes and hallmarks of virus evolution. *Nat. Commun.* 5:5269. 10.1038/ncomms6269 25381880 PMC4241990

[B61] Ghildiyal M. Zamore P. D. (2009). Small silencing RNAs: an expanding universe. *Nat. Rev. Genet.* 10 94–108. 10.1038/nrg2504 19148191 PMC2724769

[B62] Giampetruzzi A. Roumi V. Roberto R. Malossini U. Yoshikawa N. La Notte P. (2012). A new grapevine virus discovered by deep sequencing of virus- and viroid-derived small RNAs in Cv Pinot gris. *Virus Res.* 163 262–268. 10.1016/j.virusres.2011.10.010 22036729

[B63] Glasa M. Predajňa L. Komínek P. Nagyová A. Candresse T. Olmos A. (2014). Molecular characterization of divergent grapevine Pinot gris virus isolates and their detection in Slovak and Czech grapevines. *Arch. Virol.* 159 2103–2107. 10.1007/s00705-014-2031-5 24599565

[B64] Glasa M. Predajňa L. Šoltys K. Sabanadzovic S. Olmos A. (2015). Detection and molecular characterisation of *Grapevine Syrah virus*-1 isolates from Central Europe. *Virus Genes* 51 112–121. 10.1007/s11262-015-1201-1 25940164

[B65] Gong Z. Han G. Z. (2018). Euphyllophyte paleoviruses illuminate hidden diversity and macroevolutionary mode of Caulimoviridae. *J. Virol.* 92:e02043-17. 10.1128/JVI.02043-17 29491164 PMC5923073

[B66] Gregor W. Mette M. F. Staginnus C. Matzke M. A. Matzke A. J. (2004). A distinct endogenous pararetrovirus family in *Nicotiana tomentosiformis*, a diploid progenitor of polyploid tobacco. *Plant Physiol.* 134 1191–1199. 10.1104/pp.103.031112 14988473 PMC389943

[B67] Gronenborn B. (2004). Nanoviruses: genome organisation and protein function. *Vet. Microbiol.* 98 103–109. 10.1016/j.vetmic.2003.10.015 14741122

[B68] Hamilton A. J. Baulcombe D. C. (1999). A species of small antisense RNA in posttranscriptional gene silencing in plants. *Science* 286 950–952. 10.1126/science.286.5441.950 10542148

[B69] Hanley-Bowdoin L. Bejarano E. R. Robertson D. Mansoor S. (2013). Geminiviruses: masters at redirecting and reprogramming plant processes. *Nat. Rev. Microbiol.* 11 777–788. 10.1038/nrmicro3117 24100361

[B70] Harris C. J. Molnar A. Müller S. Y. Baulcombe D. C. (2015). FDF-PAGE: a powerful technique revealing previously undetected small RNAs sequestered by complementary transcripts. *Nucleic Acids Res.* 43 7590–7599. 10.1093/nar/gkv604 26071954 PMC4551911

[B71] Hartung J. S. Roy A. Fu S. Shao J. Schneider W. L. Brlansky R. H. (2015). History and diversity of *Citrus leprosis virus* recorded in herbarium specimens. *Phytopathology* 105 1277–1284. 10.1094/PHYTO-03-15-0064-R 25961338

[B72] Henderson I. R. Zhang X. Lu C. Johnson L. Meyers B. C. Green P. J. (2006). Dissecting *Arabidopsis thaliana* DICER function in small RNA processing, gene silencing and DNA methylation patterning. *Nat. Genet.* 38 721–725. 10.1038/ng1804 16699516

[B73] Hong W. Qian D. Sun R. Jiang L. Wang Y. Wei C. (2015). *OsRDR6* plays role in host defense against double-stranded RNA virus, *Rice Dwarf Phytoreovirus*. *Sci. Rep.* 5:11324. 10.1038/srep11324 26165755 PMC4499934

[B74] Howard S. Qiu W. (2017). Viral small RNAs reveal the genomic variations of three grapevine vein clearing virus quasispecies populations. *Virus Res.* 229 24–27. 10.1016/j.virusres.2016.12.012 28012998

[B75] Hull R. (2014). *Plant Virology.* Cambridge, MA: Academic Press.

[B76] Jackel J. N. Buchmann R. C. Singhal U. Bisaro D. M. (2015). Analysis of geminivirus AL2 and L2 proteins reveals a novel AL2 silencing suppressor activity. *J. Virol.* 89 3176–3187. 10.1128/JVI.02625-14 25552721 PMC4337558

[B77] Jackel J. N. Storer J. M. Coursey T. Bisaro D. M. (2016). *Arabidopsis* RNA polymerases IV and V are required to establish H3K9 methylation, but not cytosine methylation, on geminivirus chromatin. *J*. *Virol.* 90 7529–7540. 10.1128/JVI.00656-16 27279611 PMC4984644

[B78] Jeffrey K. L. Li Y. Ding S. W. (2017). Reply to ‘Questioning antiviral RNAi in mammals’. *Nat. Microbiol.* 2:17053. 10.1038/nmicrobiol.2017.53 28440274 PMC5488271

[B79] Jeske H. (2018). Barcoding of plant viruses with circular single-stranded DNA based on rolling circle amplification. *Viruses* 10:469. 10.3390/v10090469 30200312 PMC6164888

[B80] Jiang L. Qian D. Zheng H. Meng L. Y. Chen J. Le W. J. (2012). RNA-dependent RNA polymerase 6 of rice (*Oryza sativa*) plays role in host defense against negative-strand RNA virus, *Rice stripe virus*. *Virus Res.* 163 512–519. 10.1016/j.virusres.2011.11.016 22142475

[B81] Jimenez J. Carvajal-Yepes M. Leiva A. M. Cruz M. Romero L. E. Bolaños C. A. (2018). Complete genome sequence of *Rice hoja blanca tenuivirus* isolated from a susceptible rice cultivar in Colombia. *Genome Announc.* 6:e01490-17. 10.1128/genomeA.01490-17 29449400 PMC5814491

[B82] Jones S. Baizan-Edge A. MacFarlane S. Torrance L. (2017). Viral diagnostics in plants using next generation sequencing: computational analysis in practice. *Front. Plant Sci.* 8:1770. 10.3389/fpls.2017.01770 29123534 PMC5662881

[B83] Kalischuk M. L. Fusaro A. F. Waterhouse P. M. Pappu H. R. Kawchuk L. M. (2013). Complete genomic sequence of a *Rubus yellow net virus* isolate and detection of genome-wide pararetrovirus-derived small RNAs. *Virus Res.* 178 306–313. 10.1016/j.virusres.2013.09.026 24076299

[B84] Kashif M. Pietilä S. Artola K. Jones R. A. C. Tugume A. K. Mäkinen V. (2012). Detection of viruses in sweetpotato from Honduras and Guatemala augmented by deep-sequencing of small-RNAs. *Plant Dis.* 96 1430–1437. 10.1094/PDIS-03-12-0268-RE 30727310

[B85] Katsarou K. Mavrothalassiti E. Dermauw W. Van Leeuwen T. Kalantidis K. (2016). Combined activity of DCL2 and DCL3 is crucial in the defense against *Potato spindle tuber viroid*. *PLoS Pathog.* 12:e1005936. 10.1371/journal.ppat.1005936 27732664 PMC5061435

[B86] Katsarou K. Rao A. L. Tsagris M. Kalantidis K. (2015). Infectious long non-coding RNAs. *Biochimie* 117 37–47. 10.1016/j.biochi.2015.05.005 25986218

[B87] Kazmi S. A. Yang Z. Hong N. Wang G. Wang Y. (2015). Characterization by small RNA sequencing of taro bacilliform CH virus (TaBCHV), a novel badnavirus. *PLoS One* 10:e0134147. 10.1371/journal.pone.0134147 26207896 PMC4514669

[B88] Kishigami R. Yamagishi N. Ito T. Yoshikawa N. (2014). Detection of apple latent spherical virus in seeds PCR and seedlings from infected apple trees by reverse transcription quantitative sequencing deep: evidence for lack of transmission of the virus to most progeny seedlings. *J. Gen. Plant Pathol.* 80 490–498. 10.1007/s10327-014-0541-3

[B89] Kreuze J. (2014). “siRNA deep sequencing and assembly: piecing together viral infections,” in *Detection and Diagnostics of Plant Pathogens, Plant Pathology in the 21st Century 5*, eds Gullino M. L. Bonants P. J. M. (Dordrecht: Springer Science+Business Media), 21–38.

[B90] Kreuze J. F. Perez A. Untiveros M. Quispe D. Fuentes S. Barker I. (2009). Complete viral genome sequence and discovery of novel viruses by deep sequencing of small RNAs: a generic method for diagnosis, discovery and sequencing of viruses. *Virology* 388 1–7. 10.1016/j.virol.2009.03.024 19394993

[B91] Kutnjak D. Rupar M. Gutierrez-Aguirre I. Curk T. Kreuze J. F. Ravnikar M. (2015). Deep sequencing of virus-derived small interfering RNAs and RNA from viral particles shows highly similar mutational landscapes of a plant virus population. *J. Virol.* 89 4760–4769. 10.1128/JVI.03685-14 25673712 PMC4403455

[B92] Kutnjak D. Silvestre R. Cuellar W. Perez W. Müller G. Ravnikar M. (2014). Complete genome sequences of new divergent potato virus X isolates and discrimination between strains in a mixed infection using small RNAs sequencing approach. *Virus Res.* 191 45–50. 10.1016/j.virusres.2014.07.012 25051147

[B93] Lan Y. Li Y. E Z. Sun F. Du L. Xu Q. (2018). Identification of virus-derived siRNAs and their targets in RBSDV-infected rice by deep sequencing. *J. Basic Microbiol.* 58 227–237. 10.1002/jobm.201700325 29215744

[B94] Lee J. Yang E. C. Graf L. Yang J. H. Qiu H. Zelzion U. (2018). Analysis of the draft genome of the red seaweed *Gracilariopsis chorda* provides insights into genome size evolution in rhodophyta. *Mol. Biol. Evol.* 35 1869–1886. 10.1093/molbev/msy081 29688518

[B95] Leibman D. Prakash S. Wolf D. Zelcer A. Anfoka G. Haviv S. (2015). Immunity to tomato yellow leaf curl virus in transgenic tomato is associated with accumulation of transgene small RNA. *Arch. Virol.* 160 2727–2739. 10.1007/s00705-015-2551-7 26255053

[B96] Leibman D. Wolf D. Saharan V. Zelcer A. Arazi T. Yoel S. (2011). A high level of transgenic viral small RNA is associated with broad potyvirus resistance in cucurbits. *Mol. Plant Microbe Interact.* 24 1220–1238. 10.1094/MPMI-05-11-0128 21899438

[B97] Li R. Gao S. Hernandez A. G. Wechter W. P. Fei Z. Ling K. S. (2012). Deep sequencing of small RNAs in tomato for virus and viroid identification and strain differentiation. *PLoS One* 7:e37127. 10.1371/journal.pone.0037127 22623984 PMC3356388

[B98] Liu W. Duttke S. H. Hetzel J. Groth M. Feng S. Gallego-Bartolome J. (2018). RNA-directed DNA methylation involves co-transcriptional small-RNA-guided slicing of polymerase V transcripts in *Arabidopsis*. *Nat. Plants* 4 181–188. 10.1038/s41477-017-0100-y 29379150 PMC5832601

[B99] Liu Y. El-Kassaby Y. A. (2017). Landscape of fluid sets of hairpin-derived 21-/24-nt-long small RNAs at seed set uncovers special epigenetic features in *Picea glauca*. *Genome Biol. Evol.* 9 82–92. 10.1093/gbe/evw283 28082604 PMC5381586

[B100] Lockhart B. E. Menke J. Dahal G. Olszewski N. E. (2000). Characterization and genomic analysis of tobacco vein clearing virus, a plant pararetrovirus that is transmitted vertically and related to sequences integrated in the host genome. *J. Gen. Virol.* 81 1579–1585. 10.1099/0022-1317-81-6-1579 10811941

[B101] Lózsa R. Csorba T. Lakatos L. Burgyán J. (2008). Inhibition of 3’ modification of small RNAs in virus-infected plants require spatial and temporal co-expression of small RNAs and viral silencing-suppressor proteins. *Nucleic Acids Res.* 36 4099–4107. 10.1093/nar/gkn365 18539609 PMC2475607

[B102] Ma L. Hatlen A. Kelly L. J. Becher H. Wang W. Kovarik A. (2015). Angiosperms are unique among land plant lineages in the occurrence of key genes in the RNA-directed DNA methylation (RdDM) pathway. *Genome Biol. Evol.* 7 2648–2662. 10.1093/gbe/evv171 26338185 PMC4607528

[B103] Maliogka V. I. Minafra A. Saldarelli P. Ruiz-García A. B. Glasa M. Katis N. (2018). Recent advances on detection and characterization of fruit tree viruses using high-throughput sequencing technologies. *Viruses* 10:E436. 10.3390/v10080436 30126105 PMC6116224

[B104] Maliogka V. I. Olmos A. Pappi P. G. Lotos L. Efthimiou K. Grammatikaki G. (2015). A novel grapevine badnavirus is associated with the Roditis leaf discoloration disease. *Virus Res.* 203 47–55. 10.1016/j.virusres.2015.03.003 25791736

[B105] Malpica-López N. Rajeswaran R. Beknazariants D. Seguin J. Golyaev V. Farinelli L. (2018). Revisiting the roles of tobamovirus replicase complex proteins in viral replication and silencing suppression. *Mol. Plant Microbe Interact.* 31 125–144. 10.1094/MPMI-07-17-0164-R 29140168

[B106] Mandal B. (2010). Advances in small isometric multicomponent ssDNA viruses infecting plants. *Indian J. Virol.* 21 18–30. 10.1007/s13337-010-0010-3 23637475 PMC3550773

[B107] Marcon H. S. Costa-Silva J. Lorenzetti A. P. R. Marino C. L. Domingues D. S. (2017). Genome-wide analysis of EgEVE_1, a transcriptionally active endogenous viral element associated to small RNAs in Eucalyptus genomes. *Genet. Mol. Biol.* 40 217–225. 10.1590/1678-4685-GMB-2016-0086 28235127 PMC5452135

[B108] Massart S. Chiumenti M. De Jonghe K. Glover R. Haegeman A. Koloniuk I. (2018). Virus detection by high-throughput sequencing of small RNAs: large scale performance testing of sequence analysis strategies. *Phytopathology* 10.1094/PHYTO-02-18-0067-R [Epub ahead of print]. 30070618

[B109] Masuta Y. Nozawa K. Takagi H. Yaegashi H. Tanaka K. Ito T. (2017). Inducible transposition of a heat-activated retrotransposon in tissue culture. *Plant Cell Physiol.* 58 375–384. 10.1093/pcp/pcw202 28013279

[B110] Matzke M. A. Kanno T. Matzke A. J. (2015). Evolution of a complex epigenetic pathway in flowering plants. *Annu. Rev. Plant Biol.* 66 243–267. 10.1146/annurev-arplant-043014-114633 25494460

[B111] Mbanzibwa D. R. Tugume A. K. Chiunga E. Mark D. Tairo F. D. (2014). Small RNA deep sequencing-based detection and further evidence of DNA viruses infecting sweetpotato plants in Tanzania. *Ann. Appl. Biol.* 165 329–339. 10.1111/aab.12136

[B112] Mette M. F. Kanno T. Aufsatz W. Jakowitsch J. van der Winden J. Matzke M. A. (2002). Endogenous viral sequences and their potential contribution to heritable virus resistance in plants. *EMBO J.* 21 461–469. 10.1093/emboj/21.3.461 11823438 PMC125834

[B113] Minoia S. Carbonell A. Di Serio F. Gisel A. Carrington J. C. Navarro B. (2014). Specific argonautes selectively bind small RNAs derived from *Potato spindle tuber viroid* and attenuate viroid accumulation *in vivo*. *J. Virol.* 88 11933–11945. 10.1128/JVI.01404-14 25100851 PMC4178711

[B114] Molnár A. Csorba T. Lakatos L. Várallyay E. Lacomme C. Burgyán J. (2005). Plant virus-derived small interfering RNAs originate predominantly from highly structured single-stranded viral RNAs. *J. Virol.* 79 7812–7818. 10.1128/JVI.79.12.7812-7818.2005 15919934 PMC1143663

[B115] Mongelli V. Saleh M. C. (2016). Bugs are not to be silenced: small RNA pathways and antiviral responses in insects. *Annu. Rev. Virol.* 3 573–589. 10.1146/annurev-virology-110615-042447 27741406

[B116] Morelli M. Giampetruzzi A. Laghezza L. Catalano L. Savino V. N. Saldarelli P. (2017). Identification and characterization of an isolate of apple green crinkle associated virus involved in a severe disease of quince (*Cydonia oblonga*, Mill.). *Arch. Virol.* 162 299–306. 10.1007/s00705-016-3074-6 27709400

[B117] Murad L. Bielawski J. P. Matyasek R. Kovarík A. Nichols R. A. Leitch A. R. (2004). The origin and evolution of geminivirus-related DNA sequences in *Nicotiana*. *Heredity* 92 352–358. 10.1038/sj.hdy.6800431 14985783

[B118] Mwaipopo B. Msolla N. S. Njau P. Mark D. Mbanzibwa D. R. (2018). Comprehensive surveys of *Bean common mosaic virus* and *Bean common mosaic necrosis virus* and molecular evidence for occurrence of other *Phaseolus vulgaris* viruses in Tanzania. *Plant Dis.* 102 2361–2370. 10.1094/PDIS-01-18-0198-RE 30252625 PMC7779967

[B119] Navarro B. Gisel A. Rodio M. E. Delgado S. Flores R. Di Serio F. (2012). Small RNAs containing the pathogenic determinant of a chloroplast-replicating viroid guide the degradation of a host mRNA as predicted by RNA silencing. *Plant J.* 70 991–1003. 10.1111/j.1365-313X.2012.04940.x 22332758

[B120] Nayak A. Tassetto M. Kunitomi M. Andino R. (2013). RNA interference-mediated intrinsic antiviral immunity in invertebrates. *Curr. Top. Microbiol. Immunol.* 371 183–200. 10.1007/978-3-642-37765-5_7 23686236

[B121] Ndowora T. Dahal G. LaFleur D. Harper G. Hull R. Olszewski N. E. (1999). Evidence that badnavirus infection in *Musa* can originate from integrated pararetroviral sequences. *Virology* 255 214–220. 10.1006/viro.1998.9582 10069946

[B122] Noreen F. Akbergenov R. Hohn T. Richert-Pöggeler K. R. (2007). Distinct expression of endogenous *Petunia vein clearing virus* and the DNA transposon *dTph1* in two *Petunia hybrida* lines is correlated with differences in histone modification and siRNA production. *Plant J.* 50 219–229. 10.1111/j.1365-313X.2007.03040.x 17444906

[B123] Pantaleo V. Saldarelli P. Miozzi L. Giampetruzzi A. Gisel A. Moxon S. (2010). Deep sequencing analysis of viral short RNAs from an infected Pinot Noir grapevine. *Virology* 408 49–56. 10.1016/j.virol.2010.09.001 20875658

[B124] Parameswaran P. Sklan E. Wilkins C. Burgon T. Samuel M. A. Lu R. (2010). Six RNA viruses and forty-one hosts: viral small RNAs and modulation of small RNA repertoires in vertebrate and invertebrate systems. *PLoS Pathog.* 6:e1000764. 10.1371/journal.ppat.1000764 20169186 PMC2820531

[B125] Patil B. L. Arora D. J. (2018). Comparative characterization of small RNAs derived from an emaravirus and a geminivirus infecting pigeonpea. *J. Plant Biochem. Biotechnol.* 27 382–392. 10.1007/s13562-018-0447-9

[B126] Pecman A. Kutnjak D. Gutiérrez-Aguirre I. Adams I. Fox A. Boonham N. (2017). Next generation sequencing for detection and discovery of plant viruses and viroids: comparison of two approaches. *Front. Microbiol.* 8:1998. 10.3389/fmicb.2017.01998 29081770 PMC5645528

[B127] Pérez-Cañamás M. Blanco-Pérez M. Forment J. Hernández C. (2017). *Nicotiana benthamiana* plants asymptomatically infected by *Pelargonium line pattern virus* show unusually high accumulation of viral small RNAs that is neither associated with DCL induction nor RDR6 activity. *Virology* 501 136–146. 10.1016/j.virol.2016.11.018 27915129

[B128] Pooggin M. M. (2013). How can plant DNA viruses evade siRNA-directed DNA methylation and silencing? *Int. J. Mol. Sci.* 14 15233–15259. 10.3390/ijms140815233 23887650 PMC3759858

[B129] Pooggin M. M. (2016). “Role of small RNAs in virus host interaction,” in *Plant-Virus Interactions - Molecular Biology, Intra- and Intercellular Transport*, ed. Klejnow T. (Berlin: Springer), 161–189.

[B130] Pooggin M. M. (2017). RNAi-mediated resistance to viruses: a critical assessment of methodologies. *Curr. Opin. Virol.* 26 28–35. 10.1016/j.coviro.2017.07.010 28753441

[B131] Pooggin M. M. Ryabova L. A. (2018). Ribosome shunting, polycistronic translation, and evasion of antiviral defenses in plant pararetroviruses and beyond. *Front. Microbiol.* 9:644. 10.3389/fmicb.2018.00644 29692761 PMC5902531

[B132] Qiao W. Zarzyñska-Nowak A. Nerva L. Kuo Y. W. Falk B. W. (2018). Accumulation of 24 nucleotide transgene-derived siRNAs is associated with Crinivirus immunity in transgenic plants. *Mol. Plant Pathol.* 19 2236–2247. 10.1111/mpp.12695 29704454 PMC6638120

[B133] Qin C. Li B. Fan Y. Zhang X. Yu Z. Ryabov E. (2017). Roles of dicer-like proteins 2 and 4 in intra- and intercellular antiviral silencing. *Plant Physiol.* 174 1067–1081. 10.1104/pp.17.00475 28455401 PMC5462052

[B134] Raja P. Jackel J. N. Li S. Heard I. M. Bisaro D. M. (2014). *Arabidopsis* double-stranded RNA binding protein DRB3 participates in methylation-mediated defense against geminiviruses. *J. Virol.* 88 2611–2622. 10.1128/JVI.02305-13 24352449 PMC3958096

[B135] Rajeswaran R. Golyaev V. Seguin J. Zvereva A. S. Farinelli L. Pooggin M. M. (2014a). Interactions of Rice tungro bacilliform pararetrovirus and its protein P4 with plant RNA-silencing machinery. *Mol. Plant Microbe Interact.* 27 1370–1378. 10.1094/MPMI-07-14-0201-R 25122481

[B136] Rajeswaran R. Seguin J. Chabannes M. Duroy P. O. Laboureau N. Farinelli L. (2014b). Evasion of short interfering RNA-directed antiviral silencing in *Musa acuminata* persistently infected with six distinct banana streak pararetroviruses. *J. Virol.* 88 11516–11528. 10.1128/JVI.01496-14 25056897 PMC4178793

[B137] Ramesh S. V. Sahu P. P. Prasad M. Praveen S. Pappu H. R. (2017). Geminiviruses and plant hosts: a closer examination of the molecular arms race. *Viruses* 9:E256. 10.3390/v9090256 28914771 PMC5618022

[B138] Rao A. L. Kalantidis K. (2015). Virus-associated small satellite RNAs and viroids display similarities in their replication strategies. *Virology* 479–480, 627–636. 10.1016/j.virol.2015.02.018 25731957

[B139] Richert-Pöggeler K. R. Noreen F. Schwarzacher T. Harper G. Hohn T. (2003). Induction of infectious petunia vein clearing (pararetro) virus from endogenous provirus in petunia. *EMBO J.* 22 4836–4845. 10.1093/emboj/cdg443 12970195 PMC212712

[B140] Rogers K. Chen X. (2013). Biogenesis, turnover, and mode of action of plant microRNAs. *Plant Cell* 25 2383–2399. 10.1105/tpc.113.113159 23881412 PMC3753372

[B141] Roossinck M. J. (2017). Deep sequencing for discovery and evolutionary analysis of plant viruses. *Virus Res.* 239 82–86. 10.1016/j.virusres.2016.11.019 27876625

[B142] Roossinck M. J. Martin D. P. Roumagnac P. (2015). Plant virus metagenomics: advances in virus discovery. *Phytopathology* 105 716–727. 10.1094/PHYTO-12-14-0356-RVW 26056847

[B143] Roy A. Choudhary N. Guillermo L. M. Shao J. Govindarajulu A. Achor D. (2013a). A novel virus of the genus *Cilevirus* causing symptoms similar to citrus leprosis. *Pytopathology* 103 488–500. 10.1094/PHYTO-07-12-0177-R 23268581

[B144] Roy A. Shao J. Hartung J. S. Schneider W. Brlansky R. H. (2013b). A case study on discovery of novel *Citrus Leprosis Virus* cytoplasmic type 2 utilizing small RNA libraries by next generation sequencing and bioinformatic analyses. *J. Data Mining Genomics Proteomics* 4:129 10.4172/2153-0602.1000129

[B145] Roy A. Stone A. Otero-Colina G. Wei G. Choudhary N. Achor D. (2013c). Genome assembly of *citrus leprosis virus* nuclear type reveals a close association with orchid fleck virus. *Genome Announc.* 1:e00519-13. 10.1128/genomeA.00519-13 23887919 PMC3735072

[B146] Ruiz-Ruiz S. Navarro B. Gisel A. Peña L. Navarro L. Moreno P. (2011). Citrus tristeza virus infection induces the accumulation of viral small RNAs (21-24-nt) mapping preferentially at the 3′-terminal region of the genomic RNA and affects the host small RNA profile. *Plant Mol. Biol.* 75 607–619. 10.1007/s11103-011-9754-4 21327514

[B147] Santala J. Valkonen J. P. T. (2018). Sensitivity of small RNA-based detection of plant viruses. *Front. Microbiol.* 9:939. 10.3389/fmicb.2018.00939 29867848 PMC5960716

[B148] Sattar S. Thompson G. A. (2016). Small RNA regulators of plant-hemipteran interactions: micromanagers with versatile roles. *Front. Plant Sci.* 7:1241. 10.3389/fpls.2016.01241 27625654 PMC5003895

[B149] Schuck J. Gursinsky T. Pantaleo V. Burgyán J. Behrens S. E. (2013). AGO/RISC-mediated antiviral RNA silencing in a plant in vitro system. *Nucleic Acids Res.* 41 5090–5103. 10.1093/nar/gkt193 23535144 PMC3643602

[B150] Seguin J. Otten P. Baerlocher L. Farinelli L. Pooggin M. M. (2016). MISIS-2: a bioinformatics tool for in-depth analysis of small RNAs and representation of consensus master genome in viral quasispecies. *J. Virol. Methods* 233 37–40. 10.1016/j.jviromet.2016.03.005 26994965

[B151] Seguin J. Rajeswaran R. Malpica-López N. Martin R. R. Kasschau K. Dolja V. V. (2014). De novo reconstruction of consensus master genomes of plant RNA and DNA viruses from siRNAs. *PLoS One* 9:e88513. 10.1371/journal.pone.0088513 24523907 PMC3921208

[B152] Shamandi N. Zytnicki M. Charbonnel C. Elvira-Matelot E. Bochnakian A. Comella P. (2015). Plants encode a general siRNA suppressor that is induced and suppressed by viruses. *PLoS Biol.* 13:e1002326. 10.1371/journal.pbio.1002326 26696443 PMC4687873

[B153] Shen W. X. Au P. C. Shi B. J. Smith N. A. Dennis E. S. Guo H. S. (2015). Satellite RNAs interfere with the function of viral RNA silencing suppressors. *Front. Plant Sci.* 6:281. 10.3389/fpls.2015.00281 25964791 PMC4408847

[B154] Shimura H. Pantaleo V. Ishihara T. Myojo N. Inaba J. Sueda K. (2011). A viral satellite RNA induces yellow symptoms on tobacco by targeting a gene involved in chlorophyll biosynthesis using the RNA silencing machinery. *PLoS Pathog.* 7:e1002021. 10.1371/journal.ppat.1002021 21573143 PMC3088725

[B155] Shivaprasad P. V. Rajeswaran R. Blevins T. Schoelz J. Meins F. Jr. Hohn T. (2008). The CaMV transactivator/viroplasmin interferes with RDR6-dependent *trans*-acting and secondary siRNA pathways in *Arabidopsis*. *Nucleic Acids Res.* 36 5896–5909. 10.1093/nar/gkn590 18801846 PMC2566869

[B156] Smith O. Clapham A. Rose P. Liu Y. Wang J. Allaby R. G. (2014). A complete ancient RNA genome: identification, reconstruction and evolutionary history of archaeological Barley Stripe Mosaic Virus. *Sci. Rep.* 4:4003. 10.1038/srep04003 24499968 PMC3915304

[B157] Staginnus C. Gregor W. Mette M. F. Teo C. H. Borroto-Fernández E. G. Machado M. L. (2007). Endogenous pararetroviral sequences in tomato (*Solanum lycopersicum*) and related species. *BMC Plant Biol.* 7:24. 10.1186/1471-2229-7-24 17517142 PMC1899175

[B158] Sun F. Guo W. Du J. Ni Z. Sun Q. Yao Y. (2013). Widespread, abundant, and diverse TE-associated siRNAs in developing wheat grain. *Gene* 522 1–7. 10.1016/j.gene.2013.03.101 23562726

[B159] Šurbanovski N. Brilli M. Moser M. Si-Ammour A. (2016). A highly specific microRNA-mediated mechanism silences LTR retrotransposons of strawberry. *Plant J.* 85 70–82. 10.1111/tpj.13090 26611654

[B160] Szittya G. Moxon S. Pantaleo V. Toth G. Rusholme Pilcher R. L. Moulton V. (2010). Structural and functional analysis of viral siRNAs. *PLoS Pathog.* 6:e1000838. 10.1371/journal.ppat.1000838 20368973 PMC2848561

[B161] tenOever B. R. (2016). The evolution of antiviral defense systems. *Cell Host Microbe* 19 142–149. 10.1016/j.chom.2016.01.006 26867173

[B162] tenOever B. R. (2017). Questioning antiviral RNAi in mammals. *Nat. Microbiol.* 2:17052. 10.1038/nmicrobiol.2017.52 28440277

[B163] Torchetti E. M. Pegoraro M. Navarro B. Catoni M. Di Serio F. Noris E. (2016). A nuclear-replicating viroid antagonizes infectivity and accumulation of a geminivirus by upregulating methylation-related genes and inducing hypermethylation of viral DNA. *Sci. Rep.* 6:35101. 10.1038/srep35101 27739453 PMC5064398

[B164] Tsushima D. Adkar-Purushothama C. R. Taneda A. Sano T. (2015). Changes in relative expression levels of viroid-specific small RNAs and microRNAs in tomato plants infected with severe and mild symptom-inducing isolates of *Potato spindle tuber viroid*. *J. Gen. Plant Pathol.* 81 49–62. 10.1007/s10327-014-0566-7

[B165] Turco S. Golyaev V. Seguin J. Gilli C. Farinelli L. Boller T. (2018). Small RNA-omics for virome reconstruction and antiviral defense characterization in mixed infections of cultivated *Solanum* plants. *Mol. Plant Microbe Interact.* 31 707–723. 10.1094/MPMI-12-17-0301-R 29424662

[B166] Ujino-Ihara T. Ueno S. Uchiyama K. Futamura N. (2018). Comprehensive analysis of small RNAs expressed in developing male strobili of *Cryptomeria japonica*. *PLoS One* 13:e0193665. 10.1371/journal.pone.0193665 29529051 PMC5846777

[B167] Untiveros M. Olspert A. Artola K. Firth A. E. Kreuz J. F. Valkonen J. P. (2016). A novel sweet potato potyvirus open reading frame (ORF) is expressed via polymerase slippage and suppresses RNA silencing. *Mol. Plant Pathol.* 17 1111–1123. 10.1111/mpp.12366 26757490 PMC4979677

[B168] Vainio E. J. Jurvansuu J. Streng J. Rajamäki M. L. Hantula J. Valkonen J. P. (2015). Diagnosis and discovery of fungal viruses using deep sequencing of small RNAs. *J. Gen. Virol.* 96 714–725. 10.1099/jgv.0.000003 25480928

[B169] Velasco L. Arjona-Girona I. Ariza-Fernández M. T. Cretazzo E. López-Herrera C. (2018). A novel hypovirus species from xylariaceae fungi infecting avocado. *Front. Microbiol.* 9:778. 10.3389/fmicb.2018.00778 29867781 PMC5952064

[B170] Verdin E. Wipf-Scheibel C. Gognalons P. Aller F. Jacquemond M. Tepfer M. (2017). Sequencing viral siRNAs to identify previously undescribed viruses and viroids in a panel of ornamental plant samples structured as a matrix of pools. *Virus Res.* 241 19–28. 10.1016/j.virusres.2017.05.019 28576697

[B171] Wang F. Sun Y. Ruan J. Chen R. Chen X. Chen C. (2016). Using small RNA deep sequencing data to detect human viruses. *Biomed Res. Int.* 2016:2596782. 10.1155/2016/2596782 27066498 PMC4811048

[B172] Waldron F. M. Stone G. N. Obbard D. J. (2018). Metagenomic sequencing suggests a diversity of RNA interference-like responses to viruses across multicellular eukaryotes. *PLoS Genet.* 14:e1007533. 10.1371/journal.pgen.1007533 30059538 PMC6085071

[B173] Wang J. Tang Y. Yang Y. Ma N. Ling X. Kan J. (2016). Cotton leaf curl multan virus-derived viral small RNAs can target cotton genes to promote viral infection. *Front. Plant Sci.* 7:1162. 10.3389/fpls.2016.01162 27540385 PMC4972823

[B174] Wang T. Deng Z. Zhang X. Wang H. Wang Y. Liu X. (2018). Tomato *DCL2b* is required for the biosynthesis of 22-nt small RNAs, the resulting secondary siRNAs, and the host defense against ToMV. *Hortic Res.* 5:62. 10.1038/s41438-018-0073-7 30181890 PMC6119189

[B175] Wang X. B. Jovel J. Udomporn P. Wang Y. Wu Q. Li W. X. (2011). The 21-nucleotide, but not 22-nucleotide, viral secondary small interfering RNAs direct potent antiviral defense by two cooperative argonautes in *Arabidopsis thaliana*. *Plant Cell* 23 1625–1638. 10.1105/tpc.110.082305 21467580 PMC3101545

[B176] Wang X. B. Wu Q. Ito T. Cillo F. Li W. X. Chen X. (2010). RNAi-mediated viral immunity requires amplification of virus-derived siRNAs in *Arabidopsis thaliana*. *Proc. Natl. Acad. Sci. U.S.A.* 107 484–489. 10.1073/pnas.0904086107 19966292 PMC2806737

[B177] Wang Y. Cheng X. Wu X. Wang A. Wu X. (2014). Characterization of complete genome and small RNA profile of pagoda yellow mosaic associated virus, a novel badnavirus in China. *Virus Res.* 188 103–108. 10.1016/j.virusres.2014.04.006 24751798

[B178] Wang Y. Liang W. Tang T. (2018). Constant conflict between Gypsy LTR retrotransposons and CHH methylation within a stress-adapted mangrove genome. *New Phytol.* 220 922–935. 10.1111/nph.15209 29762876

[B179] Wassenegger M. Heimes S. Riedel L. Sänger H. L. (1994). RNA-directed de novo methylation of genomic sequences in plants. *Cell* 76 567–576. 10.1016/0092-8674(94)90119-8 8313476

[B180] Wu J. Yang Z. Wang Y. Zheng L. Ye R. Ji Y. (2015). Viral-inducible Argonaute18 confers broad-spectrum virus resistance in rice by sequestering a host microRNA. *eLife* 4:e05733. 10.7554/eLife.05733 25688565 PMC4358150

[B181] Wu Q. Ding S. W. Zhang Y. Zhu S. (2015). Identification of viruses and viroids by next-generation sequencing and homology-dependent and homology-independent algorithms. *Annu. Rev. Phytopathol.* 53 425–444. 10.1146/annurev-phyto-080614-120030 26047558

[B182] Wu Q. Luo Y. Lu R. Lau N. Lai E. C. Li W. X. (2010). Virus discovery by deep sequencing and assembly of virus-derived small silencing RNAs. *Proc. Natl. Acad. Sci. U.S.A.* 107 1606–1611. 10.1073/pnas.0911353107 20080648 PMC2824396

[B183] Wu Q. Wang Y. Cao M. Pantaleo V. Burgyan J. Li W. X. (2012). Homology-independent discovery of replicating pathogenic circular RNAs by deep sequencing and a new computational algorithm. *Proc. Natl. Acad. Sci. U.S.A.* 109 3938–3943. 10.1073/pnas.1117815109 22345560 PMC3309787

[B184] Xie Z. Johansen L. K. Gustafson A. M. Kasschau K. D. Lellis A. D. Zilberman D. (2004). Genetic and functional diversification of small RNA pathways in plants. *PLoS Biol.* 2:E104. 10.1371/journal.pbio.0020104 15024409 PMC350667

[B185] Xu C. Sun X. Taylor A. Jiao C. Xu Y. Cai X. (2017). Diversity, distribution, and evolution of tomato viruses in china uncovered by small RNA sequencing. *J. Virol.* 91:e00173-17. 10.1128/JVI.00173-17 28331089 PMC5432854

[B186] Xu D. Zhou G. (2017). Characteristics of siRNAs derived from *Southern rice black-streaked dwarf virus* in infected rice and their potential role in host gene regulation. *Virol. J.* 14:27. 10.1186/s12985-017-0699-3 28183327 PMC5301327

[B187] Xu J. Liu D. Zhang Y. Wang Y. Han C. Li D. (2016). Improved pathogenicity of a beet black scorch virus variant by low temperature and co-infection with its satellite RNA. *Front. Microbiol.* 7:1771. 10.3389/fmicb.2016.01771 27867378 PMC5095503

[B188] Xu Y. Huang L. Fu S. Wu J. Zhou X. (2012). Population diversity of *Rice stripe virus*-derived siRNAs in three different hosts and RNAi-based antiviral immunity in *Laodelphgax striatellus*. *PLoS One* 7:e46238. 10.1371/journal.pone.0046238 23029445 PMC3460854

[B189] Yaegashi H. Shimizu T. Ito T. Kanematsu S. (2016). Differential inductions of RNA silencing among encapsidated double-stranded RNA mycoviruses in the white root rot fungus *Rosellinia necatrix*. *J. Virol.* 90 5677–5692. 10.1128/JVI.02951-15 27030271 PMC4886788

[B190] Yan F. Zhang H. Adams M. J. Yang J. Peng J. Antoniw J. F. (2010). Characterization of siRNAs derived from *Rice stripe virus* in infected rice plants by deep sequencing. *Arch. Virol.* 155 935–940. 10.1007/s00705-010-0670-8 20396917

[B191] Yan T. Zhu J. R. Di D. Gao Q. Zhang Y. Zhang A. (2015). Characterization of the complete genome of Barley yellow striate mosaic virus reveals a nested gene encoding a small hydrophobic protein. *Virology* 478 112–122. 10.1016/j.virol.2014.12.042 25666524

[B192] Yang X. Huang J. Liu C. Chen B. Zhang T. Zhou G. (2017). Rice stripe mosaic virus, a novel cytorhabdovirus infecting rice via leafhopper transmission. *Front. Microbiol.* 7:2140. 10.3389/fmicb.2016.02140 28101087 PMC5210121

[B193] Yang X. Wang Y. Guo W. Xie Y. Xie Q. Fan L. (2011). Characterization of small interfering RNAs derived from the geminivirus/betasatellite complex using deep sequencing. *PLoS One* 6:e16928. 10.1371/journal.pone.0016928 21347388 PMC3036729

[B194] You C. Cui J. Wang H. Qi X. Kuo L. Y. Ma H. (2017). Conservation and divergence of small RNA pathways and microRNAs in land plants. *Genome Biol.* 18:158. 10.1186/s13059-017-1291-2 28835265 PMC5569507

[B195] Zahid K. Zhao J. H. Smith N. A. Schumann U. Fang Y. Y. Dennis E. S. (2015). Nicotiana small RNA sequences support a host genome origin of cucumber mosaic virus satellite RNA. *PLoS Genet.* 11:e1004906. 10.1371/journal.pgen.1004906 25568943 PMC4287446

[B196] Zarreen F. Kumar G. Johnson A. M. A. Dasgupta I. (2018). Small RNA-based interactions between rice and the viruses which cause the tungro disease. *Virology* 523 64–73. 10.1016/j.virol.2018.07.022 30081310

[B197] Zhang Y. Singh K. Kaur R. Qiu W. (2011). Association of a novel DNA virus with the grapevine vein-clearing and vine decline syndrome. *Phytopathology* 101 1081–1090. 10.1094/PHYTO-02-11-0034 21554183

[B198] Zhang Z. Qi S. Tang N. Zhang X. Chen S. Zhu P. (2014). Discovery of replicating circular RNAs by RNA-seq and computational algorithms. *PLoS Pathog.* 10:e1004553. 10.1371/journal.ppat.1004553 25503469 PMC4263765

[B199] Zhao D. Song G. Q. (2014a). High-throughput sequencing as an effective approach in profiling small RNAs derived from a hairpin RNA expression vector in woody plants. *Plant Sci.* 228 39–47. 10.1016/j.plantsci.2014.02.013 25438784

[B200] Zhao D. Song G. Q. (2014b). Rootstock-to-scion transfer of transgene-derived small interfering RNAs and their effect on virus resistance in nontransgenic sweet cherry. *Plant Biotechnol. J.* 12 1319–1328. 10.1111/pbi.12243 25132092

[B201] Zheng Y. Gao S. Padmanabhan C. Li R. Galvez M. Gutierrez D. (2017a). VirusDetect: an automated pipeline for efficient virus discovery using deep sequencing of small RNAs. *Virology* 500 130–138. 10.1016/j.virol.2016.10.017 27825033

[B202] Zheng Y. Navarro B. Wang G. Wang Y. Yang Z. Xu W. (2017b). Actinidia chlorotic ringspot-associated virus: a novel emaravirus infecting kiwifruit plants. *Mol. Plant Pathol.* 18 569–581. 10.1111/mpp.12421 27125218 PMC6638214

[B203] Zheng Y. Wang Y. Ding B. Fei Z. (2017c). Comprehensive transcriptome analyses reveal that potato spindle tuber viroid triggers genome-wide changes in alternative splicing, inducible trans-acting activity of phased secondary small interfering RNAs, and immune responses. *J. Virol.* 91:e00247-17. 10.1128/JVI.00247-17 28331096 PMC5432855

[B204] Zheng Y. Z. Wang G. P. Hong N. Zhou J. F. Yang Z. K. Hong N. (2014). First report of *Actinidia virus* A and *Actinidia virus* B on Kiwifruit in China. *Plant Dis.* 98 1590 10.1094/PDIS-04-14-0420-PDN 30699799

