# Supplementary material for: Small RNA-Omics for Plant Virus Identification, Virome Reconstruction, and Antiviral Defense Characterization
Source: Front Microbiol. 2018 Nov 20;9:2779. doi: 10.3389/fmicb.2018.02779 (PMC6256188; doi:10.3389/fmicb.2018.02779)
Supplement: Supplementary file 1 [file Table_1.DOCX]

**Suppementary Table S1. Viruses and their satellites identified in naturally- or experimentally-infected plant hosts and fully or partially reconstructed by small RNA sequencing and assembly**

***** in most cases, 2 or 3 major size-classes of small interfering (si)RNAs (21-, 22- and 24-nucleotide, nt) are indicated in the order of their relative abundance (note that RNA viruses do not normally spawn 24-nt siRNAs); (+) or (-) strand bias of siRNA production is indicated only if it is strong, otherwise “both strands” are indicated.

** if more than one reference, first appear those reporting more detailed information on the viral siRNA profile (if any detail is reported).

| **Family**/*Genus* | *Virus species* | Host plant | Viral siRNA profile* | Reference** |
| --- | --- | --- | --- | --- |
| **Alphaflexiviridae**  (+)ssRNA |  |  |  |  |
| *Mandarivirus* | *Citrus yellow vein clearing virus* | Lemon | 21nt,22nt; both strands | Loconsole et al. 2012a; Cao et al. 2017b |
| *Potexvirus* | *Alstroemeria virus X* | Woolly burdock | 21nt,22nt | Bi et al. 2012b |
| *Potexvirus* | *Bamboo mosaic virus* | *A.thaliana* | 21nt,22nt; both strands | Lin et al. 2010 |
| *Potexvirus* | *Bamboo mosaic virus* | Bamboo | 21nt,22nt; both strands | Lin et al. 2017 |
| *Potexvirus* | *Bamboo mosaic virus* | *N. benthamiana* | 22nt,21nt; both strands | Lin et al. 2010 |
| *Potexvirus* | *Cassava new alphaflexivirus* | Cassava |  | Carvajal-Yepes et al. 2014 |
| *Potexvirus* | *Euonymus yellow vein associated virus* | *Euonymus bungeanus* |  | Yang et al. 2018a |
| *Potexvirus* | *Hosta virus X* | *Hosta* sp. |  | Shchetynina et al. 2017 |
| *Potexvirus* | *Pepino mosaic virus* | Tomato | 21nt,22nt; both strands | Li et al. 2012; Turco et al. 2018; Pecman et al. 2017 |
| *Potexvirus* | *Potato aucuba mosaic virus* | Potato |  | Wu et al. 2018b |
| *Potexvirus* | *Potato virus X* | *N. benthamiana* | 21nt,22nt; both strands | Donaire et al. 2009; Del Toro et al. 2017 |
| *Potexvirus* | *Potato virus X* | Potato | 21nt,22nt; both strands | Turco et al. 2018; Kutnjak et al. 2014; Zheng et al. 2017a; Massart et al. 2018 |
| *Potexvirus* | *Strawberry mild yellow edge virus* | Strawberry |  | Ding et al. 2016 |
| *Potexvirus* | *Tulip virus X* | Cleome |  | Verdin et al. 2017 |
| *Potexvirus* |  | Japanese tree lilac |  | Han et al. 2018 |
| *Potexvirus*  satellite | *Bamboo mosaic virus* satellite RNA | *A. thaliana* | 21nt,22nt; both strands | Lin et al. 2010 |
| *Potexvirus*  satellite | *Bamboo mosaic virus* satellite RNA | Bamboo | 21nt,22nt; (+) strand bias | Lin et al. 2017 |
| *Potexvirus*  satellite | *Bamboo mosaic virus* satellite RNA | *N. benthamiana* | 21nt,22nt; both strands | Lin et al. 2010 |
| **Amalgaviridae**  dsRNA |  |  |  |  |
| *Amalgavirus* | *Southern tomato virus* | Tomato | 21nt,22nt; both strands | Turco et al. 2018; Xu et al. 2017; Padmanabhan et al. 2015b; Pecman et al. 2017; Alcalá-Briseño et al. 2017; Che et al. 2018 |
| *Amalgavirus* | *Watermelon amalgavirus 1* | Watermelon |  | Zaagueri et al. 2017 |
| *Amalgavirus* |  | Anemone |  | Verdin et al. 2017 |
| **Aspiviridae**  (-)ssRNA |  |  |  |  |
| *Ophiovirus* | *Miraﬁori lettuce virus* | Lettuce |  | Kreuze 2014 |
| **Benyviridae**  (+)ssRNA |  |  |  |  |
| *Benyvirus* | *Beet necrotic yellow vein virus* | *Beta macrocarpa* |  | Liu et al. 2017 |
| **Betaflexiviridae**  (+)ssRNA |  |  |  |  |
| *Capillovirus* | *Apple stem grooving virus* | Apple | 21nt,22nt; both strands | Visser et al. 2014; Zhang et al. 2014a; Liang et al. 2015; Massart et al. 2018 |
| *Capillovirus* | *Apple stem grooving virus* | Crab apple |  | Li et al. 2017b |
| *Capillovirus* | *Apple stem grooving virus* | Kiwifruit  *A. chinensis* |  | Wang et al. 2018d |
| *Capillovirus* | *Apple stem grooving virus* | Pear | 21nt,22nt; both strands | Liu et al. 2016b |
| *Capillovirus* | *Apple stem grooving virus* | Rose |  | He et al. 2015 |
| *Capillovirus* | *Cherry virus A* | Apricot | 21nt,22nt; both strands | Baráth et al. 2018 |
| *Capillovirus* | *Cherry virus A* | Sweet cherry | 21nt,22nt; both strands | Wang et al. 2016b,d; Turco, Renyard, Pooggin in prep; Ruiz-Garcia et al. 2016; Wang et al. 2016e; Zamorano et al. 2017 |
| *Carlavirus* | *Cowpea mild mottle virus* | Common bean |  | Nordenstedt et al. 2017; Chiquito-Almanza et al. 2018; Mwaipopo et al. 2018 |
| *Carlavirus* | *Hop latent virus* | Hop |  | Jakse et al. 2015 |
| *Carlavirus* | *Hop mosaic virus* | Hop |  | Jakse et al. 2015 |
| *Carlavirus* | *Ligustrum virus A* | Japanese tree lilac |  | Han et al. 2018 |
| *Carlavirus* | *Lily symptomless virus* | Lily |  | Li et al. 2018 |
| *Carlavirus* | *Potato virus H* | Potato |  | Wu et al. 2018b |
| *Carlavirus* | *Potato virus H* | Tomato | 21nt,22nt; (+) strand bias | Xu et al. 2017 |
| *Carlavirus* | *Potato virus S* | Potato |  | Nie et al. 2015; Zheng et al. 2017a |
| *Carlavirus* | *Potato virus S* | Tomato | 21nt,22nt; (+) strand bias | Xu et al. 2017 |
| *Carlavirus* | *Sweet potato C6 virus* | Sweet potato |  | De Souza et al. 2013 |
| *Carlavirus* | *Sweet potato chlorotic fleck virus* | Sweet potato |  | Mbanzibwa et al. 2014 |
| *Citrivirus* | *Citrus leaf blotch virus* | Lemon |  | Cao et al. 2017b |
| *Citrivirus* | *Citrus leaf blotch virus* | Sweet cherry |  | Wang et al. 2016e |
| *Foveavirus* | *Apple green crinkle associated virus* | Quince | both strands | Morelli et al. 2017 |
| *Foveavirus* | *Apple stem pitting virus* | Apple |  | Zhang et al. 2014a; Liang et al. 2015 |
| *Foveavirus* | *Grapevine rupestris stem-pitting associated virus* | Grapevine | 21nt,22nt; (+) strand bias | Giampetruzzi et al. 2012; Pantaleo et al. 2010; Miozzi et al. 2013a; Saldarelli et al. 2015; Eichmeier et al. 2016; Chiumenti et al. 2016b; Reynard et al. 2016; Barrero et al. 2017; Cretazzo et al. 2017; Czotter et al. 2018 |
| *Tepovirus* | *Potato virus T* | Potato |  | Kreuze 2014; Zheng et al. 2017a |
| *Trichovirus* | *Apple chlorotic leaf spot virus* | Apple |  | Zhang et al. 2014a; Liang et al. 2015 |
| *Trichovirus* | *Apple chlorotic leaf spot virus* | Crab apple |  | Li et al. 2017b |
| *Trichovirus* | *Apple chlorotic leaf spot virus* | Peach |  | He et al. 2017 |
| *Trichovirus* | *Fig latent virus 1* | Fig |  | Chiumenti et al. 2014 |
| *Trichovirus* | *Grapevine berry inner necrosis virus* | Grapevine |  | Fan et al. 2017b |
| *Trichovirus* | *Grapevine berry inner necrosis virus* | Grapevine Beta |  | Fan et al. 2016b |
| *Trichovirus* | *Grapevine Pinot gris virus* | Grapevine | 21nt,22nt; both strands | Giampetruzzi et al. 2012; Glasa et al. 2014,2015; Saldarelli et al. 2015; Fan et al. 2016a; Eichmeier et al. 2016; Reynard et al. 2016; Czotter et al. 2018 |
| *Vitivirus* | *Actinidia virus A* | Kiwifruit  *A. chinensis* |  | Zheng et al. 2014 |
| *Vitivirus* | *Actinidia virus B* | *Actinidia* sp. | 21nt,22nt,24nt | Barrero et al. 2017 |
| *Vitivirus* | *Grapevine virus A* | Grapevine | 21nt,22nt; both strands | Czotter et al. 2018, pers com; Velasco et al. 2014; Maliogka et al. 2015; Eichmeier et al. 2016; Chiumenti et al. 2016b; Massart et al. 2018 |
| *Vitivirus* | *Grapevine virus B* | Grapevine | 21nt,22nt; (-) strand bias | Czotter et al. 2018, pers com; Barrero et al. 2017; Maliogka et al. 2015; Eichmeier et al. 2016; Cretazzo et al. 2017; Massart et al. 2018 |
| *Vitivirus* | *Grapevine virus G* | Grapevine |  | Blouin et al. 2018a,b |
| *Vitivirus* | *Grapevine virus I* | Grapevine |  | Blouin et al. 2018a,b |
| Unassigned  *Citrivirus*-like | *Watermelon virus A* | Watermelon |  | Xin et al. 2017b,c |
| Unassigned | *Cherry green ring mottle virus* | Peach |  | Lu et al. 2017 |
| Unassigned | *Banana mild mosaic virus* | Banana |  | Verdin et al. 2017 |
| **Bromoviridae**  (+)ssRNA |  |  |  |  |
| *Alfamovirus* | *Alfalfa mosaic virus* | *N. tabacum* |  | Pecman et al. 2017 |
| *Alfamovirus* | *Alfalfa mosaic virus* | Potato |  | Nie et al. 2015 |
| *Bromovirus* | *Brome mosaic virus* | Wheat |  | Sõmera et al. 2016 |
| *Cucumovirus* | *Cucumber mosaic virus*  and its 2b mutants | *A. thaliana*, its RNAi mutants | 21nt,22nt; both strands | Donaire et al. 2009; Wang et al. 2010; Wang et al. 2011 |
| *Cucumovirus* | *Cucumber mosaic virus* | Common bean |  | Mwaipopo et al. 2018 |
| *Cucumovirus* | *Cucumber mosaic virus* | Lily |  | Li et al. 2018 |
| *Cucumovirus* | *Cucumber mosaic virus* | Water lily |  | Kreuze 2014 |
| *Cucumovirus* | *Cucumber mosaic virus* | *N. tabacum* | 21nt,22nt; both strands | Shen et al. 2015; Zahid et al. 2015; Wang et al. 2015b; Akinyemi et al. 2016 |
| *Cucumovirus* | *Cucumber mosaic virus*  2b mutant | *N. benthamiana* | 21nt,22nt; (+) strand bias | Qiu et al. 2018 |
| *Cucumovirus* | *Cucumber mosaic virus*  2b mutant | *N. tabacum* | 21nt,22 nt; (+) strand bias | Qiu et al. 2017 |
| *Cucumovirus* | *Cucumber mosaic virus* | Tomato | 21nt,22 nt; (+) strand bias | Xu et al. 2017; Padmanabhan et al. 2015b; Che et al. 2018 |
| *Cucumovirus* | *Tomato aspermy virus* | Tomato | 21nt,22 nt; (+) strand bias | Xu et al. 2017 |
| *Cucumovirus* satellite | *Cucumber mosaic virus* satellite  Y-Sat | *N. benthamiana* | 21nt,22 nt; both strands | Shimura et al. 2011; Fang et al. 2015; Smith et al. 2011 |
| *Cucumovirus* satellite | *Cucumber mosaic virus* satellite Y-Sat | *N. tabacum* | 22nt,21nt | Shen et al. 2015 |
| *Cucumovirus*  endogenous | *Cucumber mosaic virus* satellite endogenous sequences | *N. tabacum* | 24nt; both strands | Zahid et al. 2015 |
| *Cucumovirus*  endogenous | *Cucumber mosaic virus* RNA1 endogenous sequences | Soybean | 22nt,24nt,21nt; both strands | Da Fonseca et al. 2016 |
| *Ilarvirus* | *Apple mosaic virus* | Sweet cherry |  | Ruiz-Garcia et al. 2016 |
| *Ilarvirus* | *Blackberry chlorotic ringspot virus* | Rose |  | He et al. 2015 |
| *Ilarvirus* | *Prune dwarf virus* | Sweet cherry |  | Ruiz-Garcia et al. 2016: Katsiani et al. 2018 |
| *Ilarvirus* | *Prunus necrotic ringspot virus* | Crab apple |  | Li et al. 2017b |
| *Ilarvirus* | *Prunus necrotic ringspot virus* | Cucumber | 21nt,22nt; both strands | Herranz et al. 2015 |
| *Ilarvirus* | *Prunus necrotic ringspot virus* | Peach | 21nt,22nt,24nt | Barrero et al. 2017 |
| *Ilarvirus* | *Prunus necrotic ringspot virus* | Rose |  | He et al. 2015 |
| *Ilarvirus* | *Prunus necrotic ringspot virus* | Sweet cherry | 21nt,22nt; both strands | Zhao and Song 2014a,b; Ruiz-Garcia et al. 2016; Wang et al. 2016e; Wang et al. 2018b |
| *Ilarvirus* | *Spinach latent virus* | Tomato |  | Xu et al. 2017 |
| *Ilarvirus* |  | Daisy |  | Verdin et al. 2017 |
| *Ilarvirus* |  | Wild sweetpea |  | Verdin et al. 2017 |
| **Caulimoviridae**  dsDNA-RT |  |  |  |  |
| *Cavemovirus* | *Sweet potato collusive virus* | Sweet potato |  | Mbanzibwa et al. 2014 |
| *Caulimovirus* | *Cauliflower mosaic virus* | *A. thaliana,* its RNAi mutants | 24nt,21nt,22nt; both strands | Blevins et al. 2011; Seguin et al. 2014 |
| *Caulimovirus* | *Cauliflower mosaic virus* | *Brassica oleracea* |  | Pecman et al. 2017 |
| *Caulimovirus* | Strawberry vein banding virus | Strawberry |  | Ding et al. 2016 |
| *Caulimovirus* |  | Centranthus |  | Verdin et al. 2017 |
| *Caulimovirus* |  | Common bean |  | Mwaipopo et al. 2018 |
| *Caulimovirus* |  | Lilac |  | Verdin et al. 2017 |
| *Badnavirus* episomal | *Banana streak Obino l'Ewai virus* | Banana Cavendish | 21nt,24nt,22nt; both strands | Rajeswaran et al. 2014b |
| *Badnavirus* episomal | *Banana streak Goldfinger virus* | Banana Cavendish | 21nt,22nt,24nt; both strands | Rajeswaran et al. 2014b |
| *Badnavirus* episomal | *Banana streak Imove virus* | Banana Cavendish | 21nt,22nt,24nt; both strands | Rajeswaran et al. 2014b |
| *Badnavirus* episomal | *Banana streak Mysore virus* | Banana Cavendish | 21nt,24nt,22nt; both strands | Rajeswaran et al. 2014b |
| *Badnavirus* episomal | *Banana streak Vietnam virus* | Banana Cavendish | 21nt,22nt,24nt; both strands | Rajeswaran et al. 2014b |
| *Badnavirus* episomal | *Banana streak Cavendish virus* | Banana Cavendish | 21nt,22nt,24nt; both strands | Rajeswaran et al. 2014b |
| *Badnavirus* | *Fig badnavirus-1* | Fig | 21nt,22nt,24nt; both strands | Turco, Reynard, Pooggin in prep; Chiumenti et al. 2014 |
| *Badnavirus* | *Grapevine Roditis leaf discoloration-associated virus* | Grapevine | 21nt,22nt,24nt; both strands | Maliogka et al. 2015; Chiumenti et al. 2016a |
| *Badnavirus* | *Grapevine vein clearing virus* | Grapevine | 21nt,22nt,24nt | Howard and Qiu 2017; Zhang et al. 2011 |
| *Badnavirus* | *Mulberry badnavirus 1* | Mulberry | 21nt,22nt,24nt; both strands | Chiumenti et al. 2016c |
| *Badnavirus* | *Pagoda yellow mosaic associated virus* | Pagoda tree | 21nt,22nt,24nt; both strands | Wang et al. 2014 |
| *Badnavirus* | *Rubus yellow net virus* | Raspberry | 21nt,22nt,24nt; (+) strand bias | Kalischuk et al. 2013;  Barrero et al. 2017 |
| *Badnavirus* | *Sweet potato badnavirus A* | Sweet potato |  | Mbanzibwa et al. 2014; Qin et al. 2016; Nhlapo et al. 2018 |
| *Badnavirus* | *Sweet potato badnavirus B* | Sweet potato | 22nt,24nt,21nt | Kreuze et al. 2009; Mbanzibwa et al. 2014; Qin et al. 2016; Nhlapo et al. 2018 |
| *Badnavirus* | *Sweet potato badnavirus C* | Sweet potato |  | Mbanzibwa et al. 2014 |
| *Badnavirus* | *Sweet potato pakakuy virus* | Sweet potato | 22nt,24nt,21nt | Kreuze et al. 2009; Kashif et al. 2012 |
| *Badnavirus* | *Taro bacilliform CH virus* | Taro | 21nt,22nt,24nt; both strands | Kazmi et al. 2015 |
| *Badnavirus* |  | Canna |  | Verdin et al. 2017 |
| *Florendovirus* endogenous | *Amborella trichopoda A virus* | *Amborella trichopoda* | both strands (21-24nt) | Geering et al. 2014 |
| *Florendovirus* endogenous | *Amborella trichopoda B virus* | *Amborella trichopoda* | both strands (21-24nt) | Geering et al. 2014 |
| *Florendovirus* endogenous | *Amborella trichopoda C virus* | *Amborella trichopoda* | both strands (21-24nt) | Geering et al. 2014 |
| *Florendovirus* endogenous | *Citrus clementina virus* | Clementine |  | Geering et al. 2014 |
| *Florendovirus* endogenous | *Citrus clementina virus* | Sweet orange |  | Geering et al. 2014 |
| *Florendovirus* endogenous | *Eucalyptus grandis florendovirus 1* | *Eucalyptus grandis* | 24nt,22nt,21nt | Marcon et al. 2017 |
| *Florendovirus* endogenous | *Eucalyptus grandis florendovirus 4* | *Eucalyptus grandis* | 24nt,22nt,21nt | Marcon et al. 2017 |
| *Florendovirus* endogenous | *Populus trichocarpa virus* | Poplar |  | Geering et al. 2014 |
| *Florendovirus* endogenous | *Solanum tuberosum virus* | Potato |  | Geering et al. 2014 |
| *Florendovirus* endogenous | *Sorghum bicolor virus* | *Sorghum bicolor* |  | Geering et al. 2014 |
| *Florendovirus* endogenous | *Solanum lycopersicum virus* | Tomato |  | Geering et al. 2014 |
| *Florendovirus* endogenous | *Vitis vinifera A virus* | Grapevine | both strands (21-24nt) | Geering et al. 2014 |
| *Florendovirus* endogenous | *Vitis vinifera B virus* | Grapevine | both strands (21-24nt) | Geering et al. 2014 |
| *Petuvirus*  endogenous ? | *Petunia vein clearing virus* | Petunia |  | Verdin et al. 2017 |
| *Solendovirus* | *Tobacco vein-clearing virus* | *N. tabacum* | 21nt,22nt | Barrero et al. 2017 |
| *Solendovirus*  endogenous | *Tobacco vein-clearing virus-*like | *N. tabacum* |  | Kreuze 2014 |
| *Solendovirus*  endogenous | *Tobacco vein-clearing virus-*like | *Physalis ﬂoridiana* |  | Kreuze 2014 |
| *Solendovirus*  endogenous | *Tobacco vein-clearing virus-*like | Potato |  | Kreuze 2014 |
| *Solendovirus*  endogenous | *Tobacco vein-clearing virus-*like | Tomato |  | Kreuze 2014 |
| *Tungrovirus* episomal | *Rice tungro bacilliform virus* | Rice | 21nt,22nt,24nt; both strands | Rajeswaran et al. 2014a; Zarreen et al. 2018 |
| Endogenous  *Petuvirus*-like | *Citrus endogenous pararetrovirus* | *Citrus* sp. | 24nt,21nt,22nt | Barrero et al. 2017 |
| Endogenous *Petuvirus*-like | *Citrus endogenous pararetrovirus* | Sweet orange |  | Matsumura et al. 2017 |
| Endogenous *Petuvirus*-like | *Eucalyptus grandis endogenous viral element 1* | *Eucalyptus grandis* | 24nt,21nt,22nt | Marcon et al. 2017 |
| Endogenous *Petuvirus*-like | *Fritillaria imperialis endogenous pararetrovirus* | *Fritillaria imperialis* | 24nt,22nt,21nt | Becher et al. 2014 |
| Endogenous *Petuvirus*-like |  | Milkweed |  | Verdin et al. 2017 |
| Endogenous  *Badnavirus-*like |  | Banana  *M. acuminata* |  | Kreuze 2014 |
| Endogenous *Caulimo-Sobemo*-like |  | Poplar |  | Kreuze 2014 |
| Endogenous *Cavemo-Badna-*like |  | Watermelon |  | Zaagueri et al. 2017 |
| Endogenous |  | *Amborella trichopoda* |  | Kreuze 2014 |
| Endogenous |  | *Chara coralline* |  | Kreuze 2014 |
| Endogenous |  | *Cycas rumphii* |  | Kreuze 2014 |
| Endogenous |  | Moss  *P. patens* |  | Kreuze 2014 |
| Endogenous |  | Pomelo |  | Verdin et al. 2017 |
| Endogenous |  | *Silene latifolium* |  | Kreuze 2014 |
| Endogenous |  | Sweet orange |  | Kreuze 2014 |
| Endogenous |  | Water lily |  | Kreuze 2014 |
| **Closteroviridae**  (+)ssRNA |  |  |  |  |
| *Ampelovirus* | *Fig leaf mottle-associated virus 2* | Fig | 21nt,22nt; both strands | Turco, Reynard, Pooggin in prep |
| *Ampelovirus* | *Grapevine leafroll-associated virus 1* | Grapevine | 21nt,22nt; both strands | Czotter et al. 2018, pers com; Eichmeier et al. 2016; Chiumenti et al. 2016b; |
| *Ampelovirus* | *Grapevine leafroll‐associated virus 3* | Grapevine | 21nt,22nt; both strands | Alabi et al. 2012; Maliogka et al. 2015; Velasco et al. 2014; Visser et al. 2016; Chiumenti et al. 2016b; Bester et al. 2017a,b; Barrero et al. 2017; Czotter et al. 2018; Fan et al. 2017b |
| *Ampelovirus* | *Grapevine leafroll‐associated virus 3* | Grapevine Beta |  | Fan et al. 2017a; Ahmed et al. 2018 |
| *Ampelovirus* | *Grapevine leafroll‐associated virus 4* | Grapevine | 21nt,22nt; both strands | Turco, Reynard, Pooggin in prep; Velasco et al. 2014 |
| *Ampelovirus* | *Little cherry virus 2* | Sweet cherry | 21nt,22nt; both strands | Turco, Reynard, Pooggin in prep |
| *Ampelovirus* | *Plum bark necrosis stem pitting-associated virus* | Peach |  | He et al. 2017; Lu et al. 2017 |
| *Ampelovirus* | *Plum bark necrosis stem pitting-associated virus* | Sweet cherry |  | Wang et al. 2016c; Zamorano et al. 2017 |
| *Crinivirus* | *Bean yellow disorder virus* | Common bean |  | Mwaipopo et al. 2018 |
| *Crinivirus* | *Beet pseudo yellows virus* | Lettuce |  | Kreuze 2014 |
| *Crinivirus* | *Lettuce chlorosis virus* | Green bean | 21nt,22nt; both strands | Ruiz et al. 2018 |
| *Crinivirus* | *Lettuce chlorosis virus* | Tomato |  | Zhang et al. 2017b |
| *Crinivirus* | *Lettuce infectious yellows virus* | Lettuce |  | Kreuze 2014 |
| *Crinivirus* | *Lettuce infectious yellows virus* | *N. benthamiana* | 22nt,21nt; both strands | Qiao et al. 2018 |
| *Crinivirus* | *Strawberry crinivirus 4* | Strawberry |  | Ding et al. 2016 |
| *Crinivirus* | *Strawberry pallidosis associated virus* | Strawberry |  | Ding et al. 2016, 2017 |
| *Crinivirus* | *Sweet potato chlorotic stunt virus* | Sweet potato | 22nt,21nt; both strands | Kreuze et al. 2009; Cuellar et al. 2015; Cuellar et al. 2011; Kashif et al. 2012; Mbanzibwa et al. 2014; Untiveros et al. 2016; Cao et al. 2017a |
| *Crinivirus* | *Tomato chlorosis virus* | Tomato | 22nt,21nt; both strands | Xu et al. 2017; Padmanabhan et al. 2015b; Pecman et al. 2017 |
| *Closterovirus* | *Cereal closterovirus* | Oat, Wheat |  | Sõmera et al. in prep |
| *Closterovirus* | *Citrus tristeza virus* | Citron | 21nt,22nt,24nt | Barrero et al. 2017 |
| *Closterovirus* | *Citrus tristeza virus* | *Citrus macrophylla* | both strands | Folimonova et al. 2014 |
| *Closterovirus* | *Citrus tristeza virus* | *Citrus* sp. |  | Roy et al. 2013b |
| *Closterovirus* | *Citrus tristeza virus* | Grapefruit | 22nt,21nt; both strands | Visser et al. 2017; Visser et al. 2016 |
| *Closterovirus* | *Citrus tristeza virus* | Lemon | (-) strand bias | Varveri et al. 2015 |
| *Closterovirus* | *Citrus tristeza virus* | Mandarin | both strands | Beris, Varveri, et al. in prep |
| *Closterovirus* | *Citrus tristeza virus* | Mexican lime | 21nt,22nt; (+) strand bias | Ruiz-Ruiz et al. 2011 |
| *Closterovirus* | *Citrus tristeza virus* | Persian lime | 21nt,22nt | Barrero et al. 2017 |
| *Closterovirus* | *Citrus tristeza virus* | Sour orange | 21nt,22nt; (+) strand bias | Ruiz-Ruiz et al. 2011 |
| *Closterovirus* | *Citrus tristeza virus* | Sweet orange | 21nt,22nt; (+) strand bias | Ruiz-Ruiz et al. 2011; Roy et al. 2013a; Matsumura et al. 2017 |
| *Closterovirus* | *Citrus tristeza virus* | Sweet orange on sour orange | 21nt,22nt; both strands; (-) strand bias | Licciardello et al. 2015 |
| *Closterovirus* | *Citrus tristeza virus* | Trifoliate orange | 22nt,21nt; (+) strand bias | Yokomi et al. 2017 |
| *Closterovirus* | *Grapevine leafroll-associated virus 2* | Grapevine | 21nt,22nt; both strands | Seguin, Dolya, Pooggin in prep; Chiumenti et al. 2016b; Cretazzo et al. 2017 |
| *Closterovirus* | *Rose leaf rosette-associated virus* | Rose | 21nt,22nt; both strands | He et al. 2015 |
| *Closterovirus* | *Fig mild mottle-associated virus* | Fig | 21nt,22nt; both strands | Turco, Reynard, Pooggin in prep; Chiumenti et al. 2014 |
| *Velarivirus* | *Grapevine leafroll-associated virus 7* | Grapevine |  | Ma et al. 2017 |
| *Velarivirus* | *Little cherry virus 1* | Apricot | 21nt,22nt; both strands | Baráth et al. 2018 |
| *Velarivirus* | *Little cherry virus 1* | Sweet cherry | 21nt,22nt; both strands | Katsiani et al. 2018; Turco, Reynard, Pooggin in prep; Wang et al. 2016d; Ruiz-Garcia et al. 2016; Wang et al. 2016e; Koloniuk et al. 2018; Fiore et al. 2018 |
| **Endornaviridae**  dsRNA |  |  |  |  |
| *Alphaendornavirus* | *Bell pepper endornavirus* | Bell pepper |  | Sela et al. 2012; Kreuze 2014; Chen et al. 2015a; Kamran et al. 2018 |
| *Alphaendornavirus* | *Helianthus annuus alphaendornavirus* | Sunflower | 21nt,22nt; both strands | Liu et al. 2018 |
| *Alphaendornavirus* | *Hordeum vulgare endornavirus* | Barley | 21nt,22nt; both strands | Golyaev, Candresse, Pooggin et al. in prep; Sõmera et al. in prep |
| *Alphaendornavirus* | *Phaseolus vulgaris endornavirus 1* | Common bean | both strands | Nordenstedt et al. 2017; Mwaipopo et al. 2018 |
| *Alphaendornavirus* | *Phaseolus vulgaris endornavirus 2* | Common bean | both strands | Nordenstedt et al. 2017; Mwaipopo et al. 2018 |
| *Alphaendornavirus* |  | Anemone |  | Verdin et al. 2017 |
| *Alphaendornavirus* |  | Periwinkle |  | Verdin et al. 2017 |
| **Fimoviridae**  (-)ssRNA |  |  |  |  |
| *Emaravirus* | *Actinidia chlorotic ringspot-associated virus* | Kiwifruit  *A. chinensis* | 21nt,22nt; both strands | Zheng et al. 2017b |
| *Emaravirus* | *Fig mosaic virus* | Fig | 21nt,22nt; both strands | Turco, Reynard, Pooggin in prep Chiumenti et al. 2014 |
| *Emaravirus* | *Pigeonpea sterility mosaic virus-I* | Pigeonpea | 21nt,22nt,24nt; both strands | Patil and Arora 2018; Kumar et al. 2017 |
| *Emaravirus* | *Pigeonpea sterility mosaic virus-II* | Pigeonpea |  | Kumar et al. 2017 |
| *Emaravirus* | *Raspberry leaf blotch virus* | Raspberry |  | Bi et al. 2012a |
| *Emaravirus* | *Woolly burdock yellow vein virus* | Woolly burdock | 21nt,22nt | Bi et al. 2012b |
| **Geminiviridae**  ssDNA |  |  |  |  |
| *Capulavirus* | *Plantago lanceolata latent virus* | *Plantago lanceolata* |  | Susi et al. 2017 |
| *Grablovirus* | *Grapevine red blotch virus* | Grapevine | 21nt,24nt,22nt; both strands | Seguin et al. 2014 |
| *Begomovirus* endogenous | *Endogenous geminivirus-like element 1* | Water yam | 21nt,24nt,22nt; both strands | Filloux et al. 2015 |
| *Begomovirus* bipartite | *African cassava mosaic virus* | Cassava | 21nt,22nt,24nt; both strands | Kuria et al. 2017 |
| *Begomovirus* bipartite | *Cabbage leaf curl virus*  & CP deletion/insertion mutants | *A. thaliana* & RNAi mutants | 21nt,24nt,22nt; both strands | Aregger et al. 2012;  Seguin et al. 2014 |
| *Begomovirus* bipartite | *East African cassava mosaic virus* | Cassava | 21nt,22nt,24nt; both strands | Kuria et al. 2017 |
| *Begomovirus* bipartite | *Indian cassava mosaic virus* | Cassava | 21nt,22nt,24nt; both strands | Karthikeyan et al. 2016, Pooggin et al. in prep |
| *Begomovirus* bipartite | *Mungbean yellow mosaic India virus* | Pigeonpea | 22nt,24nt,21nt; both strands | Patil and Arora 2018 |
| *Begomovirus* bipartite | *Squash leaf curl virus* | Squash |  | Hagen et al. 2011 |
| *Begomovirus* bipartite | *South African cassava mosaic virus* | Cassava | 24nt,21nt,22nt; both strands | Rogans et al. 2016 |
| *Begomovirus* bipartite | *Sri Lankan cassava mosaic virus* | Cassava | 24nt,21nt,22nt; both strands | Karthikeyan et al. 2016, Pooggin et al. in prep |
| *Begomovirus* monopartite | *Cotton leaf curl Multan virus* | Cotton | 21nt,22nt,24nt; both strands | Wang et al. 2016a |
| *Begomovirus* monopartite | *Sweet potato golden vein associated virus* | Sweet potato |  | Mbanzibwa et al. 2014; Qin et al. 2016 |
| *Begomovirus* bipartite | *Sweet potato leaf curl virus* | Sweet potato | 22nt,21nt,24nt; both strands | Cuellar et al. 2015; Mbanzibwa et al. 2014; Qin et al. 2016; Cao et al. 2017a |
| *Begomovirus* bipartite | *Sweet potato leaf curl Bengal virus* | Sweet potato |  | Qin et al. 2016 |
| *Begomovirus* bipartite | *Sweet potato leaf curl Canary virus* | Sweet potato |  | Qin et al. 2016 |
| *Begomovirus* monopartite | *Sweet potato leaf curl Georgia virus* | Sweet potato |  | Kashif et al. 2012; Qin et al. 2016 |
| *Begomovirus* monopartite | *Sweet potato leaf curl Lanzarote virus* | Sweet potato |  | Qin et al. 2016 |
| *Begomovirus* monopartite | *Sweet potato leaf curl Sao Paulo virus* | Sweet potato |  | Mbanzibwa et al. 2014 |
| *Begomovirus* monopartite | *Sweet potato leaf curl Spain virus* | Sweet potato |  | Mbanzibwa et al. 2014 |
| *Begomovirus* monopartite | *Sweet potato leaf curl Uganda virus* | Sweet potato |  | Mbanzibwa et al. 2014; Qin et al. 2016 |
| *Begomovirus* monopartite | *Sweet potato mosaic virus* | Sweet potato |  | Mbanzibwa et al. 2014 |
| *Begomovirus* monopartite | *Tomato latent virus* | Tomato RNAi-transgenic | 22nt,21nt,24nt; both strands | Fuentes et al. 2016 |
| *Begomovirus* monopartite | *Tomato yellow leaf curl China virus* | *N. benthamiana* | 22nt,21nt,24nt; both strands | Yang et al. 2011 |
| *Begomovirus* monopartite | *Tomato yellow leaf curl China virus* | Tomato | 22nt,21nt,24nt; both strands | Yang et al. 2011 |
| *Begomovirus* monopartite | *Tomato yellow leaf curl Sardinia virus* | Tomato | 21nt,22nt,24nt; both strands | Miozzi et al. 2013b |
| *Begomovirus* monopartite | *Tomato yellow leaf curl virus* | Tomato | 21nt,22nt,24nt; both strands | Donaire et al. 2009; Fuentes et al. 2016; Xu et al. 2017; Padmanabhan et al. 2015b; Leibman et al. 2015; Pecman et al. 2017; Che et al. 2018 |
| *Begomovirus* | *Sweet potato begomovirus* | Sweet potato |  | Mbanzibwa et al. 2014 |
| *Begomovirus* |  | Common bean |  | Mwaipopo et al. 2018 |
| *Begomovirus* |  | Lilac |  | Verdin et al. 2017 |
| *Begomovirus*  satellite | *Cotton leaf curl Multan*  *betasatellite* | Cotton | 21nt,22nt,24nt; both strands | Wang et al. 2016a |
| *Begomovirus*  satellite | *Tomato yellow leaf curl China betasatellite* | *N. benthamiana* | 22nt,21nt,24nt; both strands | Yang et al. 2011 |
| *Begomovirus*  satellite | *Tomato yellow leaf curl China betasatellite* | Tomato | 22nt,21nt,24nt; both strands | Yang et al. 2011 |
| *Mastrevirus* | *Chickpea chlorotic dwarf virus* | Watermelon | both strands | Zaagueri et al. 2017 |
| *Mastrevirus* | *Maize streak Reunion virus* | Maize | 24nt,22nt,21nt; both strands | Chen et al. 2015b |
| *Mastrevirus* | *Sugarcane streak Egypt Virus* | Sugarcane | 24nt,21nt,22nt; both strands | Candresse et al. 2014 |
| *Mastrevirus* | *Sugarcane white streak virus* | Sugarcane | 24nt,21nt,22nt; both strands | Candresse et al. 2014 |
| *Mastrevirus* | *Sweet potato symptomless mastrevirus 1* | Sweet potato | 22nt,24nt,21nt | Kreuze et al. 2009; Wang et al. 2015a; Cao et al. 2017a; Barrero et al. 2017 |
| *Mastrevirus* | *Wheat dwarf virus* | Wheat |  | Sõmera et al., in prep |
| Unassigned | *Apple geminivirus* | Apple | 21nt,22nt,24nt; both strands | Liang et al. 2015 |
| Unassigned | *Camellia chlorotic dwarf-associated virus* | Camellia |  | Zhang et al. 2018 |
| Unassigned | *Citrus chlorotic dwarf-associated virus* | Lemon |  | Loconsole et al. 2012b |
| Unassigned | *Grapevine geminivirus A* | Grapevine |  | Fan et al. 2017b; Ahmed et al. 2018 |
| Unassigned | *Mulberry mosaic dwarf-associated virus* | Mulberry |  | Ma et al. 2015a |
| **Luteoviridae**  (+)ssRNA |  |  |  |  |
| *Enamovirus* | *Citrus vein enation virus* | Etrog citron | 21nt,24nt,22nt; both strands | Vives et al. 2013 |
| *Enamovirus* | *Pea enation virus* | Wild sweetpea |  | Verdin et al. 2017 |
| *Luteovirus* | *Barley yellow dwarf virus* | Barley, Oat, Rye, Wheat |  | Sõmera et al. in prep |
| *Luteovirus* |  | Wild sweetpea |  | Verdin et al. 2017 |
| *Polerovirus* | *Cereal yellow dwarf virus* | Cocksfoot |  | Pallett et al. 2010 |
| *Polerovirus* | *Cereal yellow dwarf virus* | Oat |  | Sõmera et al. in prep |
| *Polerovirus* | *Cucurbit aphid-borne yellows virus* | Pumpkin |  | Tang et al. 2017 |
| *Polerovirus* | *Brassica yellows virus* | *N. benthamiana* | 22nt,21nt; both strands | Zhou et al. 2017 |
| *Polerovirus* | *Brassica yellows virus* | *N. tabacum* | both strands | Akinyemi et al. 2016 |
| *Polerovirus* | *Cassava polero-like virus* | Cassava |  | Carvajal-Yepes et al. 2014 |
| *Polerovirus* | *Cotton leafroll dwarf virus* | Cotton | 22nt,21nt,24nt; both strands | Silva et al. 2011;  Romanel et al. 2012 |
| *Polerovirus* | *Maize yellow mosaic virus* | Maize | 22nt,21nt; both strands | Chen et al. 2016 |
| *Polerovirus* | *Maize yellow dwarf virus* | Maize |  | Wang et al. 2016f |
| *Polerovirus* | *Melon aphid-borne yellows virus* | Pumpkin |  | Tang et al. 2017 |
| *Polerovirus* | *Pepper leafroll chlorosis virus* | Bell pepper | 21nt,22nt; both strands | Kamran et al. 2018 |
| *Polerovirus* | *Pepper vein yellows virus* | Pepper |  | Liu et al. 2016a |
| *Polerovirus* | *Pepper vein yellows virus* | Red pepper |  | Zhang et al. 2015 |
| *Polerovirus* | *Pepper yellows virus* | Pepper | 21nt,22nt; both strands | Lotos et al. 2017 |
| *Polerovirus* | *Potato leafroll virus* | Potato | 21nt,22nt,24nt; both strands | Hwang et al. 2013; Zheng et al. 2017a |
| *Polerovirus* | *Strawberry polerovirus 1* | Strawberry |  | Ding et al. 2016 |
| *Polerovirus* | *Turnip yellows virus* | European waterclover |  | Kreuze 2014 |
| *Polerovirus* | *Turnip yellows virus* | *N. tabacum* |  | Wang et al. 2015b |
| *Polerovirus* | *Turnip yellows virus* | Tomato |  | Xu et al. 2017 |
| *Polerovirus* | *Wheat leaf yellowing-associated virus* | Wheat | 21nt,22nt; both strands | Zhang et al. 2017a |
| *Polerovirus* |  | Iris |  | Verdin et al. 2017 |
| *Polerovirus* |  | Periwinkle |  | Verdin et al. 2017 |
| *Polerovirus-*like |  | *Torilis arvensis* |  | Lotos et al. 2018 |
| Unassigned | *Nectarine stem pitting-associated virus* | Peach |  | Lu et al. 2017 |
| **Nanoviridae**  ssDNA |  |  |  |  |
| *Nanovirus* | *Faba bean necrotic stunt virus* | Faba bean | 24nt,22nt,21nt; both strands | Golyaev, Blanc, Pooggin et al. in prep |
| *Nanovirus* | *Pea necrotic yellow dwarf virus* | Pea |  | Pecman et al. 2017 |
| **Metaviridae**  ssRNA-RT |  |  |  |  |
| *Ty3/Gypsy*  endogenous | LTR retrotransposons | *A. thaliana*, Maize, Mangrove, Strawberry, Wheat | both strands; (21-24nt) | Creasey et al. 2014; Masuta et al. 2017; Alejandri-Ramírez et al. 2018; Wang et al. 2018a; Šurbanovski et al. 2016; Sun et al. 2013 |
| **Partitiviridae**  dsRNA |  |  |  |  |
| *Deltapartitivirus* | *Citrullus lanatus cryptic virus* | Watermelon | 21nt,22nt; both strands | Xin et al. 2017b |
| *Deltapartitivirus* putative | *Persimmon cryptic virus* | Japanese persimmon |  | Ruiz-Garcia et al. 2017 |
| *Deltapartitivirus* |  | Anemone |  | Verdin et al. 2017 |
| *Deltapartitivirus* |  | Carnation |  | Verdin et al. 2017 |
| **Phenuiviridae**  (-)ssRNA |  |  |  |  |
| *Tenuivirus* | *European wheat striate mosaic virus* | Oat, Wheat |  | Sõmera et al. in prep |
| *Tenuivirus* | *Rice hoja blanca virus* | Rice |  | Jimenez et al. 2018 |
| *Tenuivirus* | *Rice stripe virus* | *N. benthamiana* | 21nt,20nt,22nt; both strands | Xu et al. 2012 |
| *Tenuivirus* | *Rice stripe virus* | Rice | 21nt,20nt,22nt; both strands | Yan et al. 2010; Xu et al. 2012; Jiang et al. 2012; Wu et al. 2015b |
| Unclassified Bunyavirales | *Watermelon crinkle leaf-associated virus 1* | Watermelon |  | Xin et al. 2017a |
| Unclassified Bunyavirales | *Watermelon crinkle leaf-associated virus 2* | Watermelon |  | Xin et al. 2017a |
| **Potyviridae**  (+)ssRNA |  |  |  |  |
| *Bymovirus* | *Wheat yellow mosaic virus* | Wheat | 21nt,22nt; both strands | Chen et al. 2014; Li et al. 2017a |
| *Ipomovirus* | *Cassava brown streak viruses* | Cassava | 21nt,22nt; both strands | Ogwok et al. 2016 |
| *Ipomovirus* | *Cucumber vein yellowing virus* | Cucumber |  | Velasco et al. 2016 |
| *Ipomovirus* | *Squash vein yellowing virus* | Watermelon | 21nt,22nt; both strands | Reingold et al. 2016 |
| *Ipomovirus* | *Sweet potato mild mottle virus* | Sweet potato |  | Mbanzibwa et al. 2014 |
| *Macluravirus* | *Alpinia oxyphylla mosaic virus* | *Alpinia oxyphylla* |  | Hu et al. 2018 |
| *Poacevirus* | *Triticum mosaic virus* | Wheat | 21nt,22nt; both strands | Tatineni et al. 2014 |
| *Potyvirus* | *Alstroemeria mosaic virus* | Alstroemeria |  | Verdin et al. 2017 |
| *Potyvirus* | *Apium virus Y* | Parsley | 21nt,22nt; both strands | Mehle et al. in prep |
| *Potyvirus* | *Bean common mosaic virus* | Common bean |  | Mwaipopo et al. 2018 |
| *Potyvirus* | *Bean common mosaic virus* | Sesame |  | Shi et al. 2016; Wang et al. 2017 |
| *Potyvirus* | *Bean common mosaic necrosis virus* | Common bean |  | Chiquito-Almanza et al. 2018; Mwaipopo et al. 2018 |
| *Potyvirus* | *Bean yellow mosaic virus* | Canna |  | Verdin et al. 2017 |
| *Potyvirus* | *Canna yellow streak virus* | Canna |  | Verdin et al. 2017 |
| *Potyvirus* | *Carrot thin leaf virus* | Parsley | 21nt,22nt; both strands | Mehle et al. in prep |
| *Potyvirus* | *Carrot thin leaf virus* | *Torilis arvensis* | both strands | Lotos et al. 2018 |
| *Potyvirus* | *Chilli veinal mottle virus* | Tomato | 21nt,22nt; both strands | Xu et al. 2017 |
| *Potyvirus* | *Cocksfoot streak virus* | Cocksfoot | 21nt,22nt; both strands | Ho et al. 2010 |
| *Potyvirus* | *Cowpea aphid-borne mosaic virus* | Common bean |  | Mwaipopo et al. 2018 |
| *Potyvirus* | *Endive necrotic mosaic virus* | Lettuce |  | Desbiez et al. 2017a |
| *Potyvirus* | *Henbane mosaic virus* | Tomato | 21nt,22nt; both strands | Pecman et al. in prep |
| *Potyvirus* | *Iris mild mosaic virus* | Iris |  | Verdin et al. 2017 |
| *Potyvirus* | *Iris severe mosaic virus* | Iris |  | Li et al. 2016b |
| *Potyvirus* | *Japanese yam mosaic virus* | Pumpkin |  | Tang et al. 2017 |
| *Potyvirus* | *Kalanchoe mosaic virus* | Kalanchoe |  | Verdin et al. 2017 |
| *Potyvirus* | *Lettuce Italian necrotic virus* | Lettuce |  | Desbiez et al. 2017a |
| *Potyvirus* | *Lily yellow mosaic virus* | Lily |  | Li et al. 2018 |
| *Potyvirus* | *Ornithogalum mosaic virus* | Iris |  | Verdin et al. 2017 |
| *Potyvirus* | *Papaya leaf distortion mosaic virus* | Papaya | 21-nt,22nt; both strands | Zhao et al. 2018 |
| *Potyvirus* | *Papaya ringspot virus* | Papaya |  | Zhang et al. 2014b; Kreuze 2014 |
| *Potyvirus* | *Peanut mottle virus* | Common bean |  | Mwaipopo et al. 2018 |
| *Potyvirus* | *Peanut mottle virus* | Peanut |  | An et al. 2018 |
| *Potyvirus* | *Pecan mosaic-associated virus* | Pecan | 21nt,22nt; both strands | Su et al. 2016 |
| *Potyvirus* | *Pepper mottle virus* | *N. tabacum* | both strands | Akinyemi et al. 2016 |
| *Potyvirus* | *Pepper mottle virus* | Tomato | 21nt,22nt; both strands | Xu et al. 2017 |
| *Potyvirus* | *Pepper veinal mottle virus* | Bell pepper |  | Zhang et al. 2016b |
| *Potyvirus* | *Pepper veinal mottle virus* | Tomato | 21nt,22nt; both strands | Xu et al. 2017 |
| *Potyvirus* | *Plum pox virus* | Apricot |  | Baráth et al. 2018 |
| *Potyvirus* | *Plum pox virus* | Japanese plum | 22nt,21nt; both strands | Sánchez et al. 2017 |
| *Potyvirus* | *Plum pox virus* | *N. benthamiana* | 21nt,22nt; both strands | Montes et al. 2014 |
| *Potyvirus* | *Potato virus A* | Potato |  | Zheng et al. 2017a; Wu et al. 2018b |
| *Potyvirus* | *Potato virus A* derivatives | *N. tabacum* |  | Santala and Valkonen 2018 |
| *Potyvirus* | *Potato virus A* | Tomato | 21nt,22nt; both strands | Xu et al. 2017 |
| *Potyvirus* | *Potato virus Y* | *N. tabacum* | 21nt,22nt; both strands | Barrero et al. 2017; Akinyemi et al. 2016; Santala and Valkonen 2018; Wang et al. 2015b |
| *Potyvirus* | *Potato virus Y* | Potato | 21nt,22nt; both strands | Naveed et al. 2014; Kutnjak et al. 2015; Nie et al. 2015; Pecman et al. 2017; Turco et al. 2018 |
| *Potyvirus* | *Potato virus Y* | Tomato | 21nt,22nt; both strands | Xu et al. 2017; Frizzi et al. 2014 |
| *Potyvirus* | *Potato yellow blotch virus* | Potato |  | Nisbet et al. 2018 |
| *Potyvirus* | *Ranunculus leaf distortion virus* | Buttercup |  | Verdin et al. 2017 |
| *Potyvirus* | *Ranunculus mild mosaic virus* | Buttercup |  | Verdin et al. 2017 |
| *Potyvirus* | *Sorghum mosaic virus* | *Miscanthus sinenesis* | 21nt,22nt,24nt | Barrero et al. 2017 |
| *Potyvirus* | *Soybean mosaic virus* | Pumpkin |  | Tang et al. 2017 |
| *Potyvirus* | *Sudan watermelon mosaic virus* | *Cucumis melo* |  | Desbiez et al. 2017b |
| *Potyvirus* | *Sugarcane mosaic virus* | Maize | 21nt,22nt; both strands | Xia et al. 2014; Xia et al. 2016 |
| *Potyvirus* | *Sweet potato feathery mottle virus* | Sweet potato | 22nt,21nt,24nt;  both strands | Kreuze et al. 2009; Kreuze 2014; Kashif et al. 2012; Mbanzibwa et al. 2014; Cuellar et al. 2015; Untiveros et al. 2016; Qin et al. 2016; Cao et al. 2017a |
| *Potyvirus* | *Sweet potato virus 2* | Sweet potato |  | Mbanzibwa et al. 2014; Untiveros et al. 2016: Qin et al. 2016; Cao et al. 2017a |
| *Potyvirus* | *Sweet potato virus C* | Sweet potato |  | Kashif et al. 2012; Qin et al. 2016; Cao et al. 2017a |
| *Potyvirus* | *Sweet potato virus G* | Sweet potato |  | Mbanzibwa et al. 2014; Untiveros et al. 2016; Qin et al. 2016; Cao et al. 2017a |
| *Potyvirus* | *Tobacco vein banding mosaic virus* | *N. tabacum* | both strands | Akinyemi et al. 2016; Wang et al. 2015b |
| *Potyvirus* | *Tobacco vein banding mosaic virus* | Sesame |  | Wang et al. 2017 |
| *Potyvirus* | *Tobacco vein banding mosaic virus* | Tomato |  | Xu et al. 2017 |
| *Potyvirus* | *Tomato necrotic stunt virus* | Tomato | 21nt,22nt; (+) strand bias | Li et al. 2012 |
| *Potyvirus* | *Turnip mosaic virus*  & its suppressor mutant | *A. thaliana* & RNAi mutants | 21nt,22nt; both strands | Donaire et al. 2009; Garcia-Ruiz et al. 2010, 2015; Kung et al. 2014; Aguiar et al. 2015 |
| *Potyvirus* | *Turnip mosaic virus* | *Brassica juncea* | 21nt,22nt; both strands | Ho et al. 2010 |
| *Potyvirus* | *Watermelon mosaic virus* | Crepe myrtle |  | Wu et al. 2018a |
| *Potyvirus* | *Watermelon mosaic virus* | *Cucumis melo* | 21nt,22nt; both strands | Donaire et al. 2009 |
| *Potyvirus* | *Watermelon mosaic virus* | Melon | both strands | Frizzi et al. 2014 |
| *Potyvirus* | *Watermelon mosaic virus* | Sesame |  | Shi et al. 2016; Wang et al. 2017 |
| *Potyvirus* | *Watermelon mosaic virus* | Pumpkin |  | Tang et al. 2017 |
| *Potyvirus* | *Watermelon mosaic virus* | Watermelon | both strands | Zaagueri et al. 2017 |
| *Potyvirus* | *Watermelon mosaic virus* | Zucchini | both strands | Frizzi et al. 2014; Sun et al. 2018 |
| *Potyvirus* | *Yam bean mosaic virus* | Yam bean | 21nt,22nt; both strands | Fuentes et al. 2012 |
| *Potyvirus* | *Zucchini yellow mosaic virus* | Cucumber | 22nt,21nt; both strands | Leibman et al. 2011 |
| *Potyvirus* | *Zucchini yellow mosaic virus* | Melon | 22nt,21nt; both strands | Leibman et al. 2011 |
| *Potyvirus* | *Zucchini yellow mosaic virus* | Sesame |  | Shi et al. 2016 |
| *Potyvirus* | *Zucchini yellow mosaic virus* | Pumpkin |  | Tang et al. 2017 |
| *Potyvirus* | *Zucchini yellow mosaic virus* | Waterelon | 22nt,21nt; both strands | Kaldis et al. 2018 |
| *Potyvirus* |  | weed | 21nt,22nt; both strands | Zheng et al. 2017a |
| *Potyvirus* |  | Alstroemeria |  | Verdin et al. 2017 |
| *Potyvirus* |  | Anemon |  | Verdin et al. 2017 |
| *Potyvirus* |  | Buttercup |  | Verdin et al. 2017 |
| *Potyvirus* |  | Hydrangea |  | Verdin et al. 2017 |
| *Potyvirus* |  | Petunia |  | Verdin et al. 2017 |
| *Tritimovirus* | *Cocksfoot steak mosaic virus* | Cocksfoot |  | Sõmera et al. in prep |
| *Tritimovirus* | *Wheat streak mosaic virus* | Wheat | 21nt,22nt; both strands | Tatineni et al. 2014 |
| Unassigned | *Areca palm necrotic ringspot virus* | Areca palm |  | Yang et al. 2018b |
| Unassigned | *Common reed chlorotic stripe virus* | Common reed |  | Yuan et al. 2017 |
| **Pseudoviridae**  ssRNA-RT |  |  |  |  |
| *Ty1/Copia*  endogenous | LTR retrotransposons | *A. thaliana*, Maize, Mangrove, Strawberry, Wheat  Brown alga | both strands; (21-24nt)  21nt,20nt; both strands | Creasey et al. 2014; Masuta et al. 2017; Alejandri-Ramírez et al. 2018; Wang et al. 2018a; Šurbanovski et al. 2016;  Sun et al. 2013  Waldron et al. 2018 |
| **Reovirirdae**  dsRNA |  |  |  |  |
| *Fijivirus* | *Mal de Río Cuarto virus* | Wheat | 21nt,22nt; both strands | de Haro et al. 2017 |
| *Fijivirus* | *Oat sterile dwarf virus* | Oat, Wheat |  | Sõmera et al. in prep |
| *Fijivirus* | *Rice black streaked dwarf virus* | Maize | 22nt,21nt; both strands | Li et al. 2017c |
| *Fijivirus* | *Southern rice black-streaked dwarf virus* | Rice | 21nt,22nt; both strands | Xu and Zhou 2017; Lan et al. 2018 |
| *Oryzavirus* | *Rice ragged stunt virus* | Rice |  | Kreuze 2014 |
| *Phytoreovirus* | *Rice dwarf virus* | Rice | 21nt,22nt; both strands | Hong et al. 2015 |
| unassigned | *Cassava frogskin associated virus* | Cassava |  | Carvajal-Yepes et al. 2014 |
| **Rhabdoviridae**  (-)ssRNA |  |  |  |  |
| *Cytorhabdovirus* | *Barley yellow striate mosaic virus* | Wheat | 21nt,22nt; both strands | Yan et al. 2015 |
| *Cytorhabdovirus* unclassified | *Rice stripe mosaic virus* | Rice | 21nt,22nt; both strands | Yang et al. 2017 |
| *Cytorhabdovirus* unclassified | *Tomato yellow mottle-associated virus* | Tomato | 21nt,22nt; both strands | Xu et al. 2017 |
| *Cytorhabdovirus* |  | Common bean |  | Mwaipopo et al. 2018 |
| *Cytorhabdovirus* |  | Water lily |  | Kreuze 2014 |
| *Dichorhavirus* | *Citrus leprosis virus nuclear type* = *Orchid fleck virus* | *Citrus* sp. |  | Roy et al. 2013b,c |
| *Dichorhavirus* | *Citrus leprosis virus nuclear type* | Sweet orange (herbarium) |  | Hartung et al. 2015 |
| *Nucleorhabdovirus* | *Eggplant mottled dwarf virus* | Lisianthus |  | Verdin et al. 2017 |
| *Varicosavirus* | *Lettuce big-vein associated virus* | Lettuce |  | Kreuze 2014 |
| unassigned |  | Kalanchoe |  | Verdin et al. 2017 |
| **Secoviridae**  (+)ssRNA |  |  |  |  |
| *Cheravirus* | *Apple latent spherical virus* | Apple | both strands | Kishigami et al. 2014 |
| *Comovirus* | *Bean pod mottle virus* | Soybean |  | Molina et al. 2012 |
| *Fabavirus* | *Broad bean wilt virus 2* | *N. tabacum* |  | Akinyemi et al. 2016 |
| *Fabavirus* | *Cherry virus F* | Sweet cherry | both strands | Katsiani et al. 2018; Koloniuk et al. 2018 |
| *Fabavirus* | *Grapevine fabavirus* | Grapevine Beta |  | Fan et al. 2017a; Ahmed et al. 2018 |
| *Fabavirus* | *Peach leaf pitting-associated virus* | Peach | 21nt,22nt; both strands | He et al. 2017 |
| *Nepovirus* | *Cycas necrotic stunt virus* | Peony |  | Verdin et al. 2017 |
| *Nepovirus* | *Cycas necrotic stunt virus* | Snapdragon |  | Verdin et al. 2017 |
| *Nepovirus* | *Grapevine chrome mosaic virus* | Grapevine | 21nt,22nt; both strands | Czotter et al. 2018, pers com |
| *Nepovirus* | *Grapevine fanleaf virus* | Grapevine | 21nt,22nt; (+) strand bias | Pantaleo et al. 2010; Turco, Reynard, Pooggin in prep; Chiumenti et al. 2016b |
| *Nepovirus* | *Potato virus B* | Potato | both strands | De Souza et al. 2017; Massart et al. 2018 |
| *Nepovirus* | *Tobacco ringspot virus* | Cleome |  | Verdin et al. 2017 |
| *Nepovirus*  satellite | *Grapevine fanleaf virus* satellite RNA | Grapevine | 21nt,22nt; (+) strand bias | Chiumenti et al. 2016b |
| *Torradovirus* | *Carrot torrado virus 1* | *Torilis arvensis* | both strands | Lotos et al. 2018 |
| *Torradovirus* | *Cassava torrado-like virus* | Cassava |  | Carvajal-Yepes et al. 2014 |
| *Torradovirus* | *Squash chlorotic leaf spot virus* | Zucchini |  | Lecoq et al. 2016 |
| *Torradovirus*-like |  | *N. benthamiana* |  | Kreuze 2014 |
| *Torradovirus*-like |  | Squash |  | Verdin et al. 2017 |
| *Waikavirus* | *Maize chlorotic dwarf virus* | *Pennisetum advena* | 21nt,22nt | Barrero et al. 2017 |
| *Waikavirus* | *Rice tungro spherical virus* | Rice |  | Zarreen et al. 2018 |
| Unassigned | *Black raspberry necrosis virus* | Raspberry | 21nt,22nt; both strands | Susi et al. 2018 |
| Unassigned | *Strawberry mottle virus* | Strawberry | 21nt,22nt | Barrero et al. 2017; Ding et al. 2016 |
| **Solemoviridae**  (+)ssRNA |  |  |  |  |
| *Sobemovirus* | *Cynosurus mottle virus* | Wheat |  | Sõmera et al. in prep |
| *Sobemovirus* | *Southern bean mosaic virus* | Common bean |  | Mwaipopo et al. 2018 |
| *Sobemovirus* | *Solanum nodiflorum mottle virus* | *N. clevelandii* |  | Sõmera et al. in prep |
| *Sobemovirus* | *Velvet tobacco mottle virus* | *N. clevelandii* |  | Sõmera et al. in prep |
| **Tombusviridae**  (+)ssRNA |  |  |  |  |
| *Alphacarmovirus* | *Angelonia flower break virus* | Verbena |  | Verdin et al. 2017 |
| *Alphanecrovirus* | *Corn salad necrosis virus* | *N. benthamiana* |  | Verdin et al. 2018 |
| *Alphanecrovirus* | *Tobacco necrosis virus A* | Potato |  | Bobev et al. 2018 |
| *Betacarmovirus* | *Turnip crinkle virus* | *A. thaliana* | (+) strand bias in AGO2-bound | Harvey et al. 2011 |
| *Betacarmovirus* | *Turnip crinkle virus* | Chickpea | 21nt,22nt; both strands | Ghasemzadeh et al. 2018, pers com |
| *Betacarmovirus* | *Turnip crinkle virus*  mutant deltaCP:GFP | *N. benthamiana* &RNAi mutants | 22nt,21nt,24nt; (+) strand bias | Qin et al. 2017 |
| *Betanecrovirus* | *Beet black scorch virus* | *N. benthamiana* | 22nt,21nt; both strands | Xu et al. 2016 |
| *Betanecrovirus* satellite | *Beet black scorch virus*  satellite RNA | *N. benthamiana* | 22nt,21nt; (+) strand bias | Xu et al. 2016 |
| *Gammacarmovirus* | *Melon necrotic spot virus* | *Cucumis melo* | 21nt,20nt,22nt; both strands | Donaire et al. 2009 |
| *Gammacarmovirus* | *Melon necrotic spot virus* | Melon | 21nt,20nt,22nt; both strands | Herranz et al. 2015 |
| *Machlomovirus* | *Maize chlorotic mottle virus* | Maize | 22nt,21nt; (+) strand bias | Xia et al. 2016 |
| *Panicovirus* | *Ginger chlorotic fleck virus* | Ginger |  | Sõmera et al. in prep |
| *Pelarspovirus* | *Jasmine virus H* | Jasmine |  | Zhuo et al. 2018 |
| *Pelarspovirus* | *Pelargonium line pattern virus* | *N. benthamiana* | 21nt,22nt; both strands | Pérez-Cañamás et al. 2017 |
| *Tombusvirus* | *Cymbidium ringspot virus*  (and its p19 mutant) | *N. benthamiana* | 22nt,21nt; both strands; (+) strand bias | Harris et al. 2015; Donaire et al. 2009; Szittya et al. 2010; Pirovano et al. 2015 |
| *Umbravirus* | *Pea enation mosaic virus 2* | *N. benthamiana* | 21nt,22nt; both strands | Zhou et al. 2017 |
| *Umbravirus* | *Tobacco mottle virus* | Common bean |  | Mwaipopo et al. 2018 |
| *Umbravirus* |  | Common bean |  | Mwaipopo et al. 2018 |
| **Tospoviridae**  (-)ssRNA |  |  |  |  |
| *Orthotospovirus* | *Capsicum chlorosis virus* | Tomato |  | Hagen et al. 2011 |
| *Orthotospovirus* | *Capsicum chlorosis virus* | Zucchini |  | Sun et al. 2018 |
| *Orthotospovirus* | *Chrysanthemum stem necrosis virus* | *N. benthamiana* |  | Pecman et al. 2017 |
| *Orthotospovirus* | *Impatiens necrotic spot virus* | Anemone |  | Verdin et al. 2017 |
| *Orthotospovirus* | *Iris yellow spot virus* | Onion | both strands | Frizzi et al. 2014 |
| *Orthotospovirus* | *Polygonum ringspot virus* | *N. benthamiana* | 21nt,22nt; both strands | Margaria et al. 2016 |
| *Orthotospovirus* | *Polygonum ringspot virus* | Tomato | 22nt,21nt; both strands | Margaria et al. 2016 |
| *Orthotospovirus* | *Tomato spotted wilt virus* | Bell pepper |  | Moyle et al. 2017 |
| *Orthotospovirus* | *Tomato spotted wilt virus* | Buttercup |  | Verdin et al. 2017 |
| *Orthotospovirus* | *Tomato spotted wilt virus* | *Cineraria* sp. |  | Verdin et al. 2017 |
| *Orthotospovirus* | *Tomato spotted wilt virus* | *N. benthamiana* | 21nt,22nt; both strands | Mitter et al. 2013; Margaria et al. 2015 |
| *Orthotospovirus* | *Tomato spotted wilt virus* | Peanut | 21nt,22nt; both strands | Fletcher et al. 2016 |
| *Orthotospovirus* | *Tomato spotted wilt virus* | Tomato | 21nt,22nt; both strands | Mitter et al. 2013; Hagen et al. 2011; Frizzi et al. 2014; Xu et al. 2017 |
| *Orthotospovirus* | *Tomato spotted wilt virus* | Velvet leaf |  | Verdin et al. 2017 |
| *Orthotospovirus* | *Tomato zonate spot virus* | Tomato | 21nt,22nt; both strands | Xu et al. 2017 |
| *Orthotospovirus* |  | Mulberry |  | Meng et al. 2013 |
| **Tymoviridae**  (+)ssRNA |  |  |  |  |
| *Maculavirus* | *Grapevine fleck virus* | Grapevine | 21nt,22nt; (-) strand bias | Barrero et al. 2017; Pantaleo et al. 2010; Miozzi et al. 2013a; Eichmeier et al. 2016; Chiumenti et al. 2016b; Czotter et al. 2018 |
| *Maculavirus* | *Grapevine red globe virus* | Grapevine | 21nt,22nt; both strands; (-) strand bias | Cretazzo and Velasco 2017; Pantaleo et al. 2010, pers com; Czotter et al. 2018, pers com; Fan et al. 2016a; Cretazzo et al. 2017; Massart et al. 2018 |
| *Maculavirus* |  | Fig |  | Chiumenti et al. 2014 |
| *Marafivirus* | *Citrus sudden death-associated virus* | Sweet orange |  | Matsumura et al. 2017 |
| *Marafivirus* | *Grapevine asteroid mosaic associated virus* | Grapevine | 21nt,22nt; (-) strand bias; both strands | Pantaleo et al. 2010, pers com; Czotter et al. 2018, pers com;Vargas-Asencio et al. 2017b |
| *Marafivirus* | *Grapevine rupestris vein feathering virus* | Grapevine | 21nt,22nt; (-) strand bias; both strands | Giampetruzzi et al. 2012; Pantaleo et al. 2010; Saldarelli et al. 2015; Eichmeier et al. 2016; Ma et al. 2017; Czotter et al. 2018, pers com; Massart et al. 2018 |
| *Marafivirus* | *Grapevine Syrah virus 1* | Grapevine | 21nt,22nt; (-) strand bias; both strands | Giampetruzzi et al. 2012; Glasa et al. 2015; Eichmeier et al. 2016; Czotter et al. 2018, pers com; Massart et al. 2018; Ahmed et al. 2018 |
| *Tymovirus* | *Andean potato latent virus* | Potato |  | Kreuze et al. 2013 |
| *Tymovirus* | *Andean potato mild mosaic virus* | Potato |  | Kreuze et al. 2013; Zheng et al. 2017a |
| *Tymovirus* | *Turnip yellow mosaic virus* | *Brassica juncea* | 21nt,22nt,24nt; (-) strand bias | Ma et al. 2015b |
| **Virgaviridae**  (+)ssRNA |  |  |  |  |
| *Furovirus* | *Chinese wheat mosaic virus* | *N. benthamiana* & *rdr6i* mutant | 21nt,22nt; both strands | Andika et al. 2013 |
| *Furovirus* | *Chinese wheat mosaic virus* | Wheat | 21nt,22nt; both strands | Yang et al. 2014 |
| *Hordeivirus* | *Barley stripe mosaic virus* | Barley 700 years old seed |  | Smith et al. 2014 |
| *Hordeivirus* | *Ligustrum mosaic virus*  tentative species | Wild privet | 22nt,21nt; both strands | Reynard, Turco, Pooggin in prep |
| *Pomovirus* | *Potato mop-top virus* | Potato |  | Peña et al. 2016 |
| *Tobamovirus* | *Cucumber green mottle mosaic virus* | Cucumber | 21nt,22nt; (+) strand bias; both strands | Li et al. 2016a |
| *Tobamovirus* | *Cucumber green mottle mosaic virus* | *Lagenaria siceraria* | 21nt,22nt; both strands | Li et al. 2016c |
| *Tobamovirus* | *Paprika mild mottle virus* | Pepper |  | Luria et al. 2018 |
| *Tobamovirus* | *Pepper mild mottle virus* | *N. benthamiana* | 21nt,22nt; (+) strand bias | Donaire et al. 2009 |
| *Tobamovirus* | *Tobacco mild green mosaic virus* | Eggplant |  | Chai et al. 2018 |
| *Tobamovirus* | *Tobacco mild green mosaic virus* | Pumpkin |  | Tang et al. 2017 |
| *Tobamovirus* | *Tobacco mosaic virus* | *A. thaliana* & its *rdr1*, *rdr6* mutants | 21nt,22nt; (+) strand bias; both strands | Qi et al. 2009; Zavallo et al. 2015 |
| *Tobamovirus* | *Tobacco mosaic virus* | *Nicotiana* sp. |  | Pecman et al. 2017 |
| *Tobamovirus* | *Tobacco mosaic virus* | *N. tabacum* | 21nt,22nt; both strands | Konakalla et al. in prep; Wang et al. 2015b; Akinyemi et al. 2016 |
| *Tobamovirus* | *Tobacco mosaic virus* | Pumpkin |  | Tang et al. 2017 |
| *Tobamovirus* | *Tobacco mosaic virus* | Tomato | 21nt,22nt; (+) strand bias | Xu et al. 2017; Che et al. 2018 |
| *Tobamovirus* | *Tomato brown rugose fruit virus* | Tomato | 21nt,22nt; both strands | Luria et al. 2017 |
| *Tobamovirus* | *Tomato mosaic virus* | Tomato | 21nt,22nt; (+) strand bias | Xu et al. 2017; Pecman et al. 2017; Wang et al. 2018c; Che et al. 2018 |
| *Tobamovirus* | *Tomato mottle mosaic virus* | Chickpea | 21nt,22nt; both strands | Pirovano et al. 2015, pers com |
| *Tobamovirus* | *Tomato mottle mosaic virus* | Eggplant |  | Chai et al. 2018 |
| *Tobamovirus* | *Tomato mottle mosaic virus* | Tomato | 21nt,22nt; (+) strand bias | Xu et al. 2017; Li et al. 2013; Padmanabhan et al. 2015a; Che et al. 2018 |
| *Tobamovirus* | *Youcai mosaic virus*  (*Oilseed rape mosaic virus*)  and its p125 mutant | *A. thaliana &* RNAi mutants | 21nt,22nt; both strands; (+) strand bias | Sequin et al. 2014; Zavallo et al. 2015; Malpica-López et al. 2018; Hu et al. 2011 |
| *Tobamovirus* | *Youcai mosaic virus*  and its p125 mutant | *N. benthamiana* | 21nt,22nt; both strands | Malpica-López et al. 2018 |
| *Tobamovirus* | *Youcai mosaic virus* | Bell peper |  | Zhang et al. 2016a |
| *Tobravirus* | *Tobacco rattle virus* | *A. thaliana* | 21nt,22nt,24nt; (+) strand bias | Donaire et al. 2009 |
| *Tobravirus* | *Tobacco rattle virus* | Cleome |  | Verdin et al. 2017 |
| *Tobravirus* | *Tobacco rattle virus* | Peony |  | Verdin et al. 2017 |
| **Unassigned family (+)ssRNA** |  |  |  |  |
| *Cilevirus* | *Citrus leprosis virus cytoplasmic type 2* | *A. thaliana* |  | Ramos-González et al. 2016 |
| *Cilevirus* | *Citrus leprosis virus cytoplasmic type 2* | *Hibiscus rosa-sinensis* |  | Roy et al. 2018a |
| *Cilevirus* | *Citrus leprosis virus cytoplasmic type 2* | Sweet orange | 21nt,22nt | Roy et al. 2013a,b; Ramos-González et al. 2016; Roy et al. 2018a |
| *Cilevirus* | *Citrus leprosis virus cytoplasmic type 2* | Sweet orange (herbarium) |  | Hartung et al. 2015 |
| *Idaeovirus* | *Privet leaf blotch-associated virus* | *Ligustrum japonicum* | 21nt,22nt; both strands | Navarro et al. 2017 |
| *Idaeovirus* | *Raspberry bushy dwarf virus* | Grapevine | 21nt,22nt; both strands | Czotter et al. 2018, pers com |
| *Idaeovirus* | *Raspberry bushy dwarf virus* | Raspberry | 21nt,22nt,24nt; both strands | Barrero et al. 2017; Susi et al. 2018 |
| *Unassigned*  Iflaviridae-like | *Tomato matilda virus* | Tomato |  | Saqib et al. 2015 |
| Satellite virus *Unassigned* | *Grapevine satellite virus* | Grapevine | 21nt,22nt; both strands | Czotter et al. 2018, pers com |
| **Unassigned family**  in Bunyavirales  **(-)ssRNA** |  |  |  |  |
| *Coguvirus* | *Citrus coguvirus* (citrus concave gum‐associated virus) | Blood orange |  | Navarro et al. 2018 |
| *Unassinged* | *Barns Ness serrated wrack bunya/phlebo-like virus 1* | Brown alga *Fucus serratus* | 21nt,20nt; both strands | Waldron et al. 2018 |

**References**

Aguiar ER, Olmo RP, Paro S, Ferreira FV, de Faria IJ, Todjro YM, Lobo FP, Kroon EG, Meignin C, Gatherer D, Imler JL, Marques JT. 2015. Sequence-independent characterization of viruses based on the pattern of viral small RNAs produced by the host. Nucleic Acids Res 43:6191–6206.

Ahmed I, Fan XD, Zhang ZP, Ren F, Hu GJ, Li ZN, Khaskheli MI, Dong YF. 2018. First Report of Grapevine Syrah virus-1 in Grapevines in China. Plant Dis. 102:466. doi.org/10.1094/PDIS-09-17-1358-PDN.

Akinyemi IA, Wang F, Zhou B, Qi S, Wu Q. 2016. Ecogenomic survey of plant viruses infecting Tobacco by Next generation sequencing. Virol J. 13:181.i:10.1093/nar/gkv587.

Alabi OJ, Zheng Y, Jagadeeswaran G, Sunkar R, Naidu RA. 2012. High-throughput sequence analysis of small RNAs in grapevine (Vitis vinifera L.) affected by grapevine leafroll disease. Mol Plant Pathol. 13:1060-76. doi: 10.1111/j.1364-3703.2012.00815.x.

Alejandri-Ramírez ND, Chávez-Hernández EC, Contreras-Guerra JL, Reyes JL, Dinkova TD. 2018. Small RNA differential expression and regulation in Tuxpeño maize embryogenic callus induction and establishment. Plant Physiol Biochem. 122:78-89. doi: 10.1016/j.plaphy.2017.11.013.

Alcalá-Briseño RI, Coşkan S, Londoño MA, Polston JE. 2017. Genome Sequence of Southern tomato virus in Asymptomatic Tomato 'Sweet Hearts'. Genome Announc. 5(7). pii: e01374-16. doi: 10.1128/genomeA.01374-16.

An M, Li R, Gao W, Bi X, Liang Y, Xia Z, Wu Y. 2018. First Report of Peanut mottle virus infecting Peanut in Northeast China. Plant Dis. 8 Aug, First Look. doi.org/10.1094/PDIS-06-18-1037-PDN.

Andika IB, Sun L, Xiang R, Li J, Chen J. 2013. Root-specific role for Nicotiana benthamiana RDR6 in the inhibition of Chinese wheat mosaic virus accumulation at higher temperatures. Mol Plant Microbe Interact. 26:1165-75. doi: 10.1094/MPMI-05-13-0137-R.

Aregger M, Borah BK, Seguin J, Rajeswaran R, Gubaeva EG, Zvereva AS, Windels D, Vazquez F, Blevins T, Farinelli L, Pooggin MM. 2012. Primary and secondary siRNAs in geminivirus-induced gene silencing. PLoS Pathog. 8:e1002941. doi: 10.1371/journal.ppat.1002941.

Baráth D, Jaksa-Czotter N, Molnár J, Varga T, Balássy J, Szabó LK, Kirilla Z, Tusnády GE, Preininger É, Várallyay É. 2018. Small RNA NGS Revealed the Presence of Cherry Virus A and Little Cherry Virus 1 on Apricots in Hungary.Viruses 10(6). pii: E318. doi: 10.3390/v10060318.

Barrero RA, Napier KR, Cunnington J, Liefting L, Keenan S, Frampton RA, Szabo T, Bulman S, Hunter A, Ward L, Whattam M, Bellgard M. 2017. An internet-based bioinformatics toolkit for plant biosecurity diagnosis and surveillance of viruses and viroids. BMC Bioinformatics. 18:26. doi: 10.1186/s12859-016-1428-4.

Becher H, Ma L, Kelly LJ, Kovarik A, Leitch IJ, Leitch AR. 2014. Endogenous pararetrovirus sequences associated with 24 nt small RNAs at the centromeres of Fritillaria imperialis L. (Liliaceae), a species with a giant genome. Plant J. 80:823-33. doi: 10.1111/tpj.12673.

Bester R, Burger JT, Maree HJ. 2017a. Differential expression of miRNAs and associated gene targets in grapevine leafroll-associated virus 3-infected plants. Arch Virol. 162:987-996. doi: 10.1007/s00705-016-3197-9.

Bester, R., Burger, J.T., and Maree, H.J. 2017b. Transcriptome analysis reveals differentially expressed small RNAs and genes associated with grapevine leafroll-associated virus 3 infections. Physiol Mol Plant Pathol. 100:220-236. doi: 10.1016/j.pmpp.2017.10.006.

Bi Y, Artola K, Kurokura T, Hytonen T, Valkonen JPT. 2012a. First report of raspberry leaf blotch virus in raspberries in Finland. Plant Disease 96:1231-1232. doi: 10.1094/PDIS-04-12-0368-PDN

Bi Y, Tugume AK, Valkonen JP. 2012b. Small-RNA deep sequencing reveals Arctium tomentosum as a natural host of Alstroemeria virus X and a new putative Emaravirus. PLoS One 7:e42758. doi: 10.1371/journal.pone.0042758.

Blevins T, Rajeswaran R, Aregger M, Borah BK, Schepetilnikov M, Baerlocher L, Farinelli L, Meins F Jr, Hohn T, Pooggin MM. 2011. Massive production of small RNAs from a non-coding region of Cauliflower mosaic virus in plant defense and viral counter-defense. Nucleic Acids Res. 39:5003-14. doi: 10.1093/nar/gkr119.

Blouin AG, Chooi KM, Warren B, Napier KR, Barrero RA, MacDiarmid RM. 2018a. Grapevine virus I, a putative new vitivirus detected in co-infection with grapevine virus G in New Zealand. Arch Virol. 163:1371-1374. doi: 10.1007/s00705-018-3738-5.

Blouin AG, Keenan S, Napier KR, Barrero RA, MacDiarmid RM. 2018b. Identification of a novel vitivirus from grapevines in New Zealand. Arch Virol. 163:281-284. doi: 10.1007/s00705-017-3581-0.

Bobev SG, De Jonghe K, Maes M. 2018. First Report of Tobacco necrosis virus Causing the ABC Disease on Potato Tubers in Bulgaria. Plant Dis. 102:829. doi.org/10.1094/PDIS-08-17-1188-PDN.

Candresse T, Filloux D, Muhire B, Julian C, Galzi S, Fort G, Bernardo P, Daugrois JH, Fernandez E, Martin DP, Varsani A, Roumagnac P. 2014. Appearances can be deceptive: revealing a hidden viral infection with deep sequencing in a plant quarantine context. PLoS One 9:e102945. doi: 10.1371/journal.pone.0102945.

Cao M, Lan P, Li F, Abad J, Zhou C, Li R. 2017a. Genome characterization of sweet potato symptomless virus 1: a mastrevirus with an unusual nonanucleotide sequence. Arch Virol. 162:2881-2884. doi: 10.1007/s00705-017-3396-z.

Cao MJ, Yu YQ, Tian X, Yang FY, Li RH, Zhou CY. 2017b. First Report of Citrus leaf blotch virus in Lemon in China. Plant Dis. 101:1561. doi.org/10.1094/PDIS-10-16-1500-PDN.

Carvajal-Yepes M, Olaya C, Lozano I, Cuervo M, Castaño M, Cuellar WJ. 2014. Unraveling complex viral infections in cassava (Manihot esculenta Crantz) from Colombia. Virus Res. 186:76-86. doi: 10.1016/j.virusres.2013.12.011.

Chai A, Chen LD, Shi Y, Xie X, Li B, Zhang X, Shi J. 2018. First Report of a Mixed Infection of Tomato mottle mosaic virus and Tobacco mild green mosaic virus on Eggplants in China. Plant Dis. Accepted. doi.org/10.1094/PDIS-04-18-0686-PDN.

Che HY, Luo DQ, Cao XR. 2018. First Report of Tomato Mottle Mosaic Virus in Tomato Crops in China. Plant Dis. 13 Aug. doi.org/10.1094/PDIS-03-18-0538-PDN.

Chen M, Sun L, Wu H, Chen J, Ma Y, Zhang X, Du L, Cheng S, Zhang B, Ye X, Pang J, Zhang X, Li L, Andika IB, Chen J, Xu H. 2014. Durable field resistance to wheat yellow mosaic virus in transgenic wheat containing the antisense virus polymerase gene. Plant Biotechnol J. 12:447-56. doi: 10.1111/pbi.12151.

Chen B, Bernards M, Wang A. 2015a. Complete Genome Sequence of a Bell Pepper Endornavirus Isolate from Canada. Genome Announc. 3(4). pii: e00905-15. doi: 10.1128/genomeA.00905-15

Chen S, Huang Q, Wu L, Qian Y. 2015b. Identification and characterization of a maize-associated mastrevirus in China by deep sequencing small RNA populations. Virol J. 12:156. doi: 10.1186/s12985-015-0384-3.

Chen S, Jiang G, Wu J, Liu Y, Qian Y, Zhou X. 2016. Characterization of a Novel Polerovirus Infecting Maize in China. Viruses 8(5). pii: E120. doi: 10.3390/v8050120.

Chiquito-Almanza E, Caballero-Pérez J, Guevara-Olvera L, Acosta-García G, Pérez-Pérez MCI, Acosta-Gallegos JA, Anaya-López JL. 2018. First Report of Cowpea mild mottle virus Infecting Cultivated and Wild Phaseolus in the Central-Western Region of Mexico. Plant Dis. 102:1047. doi.org/10.1094/PDIS-07-17-0990-PDN.

Chiumenti M, Torchetti EM, Di Serio F, Minafra A. 2014. Identification and characterization of a viroid resembling apple dimple fruit viroid in fig (Ficus carica L.) by next generation sequencing of small RNAs. Virus Res. 188:54-9. doi: 10.1016/j.virusres.2014.03.026.

Chiumenti M, Giampetruzzi A, Morelli M, Savino VN, Martelli GP, La Notte P, Palmisano F, Saldarelli P. 2016a. Detection and molecular characterization of a Grapevine Roditis leaf discoloration-associated virus (GRLDaV) variant in an autochthonous grape from Apulia (Italy). Virus Genes 52:428-31. doi: 10.1007/s11262-016-1305-2

Chiumenti M, Mohorianu I, Roseti V, Saldarelli P, Dalmay T, Minafra A. 2016b. High-throughput-sequencing-based identification of a grapevine fanleaf virus satellite RNA in Vitis vinifera. Arch Virol. 161(5):1401-3. doi: 10.1007/s00705-016-2776-0.

Chiumenti M, Morelli M, De Stradis A, Elbeaino T, Stavolone L, Minafra A. 2016c. Unusual genomic features of a badnavirus infecting mulberry. J Gen Virol. 97:3073-3087. doi: 10.1099/jgv.0.000600.

Creasey KM, Zhai J, Borges F, Van Ex F, Regulski M, Meyers BC, Martienssen RA. 2014. miRNAs trigger widespread epigenetically activated siRNAs from transposons in Arabidopsis. Nature 508:411-5. doi: 10.1038/nature13069.

Cretazzo E, Padilla CV, Velasco L. 2017. First Report of Grapevine Red Globe virus in grapevine in Spain. Plant Dis. 101:264. doi.org/10.1094/PDIS-06-16-0932-PDN.

Cretazzo E, Velasco L. 2017. High‐throughput sequencing allowed the completion of the genome of grapevine Red Globe virus and revealed recurring co‐infection with other tymoviruses in grapevine. Plant Pathol. 66:1202-1213. doi.org/10.1111/ppa.12669.

Cuellar WJ, Cruzado RK, Fuentes S, Untiveros M, Soto M, Kreuze JF. 2011. Sequence characterization of a Peruvian isolate of Sweet potato chlorotic stunt virus: further variability and a model for p22 acquisition. Virus Res. 157:111-5. doi: 10.1016/j.virusres.2011.01.010.

Cuellar WJ, Galvez M, Fuentes S, Tugume J, Kreuze J. 2015. Synergistic interactions of begomoviruses with Sweet potato chlorotic stunt virus (genus Crinivirus) in sweet potato (Ipomoea batatas L.). Mol Plant Pathol. 16:459-71. doi: 10.1111/mpp.12200.

Czotter N, Molnar J, Szabó E, Demian E, Kontra L, Baksa I, Szittya G, Kocsis L, Deak T, Bisztray G, Tusnady GE, Burgyan J, Varallyay E. 2018. NGS of Virus-Derived Small RNAs as a Diagnostic Method Used to Determine Viromes of Hungarian Vineyards. Front Microbiol. 9:122. doi: 10.3389/fmicb.2018.00122.

Da Fonseca GC, de Oliveira LFV, de Morais GL, Abdelnor RV, Nepomuceno AL, Waterhouse PM, Farinelli L, Margis R. 2016. Unusual RNA plant virus integration in the soybean genome leads to the production of small RNAs. Plant Sci. 246:62-69. doi: 10.1016/j.plantsci.2016.01.011.

De Haro LA, Dumón AD, Mattio MF, Argüello Caro EB, Llauger G, Zavallo D, Blanc H, Mongelli VC, Truol G, Saleh MC, Asurmendi S, Del Vas M. 2017. Mal de Río Cuarto Virus Infection Triggers the Production of Distinctive Viral-Derived siRNA Profiles in Wheat and Its Planthopper Vector. Front Plant Sci. 8:766. doi: 10.3389/fpls.2017.00766.

De Souza J, Fuentes S, Savenkov EI, Cuellar W, Kreuze JF. 2013. The complete nucleotide sequence of sweet potato C6 virus: a carlavirus lacking a cysteine-rich protein. Arch Virol. 158:1393-6. doi: 10.1007/s00705-013-1614-x.

De Souza J, Müller G, Perez W, Cuellar W, Kreuze J. 2017. Complete sequence and variability of a new subgroup B nepovirus infecting potato in central Peru. Arch Virol. 162:885-889. doi: 10.1007/s00705-016-3147-6.

Del Toro FJ, Donaire L, Aguilar E, Chung BN, Tenllado F, Canto T. 2017. Potato Virus Y HCPro Suppression of Antiviral Silencing in Nicotiana benthamiana Plants Correlates with Its Ability To Bind In Vivo to 21- and 22-Nucleotide Small RNAs of Viral Sequence. J Virol. 91:e00367-17. doi: 10.1128/JVI.00367-17.

Desbiez C, Schoeny A, Maisonneuve B, Berthier K, Bornard I, Chandeysson C, Fabre F, Girardot G, Gognalons P, Lecoq H, Lot H, Millot P, Nozeran K, Simon V, Tepfer M, Verdin E, Wipf‐Scheibel C, Moury B. 2017a. Molecular and biological characterization of two potyviruses infecting lettuce in southeastern France. Plant Pathol. 66: 970-979. doi: 10.1111/ppa.12651.

Desbiez C, Wipf-Scheibel C, Millot P, Verdin E, Dafalla G, Lecoq H. 2017b. New species in the papaya ringspot virus cluster: Insights into the evolution of the PRSV lineage. Virus Res. 241:88-94. doi: 10.1016/j.virusres.2017.06.022.

Ding X, Li Y, Hernández-Sebastià C, Abbasi PA, Fisher P, Celetti MJ, Wang A. 2016. First Report of Strawberry crinivirus 4 on Strawberry in Canada. Plant Dis. 100:1254. doi.org/10.1094/PDIS-01-16-0009-PDN.

Ding X, Chen D, Wang A, Zhang J, Wu Z. 2017. First Report of Strawberry pallidosis-associated virus on Strawberry in China. Plant Dis. 101: 2154. doi.org/10.1094/PDIS-05-17-0711-PDN.

Donaire L, Wang Y, Gonzalez-Ibeas D, Mayer KF, Aranda MA, Llave C. 2009. Deep-sequencing of plant viral small RNAs reveals effective and widespread targeting of viral genomes. Virology 392:203-14. doi: 10.1016/j.virol.2009.07.005.

Eichmeier A, Komínková M, Komínek P, Baránek M. 2016. Comprehensive Virus Detection Using Next Generation Sequencing in Grapevine Vascular Tissues of Plants Obtained from the Wine Regions of Bohemia and Moravia (Czech Republic). PLoS One 11:e0167966. doi: 10.1371/journal.pone.0167966.

Fan XD, Dong YF, Zhang ZP, Ren F, Hu GJ, Li ZN, Zhou J. 2016a. First Report of Grapevine red globe virus (GRGV) in Grapevines in China. Plant Dis. 100:2340. doi.org/10.1094/PDIS-05-16-0701-PDN.

Fan X, Hong N, Zhang Z, Yang Z, Ren F, Hu G, Li Z, Zhou J, Dong Y, Wang G. 2016b. Identification of a divergent variant of grapevine berry inner necrosis virus in grapevines showing chlorotic mottling and ring spot symptoms. Arch Virol. 161:2025-7. doi: 10.1007/s00705-016-2856-1.

Fan XD, Zhang ZP, Ren F, Hu GJ, Li ZN, Dong YF. 2017a. First Report of Grapevine fabavirus in Grapevines in China. Plant Dis. 101:847. doi.org/10.1094/PDIS-10-16-1479-PDN.

Fan XD, Zhang ZP, Ren F, Hu GJ, Li ZN, Dong YF. 2017b. First Report of Grapevine geminivirus A from Grapevines in China. Plant Dis. 101:1333. doi.org/10.1094/PDIS-01-17-0106-PDN.

Fang YY, Smith NA, Zhao JH, Lee JR, Guo HS, Wang MB. 2015. Cloning and profiling of small RNAs from cucumber mosaic virus satellite RNA. Methods Mol Biol. 1236:99-109. doi: 10.1007/978-1-4939-1743-3_9.

Filloux D, Murrell S, Koohapitagtam M, Golden M, Julian C, Galzi S, Uzest M, Rodier-Goud M, D'Hont A, Vernerey MS, Wilkin P, Peterschmitt M, Winter S, Murrell B, Martin DP, Roumagnac P. 2015. The genomes of many yam species contain transcriptionally active endogenous geminiviral sequences that may be functionally expressed. Virus Evol. 1(1):vev002. DOI: 10.1093/ve/vev002.

Fiore N, Fernández C, Quiroga N, Pino AM, Rivera L, Sagredo K, Zamorano A. 2018. First Report of Little cherry virus 1 in Chile. Plant Dis. 102:689. doi.org/10.1094/PDIS-09-17-1407-PDN.

Fletcher SJ, Shrestha A, Peters JR, Carroll BJ, Srinivasan R, Pappu HR, Mitter N. 2016. The Tomato Spotted Wilt Virus Genome Is Processed Differentially in its Plant Host Arachis hypogaea and its Thrips Vector Frankliniella fusca. Front Plant Sci. 7:1349. doi: 10.3389/fpls.2016.01349.

Folimonova SY, Harper SJ, Leonard MT, Triplett EW, Shilts T. 2014. Superinfection exclusion by Citrus tristeza virus does not correlate with the production of viral small RNAs. Virology 468-470:462-471. doi: 10.1016/j.virol.2014.08.031.

Frizzi A, Zhang Y, Kao J, Hagen C, Huang S. 2014. Small RNA profiles from virus-infected fresh market vegetables. J Agric Food Chem. 62:12067-74. doi: 10.1021/jf503756v.

Fuentes S, Heider B, Tasso RC, Romero E, Zum Felde T, Kreuze JF. 2012. Complete genome sequence of a potyvirus infecting yam beans (Pachyrhizus spp.) in Peru. Arch Virol. 157:773-6. doi: 10.1007/s00705-011-1214-6.

Fuentes A, Carlos N, Ruiz Y, Callard D, Sánchez Y, Ochagavía ME, Seguin J, Malpica-López N, Hohn T, Lecca MR, Pérez R, Doreste V, Rehrauer H, Farinelli L, Pujol M, Pooggin MM. 2016. Field Trial and Molecular Characterization of RNAi-Transgenic Tomato Plants That Exhibit Resistance to Tomato Yellow Leaf Curl Geminivirus. Mol Plant Microbe Interact. 29:197-209. doi: 10.1094/MPMI-08-15-0181-R.

Garcia-Ruiz H, Takeda A, Chapman EJ, Sullivan CM, Fahlgren N, Brempelis KJ, Carrington JC. 2010. Arabidopsis RNA-dependent RNA polymerases and dicer-like proteins in antiviral defense and small interfering RNA biogenesis during Turnip Mosaic Virus infection. Plant Cell 22:481-96. doi: 10.1105/tpc.109.073056.

Garcia-Ruiz H, Carbonell A, Hoyer JS, Fahlgren N, Gilbert KB, Takeda A, Giampetruzzi A, Garcia Ruiz MT, McGinn MG, Lowery N, Martinez Baladejo MT, Carrington JC. 2015. Roles and programming of Arabidopsis ARGONAUTE proteins during Turnip mosaic virus infection. PLoS Pathog. 11:e1004755. doi: 10.1371/journal.ppat.1004755.

Geering AD, Maumus F, Copetti D, Choisne N, Zwickl DJ, Zytnicki M, McTaggart AR, Scalabrin S, Vezzulli S, Wing RA, Quesneville H, Teycheney PY. 2014. Endogenous florendoviruses are major components of plant genomes and hallmarks of virus evolution. Nat Commun. 5:5269. doi: 10.1038/ncomms6269.

Ghasemzadeh A, Ter Haar MM, Shams-Bakhsh M, Pirovano W, Pantaleo V. 2018. Shannon Entropy to Evaluate Substitution Rate Variation Among Viral Nucleotide Positions in Datasets of Viral siRNAs. Methods Mol Biol. 1746:187-195. doi: 10.1007/978-1-4939-7683-6_15.

Giampetruzzi A, Roumi V, Roberto R, Malossini U, Yoshikawa N, La Notte P, Terlizzi F, Credi R, Saldarelli P. 2012. A new grapevine virus discovered by deep sequencing of virus- and viroid-derived small RNAs in Cv Pinot gris. Virus Res. 163:262-8. doi: 10.1016/j.virusres.2011.10.010.

Glasa M, Predajňa L, Komínek P, Nagyová A, Candresse T, Olmos A. 2014. Molecular characterization of divergent grapevine Pinot gris virus isolates and their detection in Slovak and Czech grapevines. Arch Virol. 159:2103-7. doi: 10.1007/s00705-014-2031-5.

Glasa M, Predajňa L, Šoltys K, Sabanadzovic S, Olmos A. 2015. Detection and molecular characterisation of Grapevine Syrah virus-1 isolates from Central Europe. Virus Genes 51:112-21. doi: 10.1007/s11262-015-1201-1.

Hagen C, Frizzi A, Kao J, Jia L, Huang M, Zhang Y, Huang S. 2011. Using small RNA sequences to diagnose, sequence, and investigate the infectivity characteristics of vegetable-infecting viruses. Arch Virol. 156:1209-16. doi: 10.1007/s00705-011-0979-y.

Han T, Yang CX, Fu JJ, Hou QS, Gang S, Chen S, Shen P, Cao MJ. First Report of Ligustrum Virus A on Syringa reticulata var. mandshurica (Oleaceae) with a New Yellow Vein and Malformed Needle-Shaped Leaf Disease in China. Plant Dis. 13 Aug. doi.org/10.1094/PDIS-04-18-0610-PDN.

Hartung JS, Roy A, Fu S, Shao J, Schneider WL, Brlansky RH. 2015. History and Diversity of Citrus leprosis virus Recorded in Herbarium Specimens. Phytopathology 105:1277-84. doi: 10.1094/PHYTO-03-15-0064-R.

Harvey JJ, Lewsey MG, Patel K, Westwood J, Heimstädt S, Carr JP, Baulcombe DC. 2011. An antiviral defense role of AGO2 in plants. PLoS One 6:e14639. doi: 10.1371/journal.pone.0014639.

He Y, Yang Z, Hong N, Wang G, Ning G, Xu W. 2015. Deep sequencing reveals a novel closterovirus associated with wild rose leaf rosette disease. Mol Plant Pathol. 16:449-58. doi: 10.1111/mpp.12202.

He Y, Cai L, Zhou L, Yang Z, Hong N, Wang G, Li S, Xu W. 2017. Deep sequencing reveals the first fabavirus infecting peach. Sci Rep. 7:11329. doi: 10.1038/s41598-017-11743-7.

Herranz MC, Navarro JA, Sommen E, Pallas V. 2015. Comparative analysis among the small RNA populations of source, sink and conductive tissues in two different plant-virus pathosystems. BMC Genomics 16:117. doi: 10.1186/s12864-015-1327-5.

Ho T, Wang L, Huang L, Li Z, Pallett DW, Dalmay T, Ohshima K, Walsh JA, Wang H. 2010. Nucleotide bias of DCL and AGO in plant anti-virus gene silencing. Protein Cell 1:847-58. doi: 10.1007/s13238-010-0100-4.

Hong W, Qian D, Sun R, Jiang L, Wang Y, Wei C, Zhang Z, Li Y. 2015. OsRDR6 plays role in host defense against double-stranded RNA virus, Rice Dwarf Phytoreovirus. Sci Rep. 5:11324. doi: 10.1038/srep11324.

Howard S, Qiu W. 2017. Viral small RNAs reveal the genomic variations of three grapevine vein clearing virus quasispecies populations. Virus Res. 229:24-27. doi: 10.1016/j.virusres.2016.12.012.

Hu Q, Hollunder J, Niehl A, Kørner CJ, Gereige D, Windels D, Arnold A, Kuiper M, Vazquez F, Pooggin M, Heinlein M. 2011. Specific impact of tobamovirus infection on the Arabidopsis small RNA profile. PLoS One 6:e19549. doi: 10.1371/journal.pone.0019549.

Hu W, Li Z, Wang X, Liu W, Huang C, Miao W, Cui H. 2018. Complete genomic sequence of a novel macluravirus, alpinia oxyphylla mosaic virus (AloMV), identified in Alpinia oxyphylla. Arch Virol. May 21. doi: 10.1007/s00705-018-3879-6.

Hwang YT, Kalischuk M, Fusaro AF, Waterhouse PM, Kawchuk L. 2013. Small RNA sequencing of Potato leafroll virus-infected plants reveals an additional subgenomic RNA encoding a sequence-specific RNA-binding protein. Virology 438:61-9. doi: 10.1016/j.virol.2012.12.012.

Jakse J, Radisek S, Pokorn T, Matousek J, Javornik B. 2015. Deep‐sequencing revealed Citrus bark cracking viroid (CBCVd) as a highly aggressive pathogen on hop. Plant Pathol. 64:831-842. doi: 10.1111/ppa.12325.

Jiang L, Qian D, Zheng H, Meng LY, Chen J, Le WJ, Zhou T, Zhou YJ, Wei CH, Li Y. 2012. RNA-dependent RNA polymerase 6 of rice (Oryza sativa) plays role in host defense against negative-strand RNA virus, Rice stripe virus. Virus Res. 163:512-9. doi: 10.1016/j.virusres.2011.11.016.

Jimenez J, Carvajal-Yepes M, Leiva AM, Cruz M, Romero LE, Bolaños CA, Lozano I, Cuellar WJ. 2018. Complete Genome Sequence of Rice hoja blanca tenuivirus Isolated from a Susceptible Rice Cultivar in Colombia. Genome Announc. 6(7). pii: e01490-17. doi: 10.1128/genomeA.01490-17.

Kaldis A, Berbati M, Melita O, Reppa C, Holeva M, Otten P, Voloudakis A. 2018. Exogenously applied dsRNA molecules deriving from the Zucchini yellow mosaic virus (ZYMV) genome move systemically and protect cucurbits against ZYMV. Mol Plant Pathol. 19:883-895. doi: 10.1111/mpp.12572.

Kalischuk ML, Fusaro AF, Waterhouse PM, Pappu HR, Kawchuk LM. 2013. Complete genomic sequence of a Rubus yellow net virus isolate and detection of genome-wide pararetrovirus-derived small RNAs. Virus Res. 178:306-13. doi: 10.1016/j.virusres.2013.09.026.

Kamran A, Lotos L, Amer MA, Al-Saleh MA, Alshahwan IM, Shakeel MT, Ahmad MH, Umar M, Katis NI. 2018. Characterization of Pepper leafroll chlorosis virus, a New Polerovirus Causing Yellowing Disease of Bell Pepper in Saudi Arabia. Plant Dis. 102:318-326. doi.org/10.1094/PDIS-03-17-0418-RE.

Karthikeyan C, Patil BL, Borah BK, Resmi TR, Turco S, Pooggin MM, Hohn T, Veluthambi K. 2016. Emergence of a Latent Indian Cassava Mosaic Virus from Cassava Which Recovered from Infection by a Non-Persistent Sri Lankan Cassava Mosaic Virus. Viruses 8(10). pii: E264. doi: 10.3390/v8100264

Kashif M, Pietilä S, Artola K, Jones RAC, Tugume AK, Mäkinen V, Valkonen JPT. 2012. Detection of viruses in sweetpotato from Honduras and Guatemala augmented by deep-sequencing of small-RNAs. Plant Dis. 96:1430-1437. doi.org/10.1094/PDIS-03-12-0268-RE.

Katsiani A, Maliogka VI, Katis N, Svanella-Dumas L, Olmos A, Ruiz-García AB, Marais A, Faure C, Theil S, Lotos L, Candresse T. 2018. High-Throughput Sequencing Reveals Further Diversity of Little Cherry Virus 1 with Implications for Diagnostics. Viruses 10(7). pii: E385. doi: 10.3390/v10070385.

Kazmi SA, Yang Z, Hong N, Wang G, Wang Y. 2015. Characterization by Small RNA Sequencing of Taro Bacilliform CH Virus (TaBCHV), a Novel Badnavirus. PLoS One 10:e0134147. doi: 10.1371/journal.pone.0134147.

Koloniuk I, Sarkisova T, Petrzik K, Lenz O, Přibylová J, Fránová J, Špak J, Lotos L, Beta C, Katsiani A, Candresse T, Maliogka VI. 2018. Variability Studies of Two Prunus-Infecting Fabaviruses with the Aid of High-Throughput Sequencing. Viruses 10(4). pii: E204. doi: 10.3390/v10040204.

Kishigami R, Yamagishi N, Ito T, Yoshikawa N. 2014. Detection of apple latent spherical virus in seeds and seedlings from infected apple trees by reverse transcription quantitative PCR and deep sequencing: evidence for lack of transmission of the virus to most progeny seedlings. J Gen Plant Pathol. 80:490-498. doi: 10.1007/s10327-014-0541-3.

Kreuze J. 2014. siRNA Deep Sequencing and Assembly: Piecing Together Viral Infections.... In M.L. Gullino and P.J.M. Bonants (eds.), Detection and Diagnostics of Plant Pathogens, Plant Pathology in the 21st Century 5, Springer Science+Business Media Dordrecht 2014. pp. 21-38. doi: https://link.springer.com/chapter/10.1007/978-94-017-9020-8_2

Kreuze J, Koenig R, De Souza J, Vetten HJ, Muller G, Flores B, Ziebell H, Cuellar W. 2013. The complete genome sequences of a Peruvian and a Colombian isolate of Andean potato latent virus and partial sequences of further isolates suggest the existence of two distinct potato-infecting tymovirus species. Virus Res. 173:431-5. doi: 10.1016/j.virusres.2013.01.014.

Kreuze JF, Perez A, Untiveros M, Quispe D, Fuentes S, Barker I, Simon R. 2009. Complete viral genome sequence and discovery of novel viruses by deep sequencing of small RNAs: a generic method for diagnosis, discovery and sequencing of viruses. Virology 388:1-7. doi: 10.1016/j.virol.2009.03.024.

Kumar S, Subbarao BL, Hallan V. 2017. Molecular characterization of emaraviruses associated with Pigeonpea sterility mosaic disease. Sci Rep. 7(1):11831. doi: 10.1038/s41598-017-11958-8.

Kung YJ, Lin PC, Yeh SD, Hong SF, Chua NH, Liu LY, Lin CP, Huang YH, Wu HW, Chen CC, Lin SS. 2014. Genetic analyses of the FRNK motif function of Turnip mosaic virus uncover multiple and potentially interactive pathways of cross-protection. Mol Plant Microbe Interact. 27:944-55. doi: 10.1094/MPMI-04-14-0116-R.

Kuria P, Ilyas M, Ateka E, Miano D, Onguso J, Carrington JC, Taylor NJ. 2017. Differential response of cassava genotypes to infection by cassava mosaic geminiviruses. Virus Res. 227:69-81. doi: 10.1016/j.virusres.2016.09.022.

Kutnjak D, Silvestre R, Cuellar W, Perez W, Müller G, Ravnikar M, Kreuze J. 2014. Complete genome sequences of new divergent potato virus X isolates and discrimination between strains in a mixed infection using small RNAs sequencing approach. Virus Res. 191:45-50. doi: 10.1016/j.virusres.2014.07.012.

Kutnjak D, Rupar M, Gutierrez-Aguirre I, Curk T, Kreuze JF, Ravnikar M. 2015. Deep sequencing of virus-derived small interfering RNAs and RNA from viral particles shows highly similar mutational landscapes of a plant virus population. J Virol. 89:4760-9. doi: 10.1128/JVI.03685-14.

Lan Y, Li Y, E Z, Sun F, Du L, Xu Q, Zhou T, Zhou Y, Fan Y. 2018. Identification of virus-derived siRNAs and their targets in RBSDV-infected rice by deep sequencing. J Basic Microbiol. 58:227-237. doi: 10.1002/jobm.201700325.

Lecoq H, Verdin E, Tepfer M, Wipf-Scheibel C, Millot P, Dafalla G, Desbiez C. 2016. Characterization and occurrence of squash chlorotic leaf spot virus, a tentative new torradovirus infecting cucurbits in Sudan. Arch Virol. 161:1651-5. doi: 10.1007/s00705-016-2797-8.

Leibman D, Wolf D, Saharan V, Zelcer A, Arazi T, Yoel S, Gaba V, Gal-On A. 2011. A high level of transgenic viral small RNA is associated with broad potyvirus resistance in cucurbits. Mol Plant Microbe Interact. 24:1220-38. doi: 10.1094/MPMI-05-11-0128.

Leibman D, Prakash S, Wolf D, Zelcer A, Anfoka G, Haviv S, Brumin M, Gaba V, Arazi T, Lapidot M, Gal-On A. 2015. Immunity to tomato yellow leaf curl virus in transgenic tomato is associated with accumulation of transgene small RNA. Arch Virol. 160:2727-39. doi: 10.1007/s00705-015-2551-7.

Li R, Gao S, Hernandez AG, Wechter WP, Fei Z, Ling KS. 2012. Deep sequencing of small RNAs in tomato for virus and viroid identification and strain differentiation. PLoS One 7:e37127. doi: 10.1371/journal.pone.0037127.

Li R, Gao S, Fei Z, Ling KS. 2013. Complete genome sequence of a new tobamovirus naturally infecting tomatoes in Mexico. Genome Announc. 1(5). pii: e00794-13. doi: 10.1128/genomeA.00794-13.

Li Y, Deng C, Shang Q, Zhao X, Liu X, Zhou Q. 2016a. Characterization of siRNAs derived from cucumber green mottle mosaic virus in infected cucumber plants. Arch Virol. 161:455-8. doi: 10.1007/s00705-015-2687-5.

Li Y, Deng C, Shang Q, Zhao X, Liu X, Zhou Q. 2016b. The first complete genome sequence of iris severe mosaic virus. Arch Virol. 161:1069-72. doi: 10.1007/s00705-015-2743-1.

Li J, Zheng H, Zhang C, Han K, Wang S, Peng J, Lu Y, Zhao J, Xu P, Wu X, Li G, Chen J, Yan F. 2016c. Different Virus-Derived siRNAs Profiles between Leaves and Fruits in Cucumber Green Mottle Mosaic Virus-Infected Lagenaria siceraria Plants. Front Microbiol. 7:1797.

Li L, Andika IB, Xu Y, Zhang Y, Xin X, Hu L, Sun Z, Hong G, Chen Y, Yan F, Yang J, Li J, Chen J. 2017a. Differential Characteristics of Viral siRNAs between Leaves and Roots of Wheat Plants Naturally Infected with Wheat Yellow Mosaic Virus, a Soil-Borne Virus. Front Microbiol. 8:1802. doi: 10.3389/fmicb.2017.01802.

Li Y, Deng C, Bian Y, Zhao X, Zhou Q. 2017b. Characterization of apple stem grooving virus and apple chlorotic leaf spot virus identified in a crab apple tree. Arch Virol. 162(4):1093-1097. doi: 10.1007/s00705-016-3183-2.

Li M, Li Y, Xia Z, Di D, Zhang A, Miao H, Zhou T, Fan Z. 2017c. Characterization of small interfering RNAs derived from Rice black streaked dwarf virus in infected maize plants by deep sequencing. Virus Res. 228:66-74. doi: 10.1016/j.virusres.2016.11.001.

Li Y, Jia A, Qiao Y, Xiang J, Zhang Y, Wang W. 2018. Virome analysis of lily plants reveals a new potyvirus. Arch Virol. 163:1079-1082. doi: 10.1007/s00705-017-3690-9.

Liang P, Navarro B, Zhang Z, Wang H, Lu M, Xiao H, Wu Q, Zhou X, Di Serio F, Li S. 2015. Identification and characterization of a novel geminivirus with a monopartite genome infecting apple trees. J Gen Virol. 96:2411-20. doi: 10.1099/vir.0.000173.

Licciardello G, Scuderi G, Ferraro R, Giampetruzzi A, Russo M, Lombardo A, Raspagliesi D, Bar-Joseph M, Catara A. 2015. Deep sequencing and analysis of small RNAs in sweet orange grafted on sour orange infected with two citrus tristeza virus isolates prevalent in Sicily. Arch Virol. 160:2583-9. doi: 10.1007/s00705-015-2516-x.

Lin KY, Cheng CP, Chang BC, Wang WC, Huang YW, Lee YS, Huang HD, Hsu YH, Lin NS. 2010. Global analyses of small interfering RNAs derived from Bamboo mosaic virus and its associated satellite RNAs in different plants. PLoS One 5:e11928. doi: 10.1371/journal.pone.0011928.

Lin W, Yan W, Yang W, Yu C, Chen H, Zhang W, Wu Z, Yang L, Xie L. 2017. Characterisation of siRNAs derived from new isolates of bamboo mosaic virus and their associated satellites in infected ma bamboo (Dendrocalamus latiflorus). Arch Virol. 162:505-510. doi: 10.1007/s00705-016-3092-4.

Liu M, Liu X, Li X, Zhang D, Dai L, Tang Q. 2016a. Complete genome sequence of a Chinese isolate of pepper vein yellows virus and evolutionary analysis based on the CP, MP and RdRp coding regions. Arch Virol. 161:677-83. doi: 10.1007/s00705-015-2691-9.

Liu J, Zhang X, Yang Y, Hong N, Wang G, Wang A, Wang L. 2016b. Characterization of virus-derived small interfering RNAs in Apple stem grooving virus-infected in vitro-cultured Pyrus pyrifolia shoot tips in response to high temperature treatment. Virol J. 13:166.

Liu JY, Fan HY, Wang Y, Zhang YL, Li DW, Yu JL, Han CG. 2017. Characterization of microRNAs of Beta macrocarpa and their responses to Beet necrotic yellow vein virus infection. PLoS One 12:e0186500. doi: 10.1371/journal.pone.0186500.

Liu W, Xin M, Cao M, Qin M, Liu H, Zhao S, Wang X. 2018. Identification, characterization and full-length sequence analysis of a novel endornavirus in common sunflower (Helianthus annuus L.). Journal of Integrative Agriculture17:60345-7. doi: 10.1016/S2095-3119(18)61963-X.

Loconsole G, Onelge N, Potere O, Giampetruzzi A, Bozan O, Satar S, De Stradis A, Savino V, Yokomi RK, Saponari M. 2012a. Identification and characterization of citrus yellow vein clearing virus, a putative new member of the genus Mandarivirus. Phytopathology 102:1168-75. doi: 10.1094/PHYTO-06-12-0140-R.

Loconsole G, Saldarelli P, Doddapaneni H, Savino V, Martelli GP, Saponari M. 2012b. Identification of a single-stranded DNA virus associated with citrus chlorotic dwarf disease, a new member in the family Geminiviridae. Virology 432:162-72. doi: 10.1016/j.virol.2012.06.005.

Lotos L, Olmos A, Orfanidou C, Efthimiou K, Avgelis A, Katis NI, Maliogka VI. 2017. Insights into the etiology of polerovirus-induced pepper yellows disease. Phytopathology, 107:1567-1576. doi.org/10.1094/PHYTO-07-16-0254-R.

Lotos L, Olmos A, Katis NI, Maliogka VI. 2018. First Report of Carrot torrado virus 1 and Carrot thin leaf virus Naturally Infecting Torilis arvensis ssp. arvensis in Greece. Plant Dis. Jul 31 doi: 10.1094/PDIS-03-18-0381-PDN.

Lu MG, Zhang C, Zhang ZX, Wang CA, Li SF. 2017. Nectarine stem-pitting-associated virus Detected in Peach Trees in China. Plant Dis. 101:513. doi.org/10.1094/PDIS-09-16-1256-PDN.

Luria N, Smith E, Reingold V, Bekelman I, Lapidot M, Levin I, Elad N, Tam Y, Sela N, Abu-Ras A, Ezra N, Haberman A, Yitzhak L, Lachman O, Dombrovsky A. 2017. A New Israeli Tobamovirus Isolate Infects Tomato Plants Harboring Tm-22 Resistance Genes. PLoS One 12:e0170429. doi: 10.1371/journal.pone.0170429.

Luria N, Smith E, Sela N, Lachman O, Bekelman I, Koren A, Dombrovsky A. 2018. A local strain of Paprika mild mottle virus breaks L3 resistance in peppers and is accelerated in Tomato brown rugose fruit virus-infected Tm-22-resistant tomatoes. Virus Genes 54:280-289. doi: 10.1007/s11262-018-1539-2.

Ma Y, Navarro B, Zhang Z, Lu M, Zhou X, Chi S, Di Serio F, Li S. 2015a. Identification and molecular characterization of a novel monopartite geminivirus associated with mulberry mosaic dwarf disease. J Gen Virol. 96(8):2421-34. doi: 10.1099/vir.0.000175.

Ma J, Pallett D, Jiang H, Hou Y, Wang H. 2015b. Mutational bias of Turnip Yellow Mosaic Virus in the context of host anti-viral gene silencing. Virology 486:2-6. doi: 10.1016/j.virol.2015.08.024.

Ma YX, Li SF, and Zhang ZX. 2017. First Report of Grapevine rupestris vein feathering virus in an Old Grapevine in China. Plant Dis. 101:848. doi.org/10.1094/PDIS-11-16-1620-PDN.

Maliogka VI, Olmos A, Pappi PG, Lotos L, Efthimiou K, Grammatikaki G, Candresse T, Katis NI, Avgelis AD. 2015. A novel grapevine badnavirus is associated with the Roditis leaf discoloration disease. Virus Res. 203:47-55. doi: 10.1016/j.virusres.2015.03.003.

Malpica-López N, Rajeswaran R, Beknazariants D, Seguin J, Golyaev V, Farinelli L, Pooggin MM. 2018. Revisiting the Roles of Tobamovirus Replicase Complex Proteins in Viral Replication and Silencing Suppression. Mol Plant Microbe Interact. 31:125-144. doi: 10.1094/MPMI-07-17-0164-R.

Marcon HS, Costa-Silva J, Lorenzetti APR, Marino CL, Domingues DS. 2017. Genome-wide analysis of EgEVE_1, a transcriptionally active endogenous viral element associated to small RNAs in Eucalyptus genomes. Genet Mol Biol. 40:217-225. doi: 10.1590/1678-4685-GMB-2016-0086.

Margaria P, Miozzi L, Ciuffo M, Pappu HR, Turina M. 2015. The first complete genome sequences of two distinct European tomato spotted wilt virus isolates. Arch Virol. 160: 591-5. doi: 10.1007/s00705-014-2256-3.

Margaria P, Miozzi L, Ciuffo M, Rosa C, Axtell MJ, Pappu HR, Turina M. 2016. Comparison of small RNA profiles in Nicotiana benthamiana and Solanum lycopersicum infected by polygonum ringspot tospovirus reveals host-specific responses to viral infection. Virus Res. 211:38-45. doi: 10.1016/j.virusres.2015.09.019.

Massart S, Chiumenti M, De Jonghe K, Glover R, Haegeman A, Koloniuk I, Komínek P, Kreuze J, Kutnjak D, Lotos L, Maclot F, Maliogka VI, Maree H, Olivier T, Olmos A, Pooggin M, Reynard JS, Ruiz-García AB, Safarova D, Schneeberger PH, Sela N, Turco S, Vainio EJ, Varallyai E, Verdin E, Westenberg M, Brostaux Y, Candresse T. 2018. Virus detection by high-throughput sequencing of small RNAs: large scale performance testing of sequence analysis strategies. Phytopathology. Aug 2. doi: 10.1094/PHYTO-02-18-0067-R.

Masuta Y, Nozawa K, Takagi H, Yaegashi H, Tanaka K, Ito T, Saito H, Kobayashi H, Matsunaga W, Masuda S, Kato A, Ito H. 2017. Inducible Transposition of a Heat-Activated Retrotransposon in Tissue Culture. Plant Cell Physiol. 58(2):375-384. doi: 10.1093/pcp/pcw202.

Matsumura EE, Coletta-Filho HD, Nouri S, Falk BW, Nerva L, Oliveira TS, Dorta SO, Machado MA. 2017. Deep Sequencing Analysis of RNAs from Citrus Plants Grown in a Citrus Sudden Death-Affected Area Reveals Diverse Known and Putative Novel Viruses. Viruses 9(4). pii: E92. doi: 10.3390/v9040092.

Mbanzibwa DR, Tugume AK, Chiunga E, Mark D, Tairo FD. 2014. Small RNA deep sequencing-based detection and further evidence of DNA viruses infecting sweetpotato plants in Tanzania. Ann. Appl. Biol. 165: 329–39

Meng JR, Liu PP, Zou CW, Wang ZQ, Liao YM, Cai JH, Qin BX, Chen BS. 2013. First Report of a Tospovirus in Mulberry. Plant Dis. 97:1001. doi.org/10.1094/PDIS-08-12-0792-PDN.

Miozzi L, Gambino G, Burgyan J, Pantaleo V. 2013a. Genome-wide identification of viral and host transcripts targeted by viral siRNAs in Vitis vinifera. Mol Plant Pathol. 14:30-43. doi: 10.1111/j.1364-3703.2012.00828.x.

Miozzi L, Pantaleo V, Burgyán J, Accotto GP, Noris E. 2013b. Analysis of small RNAs derived from tomato yellow leaf curl Sardinia virus reveals a cross reaction between the major viral hotspot and the plant host genome. Virus Res. 178:287-96. doi: 10.1016/j.virusres.2013.09.029.

Mitter N, Koundal V, Williams S, Pappu H. 2013. Differential expression of tomato spotted wilt virus-derived viral small RNAs in infected commercial and experimental host plants. PLoS One 8:e76276. doi: 10.1371/journal.pone.0076276.

Molina LG, da Fonseca GC, de Morais GL, de Oliveira LF, de Carvalho JB, Kulcheski FR, Margis R. 2012. Metatranscriptomic analysis of small RNAs present in soybean deep sequencing libraries. Genet Mol Biol. 35:292-303. doi: 10.1590/S1415-47572012000200010.

Montes C, Castro Á, Barba P, Rubio J, Sánchez E, Carvajal D, Aguirre C, Tapia E, DelÍ Orto P, Decroocq V, Prieto H. 2014. Differential RNAi responses of Nicotiana benthamiana individuals transformed with a hairpin-inducing construct during Plum pox virus challenge. Virus Genes 49:325-38. doi: 10.1007/s11262-014-1093-5.

Morelli M, Giampetruzzi A, Laghezza L, Catalano L, Savino VN, Saldarelli P. 2017. Identification and characterization of an isolate of apple green crinkle associated virus involved in a severe disease of quince (Cydonia oblonga, Mill.). Arch Virol. 162:299-306. doi: 10.1007/s00705-016-3074-6.

Moyle RL, Pretorius L, Carvalhais LC, Schenk PM. 2017. Complete nucleotide sequence of Australian Tomato spotted wilt virus isolate TSWV-QLD2. Genome Announc 5:e01267-17. doi.org/10.1128/genomeA.01267-17.

Mwaipopo B, Msolla NS, Njau P, Mark D, Mbanzibwa DR. 2018. Comprehensive surveys of Bean common mosaic virus and Bean common mosaic necrosis virus and molecular evidence for occurrence of other Phaseolus vulgaris viruses in Tanzania. Plant Dis. Aug. doi.org/10.1094/PDIS-01-18-0198-RE.

Navarro B, Loconsole G, Giampetruzzi A, Aboughanem-Sabanadzovic N, Ragozzino A, Ragozzino E, Di Serio F. 2017. Identification and characterization of privet leaf blotch-associated virus, a novel idaeovirus. Mol Plant Pathol. 18:925-936. doi: 10.1111/mpp.12450.

Navarro B, Minutolo M, De Stradis A, Palmisano F, Alioto D, Di Serio F. 2018. The first phlebo-like virus infecting plants: a case study on the adaptation of negative-stranded RNA viruses to new hosts. Mol Plant Pathol. 19:1075-1089. doi: 10.1111/mpp.12587.

Naveed K, Mitter N, Harper A, Dhingra A, Pappu HR. 2014. Comparative analysis of virus-specific small RNA profiles of three biologically distinct strains of Potato virus Y in infected potato (Solanum tuberosum) cv. Russet Burbank. Virus Res. 191:153-60. doi: 10.1016/j.virusres.2014.07.005.

Nhlapo TF, Mulabisana JM, Odeny DA, Rey MEC, Rees DJG. 2018. First Report of Sweet potato badnavirus A and Sweet potato badnavirus B in South Africa. Plant Dis. 102:1865. doi.org/10.1094/PDIS-08-17-1235-PDN.

Nie X, De Koeyer D, Liang Z, Dickison V, Singh M, Hawkins G. 2015. Identification and First Report of a Potato Tuber Necrosis-Inducing Isolate of Alfalfa mosaic virus in Canada. Plant Dis. 99:1658. doi.org/10.1094/PDIS-04-15-0423-PDN.

Nisbet C, Monger WA, Ross S, Holmes RF, Nova Y, Thomson C, Goodfellow HA, Lacomme C, Jeffries CJ. 2018. Biological and molecular characterization of Potato yellow blotch virus, a new species of the genus Potyvirus. Plant Pathol. Accepted. doi:10.1111/ppa.12943

Nordenstedt N, Marcenaro D, Chilagane D, Mwaipopo B, Rajamäki ML, Nchimbi-Msolla S, Njau PJR, Mbanzibwa DR, Valkonen JPT. 2017. Pathogenic seedborne viruses are rare but Phaseolus vulgaris endornaviruses are common in bean varieties grown in Nicaragua and Tanzania. PLoS One 12:e0178242. doi: 10.1371/journal.pone.0178242.

Ogwok E, Ilyas M, Alicai T, Rey ME, Taylor NJ. 2016. Comparative analysis of virus-derived small RNAs within cassava (Manihot esculenta Crantz) infected with cassava brown streak viruses. Virus Res. 215:1-11. doi: 10.1016/j.virusres.2016.01.015.

Padmanabhan C, Zheng Y, Li R, Martin GB, Fei Z, Ling KS. 2015a. Complete Genome Sequence of a Tomato-Infecting Tomato Mottle Mosaic Virus in New York. Genome Announc. 3(6). pii: e01523-15. doi: 10.1128/genomeA.01523-15.

Padmanabhan C, Zheng Y, Li R, Sun SE, Zhang D, Liu Y, Fei Z, Ling KS. 2015b. Complete genome sequence of Southern tomato virus identified in China using next-generation sequencing. Genome Announc 3(5):e01226-15. doi:10.1128/genomeA.01226-15.

Pallett DW, Ho T, Cooper I, Wang H. 2010. Detection of Cereal yellow dwarf virus using small interfering RNAs and enhanced infection rate with Cocksfoot streak virus in wild cocksfoot grass (Dactylis glomerata). J Virol Methods 168:223-7. doi: 10.1016/j.jviromet.2010.06.003.

Pantaleo V, Saldarelli P, Miozzi L, Giampetruzzi A, Gisel A, Moxon S, Dalmay T, Bisztray G, Burgyan J. 2010. Deep sequencing analysis of viral short RNAs from an infected Pinot Noir grapevine. Virology 408: 49-56. doi: 10.1016/j.virol.2010.09.001.

Patil BL, Arora DJ. 2018. Comparative characterization of small RNAs derived from an emaravirus and a geminivirus infecting pigeonpea. Plant Biochem Biotechnol. https://doi.org/10.1007/s13562-018-0447-9

Pecman A, Kutnjak D, Gutiérrez-Aguirre I, Adams I, Fox A, Boonham N, Ravnikar M. 2017. Next Generation Sequencing for Detection and Discovery of Plant Viruses and Viroids: Comparison of Two Approaches. Front Microbiol. 8:1998. doi: 10.3389/fmicb.2017.01998.

Peña E, Gutiérrez M, Montecinos A, Muñoz M, Vargas E, Acuña I, Gutiérrez RA, Rosales IM. 2016. First Report of Potato mop-top virus in Chile. Plant Dis. 100:1250. doi.org/10.1094/PDIS-07-15-0816-PDN.

Pérez-Cañamás M, Blanco-Pérez M, Forment J, Hernández C. 2017. Nicotiana benthamiana plants asymptomatically infected by Pelargonium line pattern virus show unusually high accumulation of viral small RNAs that is neither associated with DCL induction nor RDR6 activity. Virology 501:136-146. doi: 10.1016/j.virol.2016.11.018.

Pirovano W, Miozzi L, Boetzer M, Pantaleo V. 2015. Bioinformatics approaches for viral metagenomics in plants using short RNAs: model case of study and application to a Cicer arietinum population. Front Microbiol. 5:790. doi: 10.3389/fmicb.2014.00790.

Qi X, Bao FS, Xie Z. 2009. Small RNA deep sequencing reveals role for Arabidopsis thaliana RNA-dependent RNA polymerases in viral siRNA biogenesis. PLoS One 4:e4971. doi: 10.1371/journal.pone.0004971.

Qiao W, Zarzyńska-Nowak A, Nerva L, Kuo YW, Falk BW. 2018. Accumulation of 24 nucleotide transgene-derived siRNAs is associated with crinivirus immunity in transgenic plants. Mol Plant Pathol. Apr 28. doi: 10.1111/mpp.12695.

Qin YH, Li XC, Zhang ZC, Qiao Q, Zhang DS, Wang YJ, Tian YT, Wang S. 2016. First Report of Sweet potato badnavirus A in China. Plant Dis. 100:865. doi.org/10.1094/PDIS-09-15-1081-PDN.

Qin C, Li B, Fan Y, Zhang X, Yu Z, Ryabov E, Zhao M, Wang H, Shi N, Zhang P, Jackson S, Tör M, Cheng Q, Liu Y, Gallusci P, Hong Y. 2017. Roles of Dicer-Like Proteins 2 and 4 in Intra- and Intercellular Antiviral Silencing. Plant Physiol. 174:1067-1081. doi: 10.1104/pp.17.00475.

Qiu Y, Zhang Y, Hu F, Zhu S. 2017. Characterization of siRNAs derived from cucumber mosaic virus in infected tobacco plants. Arch Virol. 162:2077-2082. doi: 10.1007/s00705-017-3335-z.

Qiu Y, Wu Y, Zhang Y, Xu W, Wang C, Zhu S. 2018. Profiling of small RNAs derived from cucumber mosaic virus in infected Nicotiana benthamiana plants by deep sequencing. Virus Res. 252:1-7. doi: 10.1016/j.virusres.2018.05.013.

Rajeswaran R, Golyaev V, Seguin J, Zvereva AS, Farinelli L, Pooggin MM. 2014a. Interactions of Rice tungro bacilliform pararetrovirus and its protein P4 with plant RNA-silencing machinery. Mol Plant Microbe Interact. 27: 1370-8. doi: 10.1094/MPMI-07-14-0201-R.

Rajeswaran R, Seguin J, Chabannes M, Duroy PO, Laboureau N, Farinelli L, Iskra-Caruana ML, Pooggin MM. 2014b. Evasion of short interfering RNA-directed antiviral silencing in Musa acuminata persistently infected with six distinct banana streak pararetroviruses. J Virol. 88:11516-28. doi: 10.1128/JVI.01496-14.

Ramos-González PL, Chabi-Jesus C, Guerra-Peraza O, Breton MC, Arena GD, Nunes MA, Kitajima EW, Machado MA, Freitas-Astúa J. 2016. Phylogenetic and Molecular Variability Studies Reveal a New Genetic Clade of Citrus leprosis virus C. Viruses 8(6). pii: E153. doi: 10.3390/v8060153.

Reingold V, Lachman O, Sela N, Luria N, Dombrovsky A. 2016. Watermelon fruit rot disease in Israel is caused by a distinct Squash vein yellowing virus (SqVYV) strain. Plant Disease 100:1176-1183. doi: dx.doi.org/10.1094/PDIS-09-15-1040-RE.

Reynard JS, Schumacher S, Menzel W, Fuchs J, Bohnert P, Glasa M, Wetzel T, Fuchs R. 2016. First Report of Grapevine Pinot gris virus in German Vineyards. Plant Dis. 100:2545. doi.org/10.1094/PDIS-07-16-0966-PDN.

Rogans SJ, Allie F, Tirant JE, Rey MEC. 2016. Small RNA and methylation responses in susceptible and tolerant landraces of cassava infected with South African cassava mosaic virus. Virus Res. 225:10-22. doi: 10.1016/j.virusres.2016.08.011.

Romanel E, Silva TF, Corrêa RL, Farinelli L, Hawkins JS, Schrago CE, Vaslin MF. 2012. Global alteration of microRNAs and transposon-derived small RNAs in cotton (Gossypium hirsutum) during Cotton leafroll dwarf polerovirus (CLRDV) infection. Plant Mol Biol. 80:443-60. doi: 10.1007/s11103-012-9959-1.

Roy A, Choudhary N, Guillermo LM, Shao J, Govindarajulu A, Achor D, Wei G, Picton DD, Levy L, Nakhla MK, Hartung JS, Brlansky RH. 2013a. A novel virus of the genus Cilevirus causing symptoms similar to citrus leprosis. Pytopathology 103:488-500. doi: 10.1094/PHYTO-07-12-0177-R.

Roy A, Shao J, Hartung JS, Schneider W, Brlansky RH. 2013b. A Case Study on Discovery of Novel Citrus Leprosis Virus Cytoplasmic Type 2 Utilizing Small RNA Libraries by Next Generation Sequencing and Bioinformatic Analyses. J Data Mining Genomics Proteomics 4:129. doi:10.4172/2153-0602.1000129.

Roy A, Stone A, Otero-Colina G, Wei G, Choudhary N, Achor D, Shao J, Levy L, Nakhla MK, Hollingsworth CR, Hartung JS, Schneider WL, Brlansky RH. 2013c. Genome assembly of citrus leprosis virus nuclear type reveals a close association with orchid fleck virus. Genome Announc. 1(4). pii: e00519-13. doi: 10.1128/genomeA.00519-13.

Roy A, Stone AL, León Martínez G, Hartung JS, Wei G, Mavrodieva VA, Nakhla MK, Schneider WL, Brlansky RH. 2018a. First Report of Hibiscus-infecting cilevirus in Citrus sinensis in Meta and Casanare, Colombia. Plant Dis. 102:1675. doi.org/10.1094/PDIS-01-18-0150-PDN.

Roy A, Stone AL, Melzer MJ, Hartung JS, Mavrodieva VA, Nakhla MK, Schneider WL, Brlansky RH. 2018b. First Report of a Cilevirus Associated with Green Ringspot on Senescent Hibiscus Leaves in Tampa, Florida. Plant Dis. 102:1181. doi.org/10.1094/PDIS-11-17-1699-PDN.

Ruiz L, Simón A, García C, Velasco L, Janssen D. 2018. First natural crossover recombination between two distinct species of the family Closteroviridae leads to the emergence of a new disease. PLoS One 13:e0198228. doi: 10.1371/journal.pone.0198228.

Ruiz-Garcia AB, Martinez C, Santiago R, Garcia MT, de Prado N, Olmos A. 2016. First report of Little cherry virus 1 (LChV-1) in sweet cherry in Spain. Plant Dis. 100:2340. doi: doi.org/10.1094/PDIS-05-16-0620-PDN.

Ruiz-Garcia AB, Chamberland N, Martinez C, Massart S, Olmos A. 2017. First report of Persimmon cryptic virus in Spain. J Plant Pathol. 99:287. doi: dx.doi.org/10.4454/jpp.v99i1.3802

Ruiz-Ruiz S, Navarro B, Gisel A, Peña L, Navarro L, Moreno P, Di Serio F, Flores R. 2011. Citrus tristeza virus infection induces the accumulation of viral small RNAs (21-24-nt) mapping preferentially at the 3'-terminal region of the genomic RNA and affects the host small RNA profile. Plant Mol Biol. 75:607-19. doi: 10.1007/s11103-011-9754-4.

Sánchez E, Tricon D, Mora R, Quiroz D, Decroocq V, Prieto H. 2017. A fast and efficient protocol for small RNA extraction in Japanese plum and other Prunus species. Electronic Journal of Biotechnology 30:103-109. doi.org/10.1016/j.ejbt.2017.10.006

Santala J, Valkonen JPT. 2018. Sensitivity of Small RNA-Based Detection of Plant Viruses. Front Microbiol. 9:939. doi: 10.3389/fmicb.2018.00939.

Saldarelli P, Giampetruzzi A, Morelli M, Malossini U, Pirolo C, Bianchedi P, Gualandri V. 2015. Genetic Variability of Grapevine Pinot gris virus and Its Association with Grapevine Leaf Mottling and Deformation. Phytopathology 105:555-563. doi.org/10.1094/PHYTO-09-14-0241-R.

Saqib M, Wylie SJ, Jones MG. 2015. Serendipitous identification of a new Iflavirus‐like virus infecting tomato and its subsequent characterization. Plant Pathol. 64:519-527. doi: 10.1111/ppa.12293.

Seguin J, Rajeswaran R, Malpica-López N, Martin RR, Kasschau K, Dolja VV, Otten P, Farinelli L, Pooggin MM. 2014. De novo reconstruction of consensus master genomes of plant RNA and DNA viruses from siRNAs. PLoS One 9:e88513. doi: 10.1371/journal.pone.0088513.

Sela N, Luria N, Dombrovsky A. 2012. Genome assembly of bell pepper endornavirus from small RNA. J Virol. 86:7721. doi: 10.1128/JVI.00983-12

Silva TF, Romanel EA, Andrade RR, Farinelli L, Østerås M, Deluen C, Corrêa RL, Schrago CE, Vaslin MF. 2011. Profile of small interfering RNAs from cotton plants infected with the polerovirus Cotton leafroll dwarf virus. BMC Mol Biol. 12:40. doi: 10.1186/1471-2199-12-40.

Shchetynina A, Budzanivska I, Pereboychuk O, Sõmera M, Truve E. 2017. First report of Hosta virus X infecting hosta plants in Ukraine. Acta Virol. 61:498-499. doi: 10.4149/av_2017_415.

Shen WX, Au PC, Shi BJ, Smith NA, Dennis ES, Guo HS, Zhou CY, Wang MB. 2015. Satellite RNAs interfere with the function of viral RNA silencing suppressors. Front Plant Sci. 6:281. doi: 10.3389/fpls.2015.00281.

Shi Y, Sun XY, Wang ZY, Li HL, Yan ZL. 2016. First Report of Zucchini yellow mosaic virus Infecting Sesame in China. Plant Dis. 100:2545. doi.org/10.1094/PDIS-07-16-0970-PDN.

Shimura H, Pantaleo V, Ishihara T, Myojo N, Inaba J, Sueda K, Burgyán J, Masuta C. 2011. A viral satellite RNA induces yellow symptoms on tobacco by targeting a gene involved in chlorophyll biosynthesis using the RNA silencing machinery. PLoS Pathog. 7:e1002021. doi: 10.1371/journal.ppat.1002021.

Smith NA, Eamens AL, Wang MB. 2011. Viral small interfering RNAs target host genes to mediate disease symptoms in plants. PLoS Pathog. 7:e1002022. doi: 10.1371/journal.ppat.1002022.

Smith O, Clapham A, Rose P, Liu Y, Wang J, Allaby RG. 2014. A complete ancient RNA genome: identification, reconstruction and evolutionary history of archaeological Barley Stripe Mosaic Virus. Sci Rep. 4:4003. doi: 10.1038/srep04003

Sõmera M, Gantsovski M, Truve E, Sooväli P. 2016. First Report of Brome mosaic virus in Wheat in Estonia. Plant Disease 100:2175. doi: 10.1094/PDIS-04-16-0426-PDN.

Su X, Fu S, Qian Y, Zhang L, Xu Y, Zhou X. 2016. Discovery and small RNA profile of Pecan mosaic-associated virus, a novel potyvirus of pecan trees. Sci Rep. 6:26741. doi: 10.1038/srep26741.

Sun F, Guo W, Du J, Ni Z, Sun Q, Yao Y. 2013. Widespread, abundant, and diverse TE-associated siRNAs in developing wheat grain. Gene 522:1-7. doi: 10.1016/j.gene.2013.03.101.

Sun SE, Wang JQ, Chen S, Zhang SB, Zhang DY, Liu Y. 2018. First Report of Capsicum chlorosis orthotospovirus Infecting Zucchini (Cucurbita pepo) in China. Plant Dis. 14 Aug. doi.org/10.1094/PDIS-12-17-1876-PDN.

Šurbanovski N, Brilli M, Moser M, Si-Ammour A. 2016. A highly specific microRNA-mediated mechanism silences LTR retrotransposons of strawberry. Plant J. 85:70-82. doi: 10.1111/tpj.13090.

Susi H, Laine AL, Filloux D, Kraberger S, Farkas K, Bernardo P, Frilander MJ, Martin DP, Varsani A, Roumagnac P. 2017. Genome sequences of a capulavirus infecting Plantago lanceolata in the Åland archipelago of Finland. Arch Virol. 162:2041-2045. doi: 10.1007/s00705-017-3298-0.

Susi H, Rajamäki ML, Artola K, Jayaraj‐Mallika FR, Valkonen JPT. 2018. Molecular detection and characterisation of black raspberry necrosis virus and raspberry bushy dwarf virus isolates in wild raspberries. Ann Appl Biol 173: 97-111. doi: 10.1111/aab.12438.

Szittya G, Moxon S, Pantaleo V, Toth G, Rusholme Pilcher RL, Moulton V, Burgyan J, Dalmay T. 2010. Structural and functional analysis of viral siRNAs. PLoS Pathog. 6:e1000838. doi: 10.1371/journal.ppat.1000838.

Tang QJ, Liu MY, Li X, Zhao SQ, Dai LY. 2017. Pumpkin: A New Natural Host of Tobacco mild green mosaic virus in China. Plant Dis. 101:1063-1063. doi.org/10.1094/PDIS-03-16-0317-PDN.

Tatineni S, Riethoven JJ, Graybosch RA, French R, Mitra A. 2014. Dynamics of small RNA profiles of virus and host origin in wheat cultivars synergistically infected by Wheat streak mosaic virus and Triticum mosaic virus: virus infection caused a drastic shift in the endogenous small RNA profile. PLoS One 9:e111577. doi: 10.1371/journal.pone.0111577.

Turco S, Golyaev V, Seguin J, Gilli C, Farinelli L, Boller T, Schumpp O, Pooggin MM. 2018. Small RNA-Omics for Virome Reconstruction and Antiviral Defense Characterization in Mixed Infections of Cultivated Solanum Plants. Mol Plant Microbe Interact. 31:707-723. doi: 10.1094/MPMI-12-17-0301-R.

Untiveros M, Olspert A, Artola K, Firth AE, Kreuz JF, Valkonen JP. 2016. A novel sweet potato potyvirus open reading frame (ORF) is expressed via polymerase slippage and suppresses RNA silencing. Mol Plant Pathol. 17:1111-1123. doi: 10.1111/mpp.12366

Vargas-Asencio J, Wojciechowska K, Baskerville M, Gomez AL, Perry KL, Thompson JR. 2017b. The complete nucleotide sequence and genomic characterization of grapevine asteroid mosaic associated virus. Virus Res. 227:82-87. doi: 10.1016/j.virusres.2016.

Varveri C, Olmos A, Pina JA, Marroquín C, Cambra M. 2015. Biological and molecular characterization of a distinct Citrus tristeza virus isolate originating from a lemon tree in Greece. Plant Pathol. 64:792-798. doi:10.1111/ppa.12308.

Velasco L, Bota J, Montero R, and Cretazzo E. 2014. Differences of Three Ampeloviruses' Multiplication in Plant May Explain Their Incidences in Vineyards. Plant Dis. 98:395-400. doi.org/10.1094/PDIS-04-13-0433-RE.

Velasco L, Salem N, Willemsen A, Lapidot M, Mansour AN, Rubio L, Galipienso L. 2016. Genetic variation and evolutionary forces shaping Cucumber vein yellowing virus populations: risk of emergence of virulent isolates in Europe. Plant Pathol. 65:847-856. doi: 10.1111/ppa.12465.

Verdin E, Wipf-Scheibel C, Gognalons P, Aller F, Jacquemond M, Tepfer M. 2017. Sequencing viral siRNAs to identify previously undescribed viruses and viroids in a panel of ornamental plant samples structured as a matrix of pools. Virus Res. 241:19-28. doi: 10.1016/j.virusres.2017.05.019.

Verdin E, Marais A, Wipf-Scheibel C, Faure C, Pelletier B, David P, Svanella-Dumas L, Poisblaud C, Lecoq H, Candresse T. 2018. Biological and Genetic Characterization of New and Known Necroviruses Causing an Emerging Systemic Necrosis Disease of Corn Salad (Valerianella locusta) in France. Phytopathology 108:1002-1010. doi: 10.1094/PHYTO-08-17-0284-R.

Visser M, Maree HJ, Rees DJ, Burger JT. 2014. High-throughput sequencing reveals small RNAs involved in ASGV infection. BMC Genomics 15:568. doi: 10.1186/1471-2164-15-568.

Visser M, Bester R, Burger JT, Maree HJ. 2016. Next-generation sequencing for virus detection: covering all the bases. Virol J. 13:85. doi: 10.1186/s12985-016-0539-x.

Visser M, Cook G, Burger JT, Maree HJ. 2017. In silico analysis of the grapefruit sRNAome, transcriptome and gene regulation in response to CTV-CDVd co-infection. Virol J. 14:200. doi: 10.1186/s12985-017-0871-9.

Vives MC, Velázquez K, Pina JA, Moreno P, Guerri J, Navarro L. 2013. Identification of a new enamovirus associated with citrus vein enation disease by deep sequencing of small RNAs. Phytopathology 103(10):1077-86. doi: 10.1094/PHYTO-03-13-0068-R.

Waldron FM, Stone GN, Obbard DJ. 2018. Metagenomic sequencing suggests a diversity of RNA interference-like responses to viruses across multicellular eukaryotes. PLoS Genet. 14:e1007533. doi.org/10.1371/journal.pgen.1007533.

Wang XB, Wu Q, Ito T, Cillo F, Li WX, Chen X, Yu JL, Ding SW. 2010. RNAi-mediated viral immunity requires amplification of virus-derived siRNAs in Arabidopsis thaliana. Proc Natl Acad Sci U S A. Jan 5;107(1):484-9. doi: 10.1073/pnas.0904086107.

Wang XB, Jovel J, Udomporn P, Wang Y, Wu Q, Li WX, Gasciolli V, Vaucheret H, Ding SW. 2011. The 21-nucleotide, but not 22-nucleotide, viral secondary small interfering RNAs direct potent antiviral defense by two cooperative argonautes in Arabidopsis thaliana. Plant Cell 23:1625-38. doi: 10.1105/tpc.110.082305.

Wang Y, Cheng X, Wu X, Wang A, Wu X. 2014. Characterization of complete genome and small RNA profile of pagoda yellow mosaic associated virus, a novel badnavirus in China. Virus Res 188:103–108. doi: 10.1016/j.virusres.2014.04.006.

Wang YJ, Zhang DS, Zhang ZC, Wang S, Qiao Q, Qin YH, Tian YT. 2015a. First Report on Sweetpotato symptomless virus 1 (Genus Mastrevirus, Family Geminiviridae) in Sweetpotato in China. Plant Dis. 99:1042. doi.org/10.1094/PDIS-12-14-1358-PDN.

Wang F, Wu QF, Zhou BG, Gao ZL, and Xu DF. 2015b. First Report of Turnip yellows virus in Tobacco in China. Plant Dis. 99:1870. doi.org/10.1094/PDIS-04-15-0426-PDN.

Wang J, Tang Y, Yang Y, Ma N, Ling X, Kan J, He Z, Zhang B. 2016a. Cotton Leaf Curl Multan Virus-Derived Viral Small RNAs Can Target Cotton Genes to Promote Viral Infection. Front Plant Sci. 7:1162. doi: 10.3389/fpls.2016.01162.

Wang J, Zhai Y, Liu W, Dhingra A, Pappu HR, Liu Q. 2016b. Structure and Genome Organization of Cherry Virus A (Capillovirus, Betaflexiviridae) from China Using Small RNA Sequencing. Genome Announc. 4(3). pii: e00364-16.

Wang J, Zhai Y, Liu W, Zhu D, Pappu HR, Liu Q. 2016c. Complete Genomic Characterization of Plum bark necrosis stem pitting-associated virus Infecting Sweet Cherry in China. Genome Announc. 4(3). pii: e00413-16. doi: 10.1128/genomeA.00413-16.

Wang J, Zhu D, Tan Y, Zong X, Wei H, Hammond RW, Liu Q. 2016d. Complete nucleotide sequence of little cherry virus 1 (LChV-1) infecting sweet cherry in China. Arch Virol. 161:749-53. doi: 10.1007/s00705-015-2737-z.

Wang J, Zhu D. Tan Y, Zong X, Wei H, Liu Q. 2016e. First Report of Citrus leaf blotch virus in Sweet Cherry. Plant Dis. 100:1027. doi.org/10.1094/PDIS-09-15-0965-PDN.

Wang F, Zhou BG, Gao Z.L, Xu DF. 2016f. A New Species of the Genus Polerovirus Causing Symptoms Similar to Maize yellow dwarf virus-RMV of Maize in China. Plant Dis. 100:1508. doi.org/10.1094/PDIS-11-15-1259-PDN.

Wang HY, Gong HH, Yan ZY, Tang W, Zhu TS, Zhao M, and Li XD. 2017. First Report of Tobacco vein banding mosaic virus Infecting Sesame in China. Plant Dis. 101:850. doi.org/10.1094/PDIS-12-16-1707-PDN.

Wang Y, Liang W, Tang T. 2018a. Constant conflict between Gypsy LTR retrotransposons and CHH methylation within a stress-adapted mangrove genome. New Phytol. May 15. doi: 10.1111/nph.15209.

Wang J, Zhai Y, Zhu D, Liu W, Pappu HR, Liu Q. 2018b. Whole-Genome Characterization of Prunus necrotic ringspot virus Infecting Sweet Cherry in China. Genome Announc. 6(9). pii: e00060-18.

Wang T, Deng Z, Zhang X, Wang H, Wang Y, Liu X, Liu S, Xu F, Li T, Fu D, Zhu B, Luo Y, Zhu H. 2018c. Tomato DCL2b is required for the biosynthesis of 22-nt small RNAs, the resulting secondary siRNAs, and the host defense against ToMV. Hortic Res. 5:62. doi: 10.1038/s41438-018-0073-7.

Wang Y, Zhuang H, Yang Z, Wen L, Wang G, Hong N. 2018d. Molecular characterization of an Apple stem grooving virus isolate from kiwifruit (Actinidia chinensis) in China. Can J Plant Pathol. 40:1, 76-83, doi: 10.1080/07060661.2017.1401006.

Wu J, Yang Z, Wang Y, Zheng L, Ye R, Ji Y, Zhao S, Ji S, Liu R, Xu L, Zheng H, Zhou Y, Zhang X, Cao X, Xie L, Wu Z, Qi Y, Li Y. 2015b. Viral-inducible Argonaute18 confers broad-spectrum virus resistance in rice by sequestering a host microRNA. Elife 4. doi: 10.7554/eLife.05733.

Wu X, Cheng X, Li W. 2018a. First Report of Natural Infection of Watermelon mosaic virus on Lagerstroemia indica in China. Plant Dis. 102:1048. doi.org/10.1094/PDIS-10-17-1604-PDN.

Wu X, Liu Q, Chai M, Liu J, Zhang L, Cheng X. 2018b. First report of Potato aucuba mosaic virus on potato in China. Plant Diesase. doi: doi.org/10.1094/PDIS-05-18-0851-PDN.

Xia Z, Peng J, Li Y, Chen L, Li S, Zhou T, Fan Z. 2014. Characterization of small interfering RNAs derived from Sugarcane mosaic virus in infected maize plants by deep sequencing. PLoS One 9:e97013. doi: 10.1371/journal.pone.0097013.

Xia Z, Zhao Z, Chen L, Li M, Zhou T, Deng C, Zhou Q, Fan Z. 2016. Synergistic infection of two viruses MCMV and SCMV increases the accumulations of both MCMV and MCMV-derived siRNAs in maize. Sci Rep. 6:20520. doi: 10.1038/srep20520.

Xin M, Cao M, Liu W, Ren Y, Zhou X, Wang X. 2017a. Two Negative-Strand RNA Viruses Identified in Watermelon Represent a Novel Clade in the Order Bunyavirales. Front Microbiol. 8:1514. doi: 10.3389/fmicb.2017.01514.

Xin M, Cao M, Liu W, Ren Y, Lu C, Wang X. 2017b. The genomic and biological characterization of Citrullus lanatus cryptic virus infecting watermelon in China. Virus Res. 232:106-112. doi: 10.1016/j.virusres.2017.02.009.

Xin M, Zhang P, Liu W, Ren Y, Cao M, Wang X. 2017c. The complete nucleotide sequence and genome organization of a novel betaflexivirus infecting Citrullus lanatus. Arch Virol. Oct;162(10):3239-3242. doi: 10.1007/s00705-017-3461-7.

Xu Y, Huang L, Fu S, Wu J, Zhou X. 2012. Population diversity of rice stripe virus-derived siRNAs in three different hosts and RNAi-based antiviral immunity in Laodelphgax striatellus. PLoS One 7:e46238. doi: 10.1371/journal.pone.0046238.

Xu J, Liu D, Zhang Y, Wang Y, Han C, Li D, Yu JL, Wang XB. 2016. Improved Pathogenicity of a Beet Black Scorch Virus Variant by Low Temperature and Co-infection with Its Satellite RNA. Front Microbiol. 7:1771.

Xu C, Sun X, Taylor A, Jiao C, Xu Y, Cai X, Wang X, Ge C, Pan G, Wang Q, Fei Z, Wang Q. 2017. Diversity, Distribution, and Evolution of Tomato Viruses in China Uncovered by Small RNA Sequencing. J Virol. 91(11). pii: e00173-17. doi: 10.1128/JVI.00173-17

Xu D, Zhou G. 2017. Characteristics of siRNAs derived from Southern rice black-streaked dwarf virus in infected rice and their potential role in host gene regulation. Virol J. 14:27. doi: 10.1186/s12985-017-0699-3.

Yan F, Zhang H, Adams MJ, Yang J, Peng J, Antoniw JF, Zhou Y, Chen J. 2010. Characterization of siRNAs derived from rice stripe virus in infected rice plants by deep sequencing. Arch Virol. 155:935-40. doi: 10.1007/s00705-010-0670-8.

Yan T, Zhu JR, Di D, Gao Q, Zhang Y, Zhang A, Yan C, Miao H, Wang XB. 2015. Characterization of the complete genome of Barley yellow striate mosaic virus reveals a nested gene encoding a small hydrophobic protein. Virology 478:112-22. doi: 10.1016/j.virol.2014.12.042.

Yang X, Wang Y, Guo W, Xie Y, Xie Q, Fan L, Zhou X. 2011. Characterization of small interfering RNAs derived from the geminivirus/betasatellite complex using deep sequencing. PLoS One 6:e16928. doi: 10.1371/journal.pone.0016928.

Yang J, Zheng SL, Zhang HM, Liu XY, Li J, Li JM, Chen JP. 2014. Analysis of small RNAs derived from Chinese wheat mosaic virus. Arch Virol. 159:3077-82. doi: 10.1007/s00705-014-2155-7.

Yang X, Huang J, Liu C, Chen B, Zhang T, Zhou G. 2017. Rice Stripe Mosaic Virus, a Novel Cytorhabdovirus Infecting Rice via Leafhopper Transmission. Front Microbiol. 7:2140. doi: 10.3389/fmicb.2016.02140.

Yang C, Han T, Fu J, Liao Y, Chen S. 2018a. Characterization of the complete genome of euonymus yellow vein associated virus, a distinct member of the genus Potexvirus, family Alphaflexiviridae, isolated from Euonymus bungeanus Maxim in Liaoning, Northern China. Arch Virol. 163:563-566. doi: 10.1007/s00705-017-3617-5.

Yang K, Shen W, Li Y, Li Z, Miao W, Wang A, Cui H. 2018b. A novel virus, classified within a putative new genus of the family Potyviridae, is associated with necrotic ringspot disease in areca palm. Phytopathology Aug 22. doi: 10.1094/PHYTO-06-18-0200-R.

Yokomi RK, Selvaraj V, Maheshwari Y, Saponari M, Giampetruzzi A, Chiumenti M, Hajeri S. 2017. Identification and Characterization of Citrus tristeza virus Isolates Breaking Resistance in Trifoliate Orange in California. Phytopathology 107:901-908. doi: 10.1094/PHYTO-01-17-0007-R.

Yuan W, Du K, Fan Z, Zhou T. 2017. Complete genomic sequence of common reed chlorotic stripe virus, a novel member of the family Potyviridae. Arch Virol. 162:3541-3544. doi: 10.1007/s00705-017-3454-6.

Zaagueri T, Miozzi L, Mnari-Hattab M, Noris E, Accotto GP, Vaira AM. 2017. Deep Sequencing Data and Infectivity Assays Indicate that Chickpea Chlorotic Dwarf Virus is the Etiological Agent of the "Hard Fruit Syndrome" of Watermelon. Viruses 9(11). pii: E311. doi: 10.3390/v9110311.

Zahid K, Zhao JH, Smith NA, Schumann U, Fang YY, Dennis ES, Zhang R, Guo HS, Wang MB. 2015. Nicotiana small RNA sequences support a host genome origin of cucumber mosaic virus satellite RNA. PLoS Genet. 11:e1004906. doi: 10.1371/journal.pgen.1004906.

Zamorano A, Chiumenti M, Fernández C, Quiroga N, Pino AM, Sagredo K, Saldarelli P, Fiore N. 2017. First Report of Cherry virus A and Plum bark necrosis stem pitting-associated virus in Cherry in Chile. Plant Dis. 101:1685. doi.org/10.1094/PDIS-04-17-0533-PDN.

Zarreen F, Kumar G, Johnson AMA, Dasgupta I. 2018. Small RNA-based interactions between rice and the viruses which cause the tungro disease. Virology 523:64-73. doi: 10.1016/j.virol.2018.07.022.

Zavallo D, Debat HJ, Conti G, Manacorda CA, Rodriguez MC, Asurmendi S. 2015. Differential mRNA Accumulation upon Early Arabidopsis thaliana Infection with ORMV and TMV-Cg Is Associated with Distinct Endogenous Small RNAs Level. PLoS One 10:e0134719. doi: 10.1371/journal.pone.0134719.

Zhang Y, Singh K, Kaur R, Qiu W. 2011. Association of a novel DNA virus with the grapevine vein-clearing and vine decline syndrome. Phytopathology 101:1081-90. doi: 10.1094/PHYTO-02-11-0034.

Zhang Z, Qi S, Tang N, Zhang X, Chen S, Zhu P, Ma L, Cheng J, Xu Y, Lu M, Wang H, Ding SW, Li S, Wu Q. 2014a. Discovery of replicating circular RNAs by RNA-seq and computational algorithms. PLoS Pathog. 10:e1004553. doi: 10.1371/journal.ppat.1004553.

Zhang Y, Yu N, Huang Q, Yin G, Guo A, Wang X, Xiong Z, Liu Z. 2014b. Complete genome of Hainan papaya ringspot virus using small RNA deep sequencing. Virus Genes 48:502-8. doi: 10.1007/s11262-014-1042-3.

Zhang SB, Zhao ZB, Zhang DY, Liu Y, Zhang SB, Zhang DY, Liu Y, Luo XW, Liu J, Wu LF, Peng J. 2015. First Report of Pepper vein yellows virus Infecting Red Pepper in Mainland China. Plant Dis. 99:1190-1190. doi.org/10.1094/PDIS-01-15-0025-PDN.

Zhang SB, Liu J, Zhao ZB, Zheng LM, Zhang DY, Liu Y, Du J, Peng J, Yan F, Li F, Xie Y, Cheng ZB. 2016a. First Report of Pepper (Capsicum annuum) as a Natural Host Plant for Youcai mosaic virus Plant Dis. 100:541. doi.org/10.1094/PDIS-07-15-0795-PDN.

Zhang SB, Zhao ZB, Zheng LM, Zhang DY, Liu Y, Liu J, Du J, Peng J, Yan F, Li F, Xie Y, Cheng ZB. 2016b. First Report of Pepper veinal mottle virus Infecting Pepper in Mainland China. Plant Dis. 100:1025-1025. doi.org/10.1094/PDIS-08-15-0870-PDN.

Zhang P, Liu Y, Liu W, Cao M, Massart S, Wang X. 2017a. Identification, Characterization and Full-Length Sequence Analysis of a Novel Polerovirus Associated with Wheat Leaf Yellowing Disease. Front Microbiol. 8:1689. doi: 10.3389/fmicb.2017.01689.

Zhang SB, Zhang DY, Liu Y, Luo XW, Liu MY, Du J, Wang MC. 2017b. First report of Lettuce chlorosis virus infecting tomato in China. Plant Dis. 101:846-846. doi.org/10.1094/PDIS-09-16-1315-PDN.

Zhang S, Shen P, Li M, Tian X, Zhou C, Cao M. 2018. Discovery of a novel geminivirus associated with camellia chlorotic dwarf disease. Arch Virol. 163:1709-1712. doi: 10.1007/s00705-018-3780-3.

Zhao D, Song GQ. 2014a. High-throughput sequencing as an effective approach in profiling small RNAs derived from a hairpin RNA expression vector in woody plants. Plant Sci. 228:39-47. doi: 10.1016/j.plantsci.2014.02.013.

Zhao D, Song GQ. 2014b. Rootstock-to-scion transfer of transgene-derived small interfering RNAs and their effect on virus resistance in nontransgenic sweet cherry. Plant Biotechnol J. 12(9):1319-28. doi: 10.1111/pbi.12243.

Zhao G, Tuo D, Yan P, Li X, Zhou P, Shen W. 2018. Profile of siRNAs derived from green fluorescent protein (GFP)-tagged Papaya leaf distortion mosaic virus in infected papaya plants. Virus Genes. Sep 14. doi: 10.1007/s11262-018-1601-0.

Zheng YZ, Wang GP, Hong N, Zhou JF, Yang ZK, Hong N. 2014. First Report of Actinidia virus A and Actinidia virus B on Kiwifruit in China. Plant Dis. 98:1590. doi.org/10.1094/PDIS-04-14-0420-PDN.

Zheng Y, Gao S, Padmanabhan C, Li R, Galvez M, Gutierrez D, Fuentes S, Ling KS, Kreuze J, Fei Z. 2017a. VirusDetect: An automated pipeline for efficient virus discovery using deep sequencing of small RNAs. Virology 500:130-138. doi: 10.1016/j.virol.2016.10.017

Zheng Y, Navarro B, Wang G, Wang Y, Yang Z, Xu W, Zhu C, Wang L, Serio FD, Hong N. 2017b. Actinidia chlorotic ringspot-associated virus: a novel emaravirus infecting kiwifruit plants. Mol Plant Pathol. 18:569-581. doi: 10.1111/mpp.12421.

Zhou CJ, Zhang XY, Liu SY, Wang Y, Li DW, Yu JL, Han CG. 2017. Synergistic infection of BrYV and PEMV 2 increases the accumulations of both BrYV and BrYV-derived siRNAs in Nicotiana benthamiana. Sci Rep. 7:45132. doi: 10.1038/srep45132.

Zhuo T, Zhu LJ, Lu CC, Jiang CY, Chen ZY, Zhang G, Wang ZH, Jovel J, Han YH. 2018. Complete nucleotide sequence of jasmine virus H, a new member of the family Tombusviridae. Arch Virol. 163:731-735. doi: 10.1007/s00705-017-3663-z.
