# Supplementary material for: Small RNA-Omics for Plant Virus Identification, Virome Reconstruction, and Antiviral Defense Characterization
Source: Front Microbiol. 2018 Nov 20;9:2779. doi: 10.3389/fmicb.2018.02779 (PMC6256188; doi:10.3389/fmicb.2018.02779)
Supplement: Supplementary file 2 [file Table_2.DOCX]

**Supplementary Table S2. Viroids identified in naturally- or experimentally-infected plant hosts and fully or partially reconstructed by small RNA sequencing and assembly**

***** in most cases, 2 or 3 major size-classes of small interfering (si)RNAs (21-, 22- and 24-nucleotide, nt) are indicated in the order of their relative abundance (note that only nuclear viroids normally spawn 24-nt siRNAs); strand bias of siRNA production is indicated only if it is strong, otherwise “both strands” are indicated.

** if more than one reference, first appear those reporting more detailed information on the viroid siRNA profile (if any detail is reported).

| **Family**/*Genus* | *Viroid species* | Host plant | Viroid siRNA profile* | Reference** |
| --- | --- | --- | --- | --- |
| **Avsunviroidae**  chloroplastic |  |  |  |  |
| *Pelamoviroid* | *Peach latent mosaic viroid* | Peach | 21nt,22nt;  both strands | Di Serio et al. 2009; Bolduc et al. 2010; Navarro et al. 2012; Zhang et al. 2014a; He et al. 2017; Lu et al. 2017 |
| *Pelamoviroid* | *Peach latent mosaic viroid* | *Prunus* sp. |  | Pecman et al. 2017 |
| *Pelamoviroid* tentative | *Apple hammerhead viroid*  tentative species | Apple | 21nt,22nt;  both strands | Zhang et al. 2014a |
| **Pospiviroidae**  nuclear |  |  |  |  |
| *Apscaviroid* | *Apple dimple fruit viroid* | Fig | 21nt,22nt,24nt;  both strands | Chiumenti et al. 2014 |
| *Apscaviroid* | *Apple scar skin viroid* | Apple | 22nt,21nt,24nt | Zhang et al. 2014a; Liang et al. 2015 |
| *Apscaviroid* | *Australian grapevine viroid* | Grapevine | 21nt,22nt,24nt;  both strands | Turco, Reynard, Pooggin in prep; Velasco et al. 2014; Ma et al. 2017; Vargas-Asencio et al. 2017a |
| *Apscaviroid* | *Citrus dwarfing viroid* | Grapefruit | 22nt,21nt,24nt;  both strands | Visser et al. 2017; Visser et al. 2016 |
| *Apscaviroid* | *Citrus dwarfing viroid* | Blood orange |  | Navarro et al. 2018 |
| *Apscaviroid* | *Citrus dwarfing viroid* | Sweet orange |  | Roy et al. 2013a |
| *Apscaviroid* | *Citrus viroid III =*  *Citrus dwarfing viroid* | Lemon |  | Loconsole et al. 2012a |
| *Apscaviroid* | *Citrus viroid VI* | Citron | 21nt,22nt,24nt | Barrero et al. 2017 |
| *Apscaviroid* | *Citrus viroid VI* | Lemon |  | Olmos,Varveri, et al. in prep |
| *Apscaviroid* | *Grapevine latent viroid*  tentative species | Grapevine | 21nt,24nt,22nt;  both strands | Zhang et al. 2014a; Turco, Reynard, Pooggin in prep; Rotunno et al. 2018 |
| *Apscaviroid* | *Grapevine yellow speckle viroid 1* | Grapevine | 21nt,24nt,22nt;  both strands | Navarro et al. 2009; Giampetruzzi et al. 2012; Alabi et al. 2012; Wu et al. 2012; Seguin et al. 2014; Velasco et al. 2014; Maliogka et al. 2015; Saldarelli et al. 2015; Eichmeier et al. 2016; Chiumenti et al. 2016a; Fan et al. 2016; Reynard et al. 2016; Ma et al. 2017; Bester et al. 2017a,b; Cretazzo et al. 2017; Cretazzo and Velasco 2017; Fan et al. 2017b; Czotter et al. 2018; Massart et al. 2018 |
| *Apscaviroid* | *Grapevine yellow speckle viroid 2* | Grapevine | 21nt,24nt,22nt;  both strands | Alabi et al. 2012; Ma et al. 2017; Fan et al. 2017b; Czotter et al. 2018 |
| *Cocadviroid* | *Hop latent viroid* | Hop | 22nt,21nt,24nt; both strands | Jakse et al. 2015 |
| *Cocadviroid* | *Citrus bark cracking viroid* | Hop | 22nt,24nt,21nt; both strands | Jakse et al. 2015 |
| *Cocadviroid* | *Citrus bark cracking viroid* | Lemon |  | Loconsole et al. 2012a |
| *Hostuviroid* | *Hop stunt viroid* | Abutilon |  | Verdin et al. 2017 |
| *Hostuviroid* | *Hop stunt viroid* | Chickpea | 21nt,22nt,24nt;  both strands | Pirovano et al. 2015, pers comm |
| *Hostuviroid* | *Hop stunt viroid* | Citron | 21nt,22nt,24nt | Barrero et al. 2017 |
| *Hostuviroid* | *Hop stunt viroid* | Cucumber | 21nt,22nt,24nt;  both strands | Martinez et al. 2010 |
| *Hostuviroid* | *Hop stunt viroid* | Blood orange |  | Navarro et al. 2018 |
| *Hostuviroid* | *Hop stunt viroid* | Grapevine | 21nt,24nt,22nt; both strands | Navarro et al. 2009; Giampetruzzi et al. 2012; Wu et al. 2012; Seguin et al. 2014; Velasco et al. 2014; Maliogka et al. 2015; Saldarelli et al. 2015; Eichmeier et al. 2016; Visser et al. 2016; Chiumenti et al. 2016a; Fan et al. 2016; Reynard et al. 2016; Ma et al. 2017; Bester et al. 2017a,b; Barrero et al. 2017; Cretazzo et al. 2017; Cretazzo and Velasco 2017; Fan et al. 2017b; Czotter et al. 2018; Massart et al. 2018; Ahmed et al. 2018 |
| *Hostuviroid* | *Hop stunt viroid* | Lemon | 21nt,22nt,24nt;  both strands | Su et al. 2015 |
| *Hostuviroid* | *Hop stunt viroid* | *N. benthamiana* | 22nt,21nt,24nt;  both strands | Castellano et al. 2015 |
| *Hostuviroid* | *Hop stunt viroid* | Peach |  | He et al. 2017 |
| *Hostuviroid* | *Hop stunt viroid* | Sweet cherry |  | Ruiz-Garcia et al. 2016 |
| *Pospiviroid* | *Citrus exocortis viroid* | Blood orange |  | Navarro et al. 2018 |
| *Pospiviroid* | *Citrus exocortis viroid* | Sweet orange |  | Roy et al. 2013a |
| *Pospiviroid* | *Citrus exocortis viroid* | Lemon |  | Olmos,Varveri, et al. in prep |
| *Pospiviroid* | *Citrus exocortis viroid* | Tomato |  | Xu et al. 2017 |
| *Pospiviroid* | *Columnea latent viroid* | Tomato |  | Pecman et al. 2017 |
| *Pospiviroid* | *Potato spindle tuber viroid* | *N. benthamiana* & rdr6i mutant | 22nt,21nt,24nt; (-) strand bias | Di Serio et al. 2010; Minoia et al. 2014; |
| *Pospiviroid* | *Potato spindle tuber viroid* | Tomato | 21nt,22nt,24nt; (-) strand bias; both strands | Li et al. 2012; Tsushima et al. 2015; Xu et al. 2017; Zheng et al. 2017c; Adkar-Purushothama et al. 2015; Adkar-Purushothama et al. 2018a,2018b |
| *Pospiviroid* | *Tomato apical stunt viroid* | Tomato |  | Pecman et al. 2017 |
| **Unassigned** | *Grapevine hammerhead viroid*-like RNA | Grapevine | 21nt,22nt;  both strands | Wu et al. 2012; Zhang et al. 2014a |
| **Unassigned** | *Mulberry viroid*-like RNA | Peony |  | Verdin et al. 2017 |
| **Unassigned**  endogenous | *Dianthus caryophyllus retroviroid*-like element | Carnation |  | Verdin et al. 2017 |

**References**

Adkar-Purushothama CR, Brosseau C, Giguère T, Sano T, Moffett P, Perreault JP. 2015. Small RNA Derived from the Virulence Modulating Region of the Potato spindle tuber viroid Silences callose synthase Genes of Tomato Plants. Plant Cell 27:2178-94. doi: 10.1105/tpc.15.00523.

Adkar-Purushothama CR, Perreault JP. 2018a. Alterations of the viroid regions that interact with the host defense genes attenuate viroid infection in host plant. RNA Biol. May 22:1-12. doi: 10.1080/15476286.2018.1462653.

Adkar-Purushothama CR, Sano T, Perreault JP. 2018b. Viroid derived small RNA induces early flowering in tomato plants by RNA silencing. Mol Plant Pathol. Jul 16. doi: 10.1111/mpp.12721.

Ahmed I, Fan XD, Zhang ZP, Ren F, Hu GJ, Li ZN, Khaskheli MI, Dong YF. 2018. First Report of Grapevine Syrah virus-1 in Grapevines in China. Plant Dis. 102:466. doi.org/10.1094/PDIS-09-17-1358-PDN.

Alabi OJ, Zheng Y, Jagadeeswaran G, Sunkar R, Naidu RA. 2012. High-throughput sequence analysis of small RNAs in grapevine (Vitis vinifera L.) affected by grapevine leafroll disease. Mol Plant Pathol. 13:1060-76. doi: 10.1111/j.1364-3703.2012.00815.x.

Barrero RA, Napier KR, Cunnington J, Liefting L, Keenan S, Frampton RA, Szabo T, Bulman S, Hunter A, Ward L, Whattam M, Bellgard M. 2017. An internet-based bioinformatics toolkit for plant biosecurity diagnosis and surveillance of viruses and viroids. BMC Bioinformatics. 18:26. doi: 10.1186/s12859-016-1428-4.

Bester R, Burger JT, Maree HJ. 2017a. Differential expression of miRNAs and associated gene targets in grapevine leafroll-associated virus 3-infected plants. Arch Virol. 162:987-996. doi: 10.1007/s00705-016-3197-9.

Bester, R., Burger, J.T., and Maree, H.J. 2017b. Transcriptome analysis reveals differentially expressed small RNAs and genes associated with grapevine leafroll-associated virus 3 infections. Physiol Mol Plant Pathol. 100:220-236. doi: 10.1016/j.pmpp.2017.10.006.

Bolduc F, Hoareau C, St-Pierre P, Perreault JP. 2010. In-depth sequencing of the siRNAs associated with peach latent mosaic viroid infection. BMC Mol Biol. 11:16. doi: 10.1186/1471-2199-11-16.

Castellano M, Martinez G, Pallás, Gómez G. 2015. Alterations in host DNA methylation in response to constitutive expression of Hop stunt viroid RNA in Nicotiana benthamiana plants. Plant Pathol. 64:1247-1257. doi: 10.1111/ppa.12358.

Chiumenti M, Torchetti EM, Di Serio F, Minafra A. 2014. Identification and characterization of a viroid resembling apple dimple fruit viroid in fig (Ficus carica L.) by next generation sequencing of small RNAs. Virus Res. 188:54-9. doi: 10.1016/j.virusres.2014.03.026.

Chiumenti M, Giampetruzzi A, Morelli M, Savino VN, Martelli GP, La Notte P, Palmisano F, Saldarelli P. 2016a. Detection and molecular characterization of a Grapevine Roditis leaf discoloration-associated virus (GRLDaV) variant in an autochthonous grape from Apulia (Italy). Virus Genes 52:428-31. doi: 10.1007/s11262-016-1305-2

Cretazzo E, Padilla CV, Velasco L. 2017. First Report of Grapevine Red Globe virus in grapevine in Spain. Plant Dis. 101:264. doi.org/10.1094/PDIS-06-16-0932-PDN.

Cretazzo E, Velasco L. 2017. High‐throughput sequencing allowed the completion of the genome of grapevine Red Globe virus and revealed recurring co‐infection with other tymoviruses in grapevine. Plant Pathol. 66:1202-1213. doi.org/10.1111/ppa.12669.

Czotter N, Molnar J, Szabó E, Demian E, Kontra L, Baksa I, Szittya G, Kocsis L, Deak T, Bisztray G, Tusnady GE, Burgyan J, Varallyay E. 2018. NGS of Virus-Derived Small RNAs as a Diagnostic Method Used to Determine Viromes of Hungarian Vineyards. Front Microbiol. 9:122. doi: 10.3389/fmicb.2018.00122.

Di Serio F, Gisel A, Navarro B, Delgado S, Martínez de Alba AE, Donvito G, Flores R. 2009. Deep sequencing of the small RNAs derived from two symptomatic variants of a chloroplastic viroid: implications for their genesis and for pathogenesis. PLoS One 4:e7539. doi: 10.1371/journal.pone.0007539.

Di Serio F, Martínez de Alba AE, Navarro B, Gisel A, Flores R. 2010. RNA-dependent RNA polymerase 6 delays accumulation and precludes meristem invasion of a viroid that replicates in the nucleus. J Virol. 84:2477-89. doi: 10.1128/JVI.02336-09.

Eichmeier A, Komínková M, Komínek P, Baránek M. 2016. Comprehensive Virus Detection Using Next Generation Sequencing in Grapevine Vascular Tissues of Plants Obtained from the Wine Regions of Bohemia and Moravia (Czech Republic). PLoS One 11:e0167966. doi: 10.1371/journal.pone.0167966.

Fan XD, Dong YF, Zhang ZP, Ren F, Hu GJ, Li ZN, Zhou J. 2016a. First Report of Grapevine red globe virus (GRGV) in Grapevines in China. Plant Dis. 100:2340. doi.org/10.1094/PDIS-05-16-0701-PDN.

Fan X, Hong N, Zhang Z, Yang Z, Ren F, Hu G, Li Z, Zhou J, Dong Y, Wang G. 2016b. Identification of a divergent variant of grapevine berry inner necrosis virus in grapevines showing chlorotic mottling and ring spot symptoms. Arch Virol. 161:2025-7. doi: 10.1007/s00705-016-2856-1.

Fan XD, Zhang ZP, Ren F, Hu GJ, Li ZN, Dong YF. 2017b. First Report of Grapevine geminivirus A from Grapevines in China. Plant Dis. 101:1333. doi.org/10.1094/PDIS-01-17-0106-PDN.

Giampetruzzi A, Roumi V, Roberto R, Malossini U, Yoshikawa N, La Notte P, Terlizzi F, Credi R, Saldarelli P. 2012. A new grapevine virus discovered by deep sequencing of virus- and viroid-derived small RNAs in Cv Pinot gris. Virus Res. 163:262-8. doi: 10.1016/j.virusres.2011.10.010.

He Y, Cai L, Zhou L, Yang Z, Hong N, Wang G, Li S, Xu W. 2017. Deep sequencing reveals the first fabavirus infecting peach. Sci Rep. 7:11329. doi: 10.1038/s41598-017-11743-7.

Jakse J, Radisek S, Pokorn T, Matousek J, Javornik B. 2015. Deep‐sequencing revealed Citrus bark cracking viroid (CBCVd) as a highly aggressive pathogen on hop. Plant Pathol. 64:831-842. doi: 10.1111/ppa.12325.

Katsarou K, Mavrothalassiti E, Dermauw W, Van Leeuwen T, Kalantidis K. 2016. Combined Activity of DCL2 and DCL3 Is Crucial in the Defense against Potato Spindle Tuber Viroid. PLoS Pathog. 12:e1005936. doi: 10.1371/journal.ppat.1005936.

Li R, Gao S, Hernandez AG, Wechter WP, Fei Z, Ling KS. 2012. Deep sequencing of small RNAs in tomato for virus and viroid identification and strain differentiation. PLoS One 7:e37127. doi: 10.1371/journal.pone.0037127.

Liang P, Navarro B, Zhang Z, Wang H, Lu M, Xiao H, Wu Q, Zhou X, Di Serio F, Li S. 2015. Identification and characterization of a novel geminivirus with a monopartite genome infecting apple trees. J Gen Virol. 96:2411-20. doi: 10.1099/vir.0.000173.

Loconsole G, Onelge N, Potere O, Giampetruzzi A, Bozan O, Satar S, De Stradis A, Savino V, Yokomi RK, Saponari M. 2012a. Identification and characterization of citrus yellow vein clearing virus, a putative new member of the genus Mandarivirus. Phytopathology 102:1168-75. doi: 10.1094/PHYTO-06-12-0140-R.

Lu MG, Zhang C, Zhang ZX, Wang CA, Li SF. 2017. Nectarine stem-pitting-associated virus Detected in Peach Trees in China. Plant Dis. 101:513. doi.org/10.1094/PDIS-09-16-1256-PDN.

Ma YX, Li SF, and Zhang ZX. 2017. First Report of Grapevine rupestris vein feathering virus in an Old Grapevine in China. Plant Dis. 101:848. doi.org/10.1094/PDIS-11-16-1620-PDN.

Maliogka VI, Olmos A, Pappi PG, Lotos L, Efthimiou K, Grammatikaki G, Candresse T, Katis NI, Avgelis AD. 2015. A novel grapevine badnavirus is associated with the Roditis leaf discoloration disease. Virus Res. 203:47-55. doi: 10.1016/j.virusres.2015.03.003.

Martinez G, Donaire L, Llave C, Pallas V, Gomez G. 2010. High-throughput sequencing of Hop stunt viroid-derived small RNAs from cucumber leaves and phloem. Mol Plant Pathol 11: 347–359.

Massart S, Chiumenti M, De Jonghe K, Glover R, Haegeman A, Koloniuk I, Komínek P, Kreuze J, Kutnjak D, Lotos L, Maclot F, Maliogka VI, Maree H, Olivier T, Olmos A, Pooggin M, Reynard JS, Ruiz-García AB, Safarova D, Schneeberger PH, Sela N, Turco S, Vainio EJ, Varallyai E, Verdin E, Westenberg M, Brostaux Y, Candresse T. 2018. Virus detection by high-throughput sequencing of small RNAs: large scale performance testing of sequence analysis strategies. Phytopathology. Aug 2. doi: 10.1094/PHYTO-02-18-0067-R.

Minoia S, Carbonell A, Di Serio F, Gisel A, Carrington JC, Navarro B, Flores R. 2014. Specific argonautes selectively bind small RNAs derived from potato spindle tuber viroid and attenuate viroid accumulation in vivo. J Virol. 88:11933-45. doi: 10.1128/JVI.01404-14.

Navarro B, Pantaleo V, Gisel A, Moxon S, Dalmay T, Bisztray G, Di Serio F, Burgyán J. 2009. Deep sequencing of viroid-derived small RNAs from grapevine provides new insights on the role of RNA silencing in plant-viroid interaction. PLoS One 4:e7686. doi: 10.1371/journal.pone.0007686.

Navarro B, Gisel A, Rodio ME, Delgado S, Flores R, Di Serio F. 2012. Small RNAs containing the pathogenic determinant of a chloroplast-replicating viroid guide the degradation of a host mRNA as predicted by RNA silencing. Plant J. 70:991-1003. doi: 10.1111/j.1365-313X.2012.04940.x.

Navarro B, Minutolo M, De Stradis A, Palmisano F, Alioto D, Di Serio F. 2018. The first phlebo-like virus infecting plants: a case study on the adaptation of negative-stranded RNA viruses to new hosts. Mol Plant Pathol. 19:1075-1089. doi: 10.1111/mpp.12587.

Pecman A, Kutnjak D, Gutiérrez-Aguirre I, Adams I, Fox A, Boonham N, Ravnikar M. 2017. Next Generation Sequencing for Detection and Discovery of Plant Viruses and Viroids: Comparison of Two Approaches. Front Microbiol. 8:1998. doi: 10.3389/fmicb.2017.01998.

Pirovano W, Miozzi L, Boetzer M, Pantaleo V. 2015. Bioinformatics approaches for viral metagenomics in plants using short RNAs: model case of study and application to a Cicer arietinum population. Front Microbiol. 5:790. doi: 10.3389/fmicb.2014.00790.

Reynard JS, Schumacher S, Menzel W, Fuchs J, Bohnert P, Glasa M, Wetzel T, Fuchs R. 2016. First Report of Grapevine Pinot gris virus in German Vineyards. Plant Dis. 100:2545. doi.org/10.1094/PDIS-07-16-0966-PDN.

Rotunno S, Vaira AM, Marian D, Schneider A, Raimondi S, Di Serio F, Navarro B, Miozzi L. 2018. First Report of Grapevine Latent Viroid Infecting Grapevine (Vitis vinifera L.) in Italy. Plant Disease 102:1672. doi: doi.org/10.1094/PDIS-01-18-0076-PDN.

Roy A, Choudhary N, Guillermo LM, Shao J, Govindarajulu A, Achor D, Wei G, Picton DD, Levy L, Nakhla MK, Hartung JS, Brlansky RH. 2013a. A novel virus of the genus Cilevirus causing symptoms similar to citrus leprosis. Pytopathology 103:488-500. doi: 10.1094/PHYTO-07-12-0177-R.

Ruiz-Garcia AB, Martinez C, Santiago R, Garcia MT, de Prado N, Olmos A. 2016. First report of Little cherry virus 1 (LChV-1) in sweet cherry in Spain. Plant Dis. 100:2340. doi: doi.org/10.1094/PDIS-05-16-0620-PDN.

Saldarelli P, Giampetruzzi A, Morelli M, Malossini U, Pirolo C, Bianchedi P, Gualandri V. 2015. Genetic Variability of Grapevine Pinot gris virus and Its Association with Grapevine Leaf Mottling and Deformation. Phytopathology 105:555-563. doi.org/10.1094/PHYTO-09-14-0241-R.

Seguin J, Rajeswaran R, Malpica-López N, Martin RR, Kasschau K, Dolja VV, Otten P, Farinelli L, Pooggin MM. 2014. De novo reconstruction of consensus master genomes of plant RNA and DNA viruses from siRNAs. PLoS One 9:e88513. doi: 10.1371/journal.pone.0088513.

Su X, Fu S, Qian Y, Xu Y, Zhou X. 2015. Identification of Hop stunt viroid infecting Citrus limon in China using small RNAs deep sequencing approach. Virol J. 12:103. doi: 10.1186/s12985-015-0332-2.

Tsushima D, Adkar-Purushothama CR, Taneda A et al. 2015. Changes in relative expression levels of viroid-specific small RNAs and microRNAs in tomato plants infected with severe and mild symptom-inducing isolates of Potato spindle tuber viroid. J Gen Plant Pathol. 81:49. doi.org/10.1007/s10327-014-0566-7.

Vargas-Asencio J, Perry KL, Wise A, Fuchs M. 2017a. Detection of Australian grapevine viroid in Vitis vinifera in New York. Plant Dis. 101:848.

Velasco L, Bota J, Montero R, and Cretazzo E. 2014. Differences of Three Ampeloviruses' Multiplication in Plant May Explain Their Incidences in Vineyards. Plant Dis. 98:395-400. doi.org/10.1094/PDIS-04-13-0433-RE.

Verdin E, Wipf-Scheibel C, Gognalons P, Aller F, Jacquemond M, Tepfer M. 2017. Sequencing viral siRNAs to identify previously undescribed viruses and viroids in a panel of ornamental plant samples structured as a matrix of pools. Virus Res. 241:19-28. doi: 10.1016/j.virusres.2017.05.019.

Visser M, Bester R, Burger JT, Maree HJ. 2016. Next-generation sequencing for virus detection: covering all the bases. Virol J. 13:85. doi: 10.1186/s12985-016-0539-x.

Visser M, Cook G, Burger JT, Maree HJ. 2017. In silico analysis of the grapefruit sRNAome, transcriptome and gene regulation in response to CTV-CDVd co-infection. Virol J. 14:200. doi: 10.1186/s12985-017-0871-9.

Wu Q, Wang Y, Cao M, Pantaleo V, Burgyan J, Li WX, Ding SW. 2012. Homology-independent discovery of replicating pathogenic circular RNAs by deep sequencing and a new computational algorithm. Proc Natl Acad Sci U S A. Mar 6;109(10):3938-43. doi: 10.1073/pnas.1117815109.

Xu C, Sun X, Taylor A, Jiao C, Xu Y, Cai X, Wang X, Ge C, Pan G, Wang Q, Fei Z, Wang Q. 2017. Diversity, Distribution, and Evolution of Tomato Viruses in China Uncovered by Small RNA Sequencing. J Virol. 91(11). pii: e00173-17. doi: 10.1128/JVI.00173-17

Zhang Z, Qi S, Tang N, Zhang X, Chen S, Zhu P, Ma L, Cheng J, Xu Y, Lu M, Wang H, Ding SW, Li S, Wu Q. 2014a. Discovery of replicating circular RNAs by RNA-seq and computational algorithms. PLoS Pathog. 10:e1004553. doi: 10.1371/journal.ppat.1004553.

Zheng Y, Wang Y, Ding B, Fei Z. 2017c. Comprehensive Transcriptome Analyses Reveal that Potato Spindle Tuber Viroid Triggers Genome-Wide Changes in Alternative Splicing, Inducible trans-Acting Activity of Phased Secondary Small Interfering RNAs, and Immune Responses. J Virol. 91:e00247-17. doi: 10.1128/JVI.00247-17.
