# Supplementary material for: Small RNA-Omics for Plant Virus Identification, Virome Reconstruction, and Antiviral Defense Characterization
Source: Front Microbiol. 2018 Nov 20;9:2779. doi: 10.3389/fmicb.2018.02779 (PMC6256188; doi:10.3389/fmicb.2018.02779)
Supplement: Supplementary file 3 [file Table_3.DOCX]

**List S1. Plant virus/viroid/satellite Families/Orders/Groups/Genera analysed by sRNA-seq (highlighted in blue) and Genera not yet analyzed by small RNA-seq (highlighted in red)**

(for details of viral species and host plants, see Table S1, Table S2, List S2 and ICTV Virus Taxonomy: 2017 Release, <https://talk.ictvonline.org/taxonomy/>)

**1. Alphaflexiviridae, order Tymovirales, group IV, (+)ssRNA:** 7 Genera incl **5 plant genera**: ***Allexivirus*** (12 Species), ***Lolavirus*** (1 Species), ***Mandarivirus*** (2 Species), ***Potexvirus*** (38 Species)

**2. Alphasatellitidae, order Unassigned, group II, ssDNA satellite:** 2 Subfamiles **Geminialphasatellitinae 5 Genera**: Ageyesisatellite (2 Species), Clecrusatellite (8 Species), Colecusatellite (25 Species), Gosmusatellite (6 Species) and unassigned genus; and **Nanoalphasatellitinae 7 Genera**: Babusatellite (4 Species), Clostunsatellite (5 Species), Fabenesatellite (1 Species), Milvetsatellite (1 Species), Mivedwarsatellite (4 Species), Sophoyesatellite (1 Species), Subclovsatellite (3 Species) and unassigned genus (1 Species)

**3. Amalgaviridae, order Unassigned, group III, dsRNA: 1 genus *Amalgavirus*** (4 Species)

**4. Aspiviridae** (former Ophioviridae)**, order Unassigned, group III, (-)ssRNA: 1 genus** ***Ophiovirus*** (7 Species)

**5. Avsunviroidae, order Unassigned, viroids: 3 genera** ***Avsunviroid*** (1 Species), ***Elaviroid*** (1 Species), ***Pelamoviroid*** (2 Species)

**6. Benyviridae, order Unassigned, group IV, (+)ssRNA: 1 genus** ***Benyvirus*** (3 Species)

**7. Betaflexiviridae, order Tymovirales, group IV, (+)ssRNA:** Subfamily **Quinvirinae** **4 Genera**: ***Carlavirus*** (53 Species), ***Foveavirus*** (7 Species), ***Robigovirus*** (5 Species) and ***Unassigned*** genus (3 Species); and Subfamily **Trivirinae** **8 Genera:** ***Capillovirus*** (2 Species), ***Chordovirus*** (2 Species), ***Citrivirus*** (1 Species), ***Divavirus*** (3 Species), ***Prunevirus*** (2 Species), ***Tepovirus*** (2 Species), ***Trichovirus*** (7 Species), ***Vitivirus*** (10 Species)

**8. Bromoviridae, order Unassigned, group IV, (+)ssRNA: 6 genera** ***Alfamovirus*** (1 Species), ***Anulavirus*** (2 Species), ***Bromovirus*** (6 Species), ***Cucumovirus*** (4 Species), ***Ilarvirus*** (22 Species), ***Oleavirus*** (1 Species)

**9. Caulimoviridae, order Ortervirales**, **group VII, dsDNA-RT:**  **8 genera** ***Badnavirus*** (46 Species), ***Caulimovirus*** (11 Species), ***Cavemovirus*** (2 Species), ***Petuvirus*** (1 Species), ***Rosadnavirus*** (1 species), ***Solendovirus*** (2 Species), ***Soymovirus*** (4 species), ***Tungrovirus*** (1 Species) and **tentative genus** of endogenous viral elements ***Florendovirus*** (many species)

**10. Closteroviridae, order Unassigned, group IV, (+)ssRNA: 4 genera** ***Ampelovirus*** (10 Species), ***Closterovirus*** (13 Species), ***Crinivirus*** (14 Species), ***Velarivirus*** (7 Species) and unassigned genus

**11. Endornaviridae, order Unassigned, group III, dsRNA:** 2 genera incl **1 plant genus** ***Alphaendornavirus*** (19 Species)

**12. Fimoviridae, order Bunyavirales; group V, (-)ssRNA: 1 genus** ***Emaravirus*** (9 Species)

**13. Geminiviridae, order Unassigned, group II, ssDNA: 9 Genera**: ***Becurtovirus*** (2 Species), ***Begomovirus*** (388 Species), ***Capulavirus*** (4 Species), ***Curtovirus*** (3 Species), ***Eragrovirus*** (1 Species), ***Grablovirus*** (1 Species), ***Mastrevirus*** (37 Species), ***Topocuvirus*** (1 Species), ***Turncurtovirus*** (2 Species) and ***Unassigned*** genus (2 Species)

**14. Luteoviridae, order Unassigned, group IV, (+)ssRNA: 3 genera** ***Enamovirus*** (2 Species), ***Luteovirus*** (8 Species), ***Polerovirus*** (19 Species) and *unassigned* genus (7 Species)

**15. Metaviridae, order Ortervirales, group VI, ssRNA-RT:** 2 genera incl **1 plant genus *Metavirus*** (3 plant species) of **Ty3/Gypsy LTR retrotransposons**

**16. Nanoviridae, order Unassigned, group II, ssDNA: 2 Genera**: ***Babuvirus*** (3 Species), ***Nanovirus*** (8 Species) and ***unassigned*** genus (1 Species)

**17. Partitiviridae, order Unassigned, group III, dsRNA:** 5 genera incl **3 plant genera** ***Alphapartitivirus*** (14 Species), ***Betapartitivirus*** (17 Species), ***Deltapartitivirus*** (5 Species) and ***unassigned*** genus (15 Species)

**18. Phenuiviridae, order Bunyavirales; group V, (-)ssRNA:** 4 genera incl **1 plant genus** ***Tenuivirus*** (9 Species) and 1 genus ***unassigned*** (2 Species)

**19. Pospiviroidae, order Unassigned, viroids: 5 genera** ***Apscaviroid*** (10 Species), ***Cocadviroid*** (4 Species), ***Coleviroid*** (3 Species), ***Hostuviroid*** (2 Species), ***Pospiviroid*** (9 Species)

**20. Potyviridae, order Unassigned, group IV, (+)ssRNA: 10 genera** ***Bevemovirus*** (1 Species), ***Brambyvirus*** (1 Species), ***Bymovirus*** (6 Species), ***Ipomovirus*** (7 Species), ***Macluravirus*** (8 Species), ***Poacevirus*** (3 Species), ***Potyvirus*** (168 Species), ***Roymovirus*** (1 Species), ***Rymovirus*** (3 Species), ***Tritimovirus*** (6 Species) and unassigned genus (2 Species)

**21. Pseudoviridae, order Ortervirales, group VI, ssRNA-RT:** 3 genera incl **2 plant genera *Pseudovirus,*** ***Sirevirus*** and ***unassigned* genus** = plant **Ty1/Copia LTR retrotransposons** (LTR-transposon siRNAs: e.g., Sun et al. 2013; Alejandri-Ramírez et al. 2018; Wang et al. 2018a)

**22. Reoviridae, order Unassigned, group III, dsRNA:** 2 subfamilies **Sedoreovirinae** 6 genera incl **1 plant genus** ***Phytoreovirus*** (3 Species incl 2 plant species) and **Spinareovirinae** 9 genera incl ***2 plant genera*** ***Fijivirus*** (9 Species), ***Oryzavirus*** (2 Species) and 1 unassigned

**23. Rhabdoviridae, order Mononegavirales; group V (-)ssRNA:** 18 genera including 4 “plant” genera ***Cytorhabdovirus*** (11 Species)***, Dichorhavirus*** (2 Species)***, Nucleorhabdovirus*** (10 Species)***, Varicosavirus*** (1 species)

**24. Secoviridae, order Picornavirales, group IV, (+)ssRNA:** 1 Subfamily Comovirinae

(**3 Genera**: ***Comovirus, Fabavirus, Nepovirus***), and **5 Genera**: ***Cheravirus*** (5 Species), **Sadwavirus** (1 Species), ***Sequivirus*** (3 Species), ***Torradovirus*** (6 Species), ***Waikavirus*** (4 Species), and ***unassigned*** **genus** (5 species)

**25. Solemoviridae, order Unassigned, group IV, (+)ssRNA: 2 genera** ***Polemovirus*** (1 Species) and ***Sobemovirus*** (19 Species)

**26. Tolecusatellitidae, order Unassigned, ssDNA satellite: 2 genera** ***Betasatellite*** (61 Species) and ***Deltasatellite*** (11 Species)

**27. Tombusviridae, order Unassigned, group IV, (+)ssRNA: 16 genera** ***Alphacarmovirus*** (7 Species), ***Alphanecrovirus*** (3 Species), ***Aureusvirus*** (5 Species), ***Avenavirus*** (1 Species), ***Betacarmovirus*** (4 Species), ***Betanecrovirus*** (3 Species), ***Dianthovirus*** (3 Species), ***Gallantivirus*** (1 Species), ***Gammacarmovirus*** (4 Species), ***Macanavirus*** (1 Species), ***Machlomovirus*** (1 Species), ***Panicovirus*** (3 Species), ***Pelarspovirus*** (5 Species), ***Tombusvirus*** (17 Species), ***Umbravirus*** (9 Species), ***Zeavirus*** (1 Species) and Unassigned (6 Species)

**28. Tospoviridae** formerly Bunyaviridae**, order Bunyavirales; group V, (-)ssRNA:** 1 genus ***Orthotospovirus***/formerly *Tospovirus* (11 species)

**29. Tymoviridae, order Tymovirales, group IV, (+)ssRNA: 3 genera** ***Maculavirus*** (1 Species), ***Marafivirus*** (10 Species), ***Tymovirus*** (28 Species) and unassigned genus (1 species)

**30. Virgaviridae, order Unassigned, group IV, (+)ssRNA: 7 genera** ***Furovirus*** (6 Species), ***Goravirus*** (2 Species), ***Hordeivirus*** (4 Species), ***Pecluvirus*** (2 Species), ***Pomovirus*** (5 Species), ***Tobamovirus*** (37 Species), ***Tobravirus*** (3 Species)

**31. Unassigned family**

16 Genera incl **9 plant genera**: ***Albetovirus*** **(+)ssRNA satellite** of **Tobmusviridae** (3 Species), ***Aumaivirus*** **(+)ssRNA satellite** of **Tobmusviridae** (1 Species), ***Blunervirus* (+)ssRNA** (1 Species), ***Cilevirus*** **(+)ssRNA** (2 Species), ***Higrevirus*** **(+)ssRNA** (1 Species), ***Idaeovirus*** (2 Species), ***Ourmiavirus* (+)ssRNA** (3 Species), ***Papanivirus*** **(+)ssRNA satellite** of **Tobmusviridae** (1 Species), ***Virtovirus*** **(+)ssRNA satellite** of **Virgaviridae** (1 Species), and ***Unassigned*** **(+)ssRNA satellite** of **Bromoviridae *Cucumovirus*** Cucumber mosaic virus, ***Unassigned*** **(+)ssRNA satellite** of **Secoviridae *Nepovirus*** Grapevine fanleaf virus, ***Unassigned*** **(+)ssRNA satellite** of **Tobmusviridae *Betanecrovirus*** Beet black scorch virus, ***Unassigned*** **(+)ssRNA satellite** of **Virgaviridae *Tobamovirus*** Bamboo mosaic virus, ***Unassigned*** **(+)ssRNA satellite** (of unknown helper virus) Grapevine satellite virus
