# Supplementary material for: Small RNA-Omics for Plant Virus Identification, Virome Reconstruction, and Antiviral Defense Characterization
Source: Front Microbiol. 2018 Nov 20;9:2779. doi: 10.3389/fmicb.2018.02779 (PMC6256188; doi:10.3389/fmicb.2018.02779)
Supplement: Supplementary file 4 [file Table_4.docx]

**List S2. Host plant Families/**Orders**/**Sub-clades/**Clades**/**Species** for which **virus/viroid/satellite**-derived siRNAs were analyzed by small RNA sequencing

**1. Actinidiaceae** order Ericales, clade Asterids, clade Eudicots, clade **Angiosperms**, kingdom Plantae

***Actinidia sp.***: **Betaflexiviridae** *Vitivirus* Actinidia virus B

**Kiwifruit *Actinidia chinensis***: **Betaflexiviridae** *Capillovirus* Apple stem grooving virus *Vitivirus* Actinidia virus A; **Fimoviridae** *Emaravirus* Actinidia chlorotic ringspot-associated virus

**2. Alstroemeriaceae** order Liliales, clade Monocots, clade **Angiosperms**, kingdom Plantae

**Alstroemeria *Alstroemeria sp.***: **Betaflexiviridae** *Carlavirus* Lily symptomless virus; **Potyviridae** *Potyvirus* Alstroemeria mosaic virus Unassigned virus

**3. Amaranthaceae** order Caryophyllales, clade Eudicots, clade **Angiosperms**, kingdom Plantae

***Beta macrocarpa***: **Benyviridae** *Benyvirus* Beet necrotic yellow vein virus

**4. Amaryllidaceae** order Asparagales, clade Monocots, clade **Angiosperms**, kingdom Plantae

**Onion *Allium cepa***: **Tospoviridae** *Orthotospovirus* Iris yellow spot virus

**5. Amborellaceae** order Amborellales, clade **Angiosperms**, kingdom Plantae

**Amborella *Amborella trichopoda***: **Caulimoviridae** *Florendovirus* endogenous Amborella trichopoda A virus Amborella trichopoda B virus Amborella trichopoda C virus

**6. Apiaceae** order Apiales, clade Asterids, clade Eudicots, clade **Angiosperms**, kingdom Plantae

**Common hedge parsley *Torilis arvensis***: **Luteoviridae** *Polerovirus-*like*;* **Potyviridae** *Potyvirus* Carrot thin leaf virus**; Secoviridae** *Torradovirus* Carrot torrado virus 1

**Parsley *Petroselinum crispum***: **Potyviridae** *Potyvirus* Apium virus Y Carrot thin leaf virus

**7. Apocynaceae** order Gentianales, clade Asterids, clade Eudicots, clade **Angiosperms**, kingdom Plantae

**Milkweed *Asclepias sp***.: **Caulimoviridae** Endogenous *Petuvirus*-like

**Periwinkle**: **Luteoviridae** *Polerovirus,* **Endornaviridae** Endornavirus

**8. Araceae** order Alismatales, clade Monocots, clade **Angiosperms**, kingdom Plantae

**Areca palm** ***Areca catechu***: **Potyviridae** *Unassigned* Areca palm necrotic ringspot disease

9. **Arecaceae** order Arecales, clade Monocots, clade **Angiosperms**, kingdom Plantae

**Taro** ***Colocasia esculenta***:

**9. Asparagaceae** order Asparagales, clade Monocots, clade **Angiosperms**, kingdom Plantae

***Host*** sp: **Alphaflexiviridae** *Potexvirus* Hosta virus X

**10. Asteraceae** order Asterales, clade Asterids, clade Eudicots, clade **Angiosperms**, kingdom Plantae

***Cineraria sp***.: **Tospoviridae** *Orthotospovirus* Tomato spotted wilt virus

**Daisy *Bellis perennis***: **Bromoviridae** *Ilarvirus*

**Lettuce *Lactuca sativa***: **Aspiviridae** *Ophiovirus* Miraﬁori lettuce virus; **Closteroviridae** *Crinivirus* Beet pseudo yellows virus Lettuce infectious yellows virus; **Potyviridae** *Potyvirus* Endive necrotic mosaic virus Lettuce Italian necrotic virus; **Rhabdoviridae** *Varicosavirus* Lettuce big-vein associated virus

**Sunflower *Helianthus annuus***: **Endornaviridae** *Alphaendornavirus* Helianthus annuus alphaendornavirus

**Woolly burdock *Arctium tomentosum***: **Alphaflexiviridae** *Potexvirus* Alstroemeria virus X, **Fimoviridae** *Emaravirus* Woolly burdock yellow vein virus.

**11. Brassicaceae** order Brassicales, clade Rosids, clade Eudicots, clade **Angiosperms**, kingdom Plantae

***Arabidopsis thaliana****:* **Alphaflexiviridae** *Potexvirus* Bamboo mosaic virus, Bamboo mosaic virus **satellite** RNA; **Bromoviridae** *Cucumovirus* Cucumber mosaic virus; **Caulimoviridae** *Caulimovirus* Cauliflower mosaic virus; **Geminiviridae** *Begomovirus* Cabbage leaf curl virus; **Potyviridae** *Potyvirus* Turnip mosaic virus; **Tombusviridae** *Betacarmovirus* Turnip crinkle virus; **Virgaviridae** *Tobamovirus* Tobacco mosaic virus Youcai mosaic virus, *Tobravirus* Tobacco rattle virus; **Unassigned** (+)ssRNA *Cilevirus* Citrus leprosis virus cytoplasmic type 2

***Brassica oleracea****:* **Caulimoviridae** *Caulimovirus* Cauliflower mosaic virus;

***Brassica juncea****:* **Potyviridae** *Potyvirus* Turnip mosaic virus; **Tymoviridae** *Tymovirus* Turnip yellow mosaic virus

**12. Cannabaceae** order Rosales, clade Rosids, clade Eudicots, clade **Angiosperms**, kingdom Plantae

**Hop *Humulus lupulus***: **Betaflexiviridae** *Carlavirus* Hop latent virus Hop mosaic virus; **Pospiviroidae** *Cocadviroid* Hop latent viroid Citrus bark cracking viroid

**13. Caprifoliaceae** order Dipsacales, clade Asterids, clade Eudicots, clade **Angiosperms**, kingdom Plantae

**Centranthus *Centranthus sp.***: **Caulimoviridae** Caulimovirus endogenous ?

**14. Caricaceae** order Brassicales, clade Rosids, clade Eudicots, clade **Angiosperms**, kingdom Plantae

**Papaya *Carica papaya****:* **Potyviridae** *Potyvirus* Papaya leaf distortion mosaic virus Papaya ringspot virus

**15. Caryophyllaceae** order Caryophyllales, clade Eudicots, clade **Angiosperms**, kingdom Plantae

**Carnation *Dianthus caryophyllus****:* **Partitiviridae** *Deltapartitivirus;* **Unassigned** Endogenous Dianthus caryophyllus **retroviroid**-like element

***Silene latifolium***: **Caulimoviridae** Endogenous

**16. Celastraceae** order Celastrales, clade Rosids, clade Eudicots, clade **Angiosperms**, kingdom Plantae

***Euonymus bungeanus***: **Alphaflexiviridae** *Potexvirus* Euonymus yellow vein associated virus

**17. Characeae** order Charales class Charophyceae, **division** **Charophyta**, kingdom Plantae

**Green algae *Chara coralline***: **Caulimoviridae** Endogenous

**18. Cleomaceae**, order Brassicales, clade Rosids, clade Eudicots, clade **Angiosperms**, kingdom Plantae

**Cleome**: **Alphaflexiviridae** *Potexvirus* Tulip virus X; **Secoviridae** *Nepovirus* Tobacco ringspot virus; **Virgaviridae** *Tobravirus* Tobacco rattle virus

**19. Convolvulaceae** order Solanales, clade Asterids, clade Eudicots, clade **Angiosperms**, kingdom Plantae

**Sweet potato *Ipomoea batatas***: **Betaflexiviridae** *Carlavirus* Sweet potato C6 virus Sweet potato chlorotic fleck virus; **Caulimoviridae** *Cavemovirus* Sweet potato collusive virus, *Badnavirus* Sweet potato badnavirus A Sweet potato badnavirus B Sweet potato badnavirus C Sweet potato pakakuy virus; **Closteroviridae** *Crinivirus* Sweet potato chlorotic stunt virus; **Geminiviridae** *Begomovirus* Sweet potato golden vein associated virus Sweet potato leaf curl virus Sweet potato leaf curl Canary virus Sweet potato leaf curl Georgia virus Sweet potato leaf curl Sao Paulo virus Sweet potato leaf curl Spain virus Sweet potato leaf curl Uganda virus Sweet potato mosaic virus Sweet potato begomovirus, *Mastrevirus* Sweet potato symptomless mastrevirus 1; **Potyviridae** *Ipomovirus* Sweet potato mild mottle virus, *Potyvirus* Sweet potato feathery mottle virus Sweet potato virus 2 Sweet potato virus C Sweet potato virus G

**20. Cucurbitaceae** order Cucurbitales, clade Rosids, clade Eudicots, clade **Angiosperms**, kingdom Plantae

**Calabash *Lagenaria siceraria***: **Virgaviridae** *Tobamovirus* Cucumber green mottle mosaic virus

**Cucumber *Cucumis sativus***: **Bromoviridae** *Ilarvirus* Prunus necrotic ringspot virus; **Potyviridae** Ipomovirus Cucumber vein yellowing virus*, Potyvirus* Zucchini yellow mosaic virus; **Virgaviridae** *Tobamovirus* Cucumber green mottle mosaic virus; **Pospiviroidae** *Hostuviroid* Hop stunt viroid

**Melon**: **Tombusviridae** *Gammacarmovirus* Melon necrotic spot virus; **Potyviridae** *Potyvirus* Watermelon mosaic virus Zucchini yellow mosaic virus

**Pumpkin *Cucurbita moschata***: **Luteoviridae** *Polerovirus* Cucurbit aphid-borne yellows virus Melon aphid-borne yellows virus; **Potyviridae** *Potyvirus* Japanese yam mosaic virus Soybean mosaic virus Watermelon mosaic virus Zucchini yellow mosaic virus; **Virgaviridae** *Tobamovirus* Tobacco mild green mosaic virus Tobacco mosaic virus

**Squash *Cucurbita sp.***: **Geminiviridae** *Begomovirus* Squash leaf curl virus; **Secoviridae** *Torradovirus*-like

**Watermelon *Citrullus lanatus***: **Amalgaviridae** *Amalgavirus* Watermelon amalgavirus 1; **Betaflexiviridae** *Unassigned* Watermelon virus A; **Caulimoviridae** Endogenous *Cavemo*-*Badna*-like; **Geminiviridae** *Mastrevirus* Chickpea chlorotic dwarf virus; **Partitiviridae** *Deltapartitivirus* Citrullus lanatus cryptic virus; **Phenuiviridae** *Unclassified* Bunyavirales Watermelon crinkle leaf-associated virus 1 Watermelon crinkle leaf-associated virus 2; **Potyviridae** *Ipomovirus* Squash vein yellowing virus *Potyvirus* Watermelon mosaic virus Zucchini yellow mosaic virus

**Zucchini *Cucurbita pepo***: **Potyviridae** *Potyvirus* Watermelon mosaic virus; **Secoviridae** *Torradovirus* Squash chlorotic leaf spot virus; **Tospoviridae** *Orthotospovirus* Capsicum chlorosis virus

**21. Crassulaceae** order Saxifragales, clade Eudicots, clade **Angiosperms**, kingdom Plantae

**Kalanchoe *Kalanchoe sp.***: **Potyviridae** *Potyvirus* Kalanchoe mosaic virus; **Rhabdoviridae** rhabdovirus

**22. Cycadaceae** order Cycadales class Cycadopsida, **division Cycadophyta**, kingdom Plantae

**Queen sago palm *Cycas rumphii***: **Caulimoviridae** Endogenous

**23. Dioscoreaceae** order Dioscoreales, clade Monocots, clade **Angiosperms**, kingdom Plantae

**Water yam *Dioscorea alata***: **Geminiviridae** *Begomovirus* endogenous Endogenous geminivirus-like element 1

**24. Ebenaceae**, order Ericales, clade Asterids, clade Eudicots, clade **Angiosperms**, kingdom Plantae

**Japanese persimmon *Dyospiros kaki***: **Partitiviridae** putative *Deltapartitivirus* Persimmon cryptic virus

**25. Euphorbiaceae**, order Malpighiales, clade Rosids, clade Eudicots, clade **Angiosperms**, kingdom Plantae

**Cassava *Manihot esculenta***: **Alphaflexiviridae** *Potexvirus* Cassava new alphaflexivirus; **Geminiviridae** *Begomovirus* African cassava mosaic virus East African cassava mosaic Indian cassava mosaic virus South African cassava mosaic virus Sri Lankan cassava mosaic virus; **Luteoviridae** *Polerovirus* Cassava polero-like virus; **Potyviridae** *Ipomovirus* Cassava brown streak viruses; **Reovirirdae** *Unassigned* Cassava frogskin associated virus; **Secoviridae** *Torradovirus* Cassava torrado-like virus

**26. Fabaceae** order Fabales, clade Rosids, clade Eudicots, clade **Angiosperms**, kingdom Plantae

**Chickpea *Cicer arietinum***: **Tombusviridae** *Betacarmovirus* Turnip crinkle virus; **Virgaviridae** *Tobamovirus* Tomato mottle mosaic virus; **Pospiviroidae** *Hostuviroid* Hop stunt viroid

**Common bean** ***Phaseolus vulgaris***: **Endornaviridae** *Alphaendornavirus* Phaseolus vulgaris endornavirus 1 Phaseolus vulgaris endornavirus 2; **Betaflexiviridae** *Carlavirus* Cowpea mild mottle virus; **Bromoviridae** *Cucumovirus* Cucumber mosaic virus; **Caulimoviridae** *Caulimovirus*; **Closteroviridae** *Crinivirus* Bean yellow disorder virus; **Geminiviridae** *Begomovirus*; **Potyviridae** *Potyvirus* Cowpea aphid-borne mosaic virus Bean common mosaic virus Bean common mosaic necrosis virus Peanut mottle virus; **Rhabdoviridae** *Cytorhabdovirus*; **Solemoviridae** *Sobemovirus* Southern bean mosaic virus; **Tombusviridae** *Umbravirus* Tobacco mottle virus Unassigned

**Faba bean** ***Vicia faba***: **Nanoviridae** *Nanovirus* Faba bean necrotic stunt virus

**Green bean** ***Phaseolus sp.***: **Closteroviridae** *Crinivirus* Lettuce chlorosis virus

**Pagoda tree *Styphnolobium japonicum***: **Caulimoviridae** *Badnavirus* Pagoda yellow mosaic associated virus

**Pea *Pisum sativum***: **Nanoviridae** *Nanovirus* Pea necrotic yellow dwarf virus;

**Peanut *Arachis hypogaea***: **Tospoviridae** *Orthotospovirus* Tomato spotted wilt virus

**Pigeonpea** ***Cajanus cajan***: **Fimoviridae** *Emaravirus* Pigeonpea sterility mosaic virus-I Pigeonpea sterility mosaic virus-II; **Geminiviridae** *Begomovirus* Mungbean yellow mosaic India virus

**Soybean *Glycine max***: **Bromoviridae** *Cucumovirus* endogenous Cucumber mosaic virus RNA1; **Secoviridae** *Comovirus* Bean pod mottle virus

**Wild sweetpea *Sphenostylis angustifolia***: **Bromoviridae** *Ilarvirus*; **Luteoviridae** *Enamovirus* Pea enation virus, *Luteovirus*

**Yam bean** ***Pachyrhizus erosus***: **Potyviridae** *Potyvirus* Yam bean mosaic virus

**27. Funariaceae** order Funariales class Bryopsida, **division** **Bryophyta**, kingdom Plantae

**Moss *Physcomitrella patens***: **Caulimoviridae** Endogenous ?

**28. Gentianaceae** order Gentianales, clade Asterids, clade Eudicots, clade **Angiosperms**, kingdom Plantae

**Lisianthus *Eustoma sp***: **Rhabdoviridae** *Nucleorhabdovirus* Eggplant mottled dwarf virus

**29. Hydrangeaceae** order Cornales, clade Asterids, clade Eudicots, clade **Angiosperms**, kingdom Plantae

**Hydrangea** ***Hydrangea sp.***: **Potyviridae** *Potyvirus*

**30. Iridaceae** order Asparagales, clade Monocots, clade **Angiosperms**, kingdom Plantae

**Iris** ***Iris sp.***: **Luteoviridae** *Polerovirus*; **Potyviridae** *Potyvirus* Iris mild mosaic virus Iris severe mosaic virus Ornithogalum mosaic virus

**31. Juglandaceae** order Fagales, clade Rosids, clade Eudicots, clade **Angiosperms**, kingdom Plantae

**Pecan** ***Carya illinoinensis***: **Potyviridae** *Potyvirus* Pecan mosaic-associated virus

**32. Liliaceae** order Liliales, clade Monocots, clade **Angiosperms**, kingdom Plantae

***Fritillaria imperialis***: **Caulimoviridae** Endogenous *Petuvirus*-like Fritillaria imperialis endogenous pararetrovirus

**Lily** ***Lilium sp.***: **Betaflexiviridae** *Carlavirus* Lily symptomless virus; **Bromoviridae** *Cucumovirus* Cucumber mosaic virus; **Potyviridae** *Potyvirus* Lily yellow mosaic virus

**33. Lythraceae** order Myrtales, clade Rosids, clade Eudicots, clade **Angiosperms**, kingdom Plantae

**Crepe myrtle *Lagerstroemia indica***: **Potyviridae** *Potyvirus* Watermelon mosaic virus

**34. Malvaceae** order Malvales, clade Rosids, clade Eudicots, clade **Angiosperms**, kingdom Plantae

**Abutilon *Abutilon sp.***: **Pospiviroidae** *Hostuviroid*

**Chinese hibiscus *Hibiscus rosa-sinensis***: Unassigned (+)ssRNA Cilevirus Citrus leprosis virus cytoplasmic type 2

**Cotton *Gossypium sp.***: **Geminiviridae** *Begomovirus* Cotton leaf curl Multan virus, *Betasatellite* Cotton leaf curl Multan

betasatellite; **Luteoviridae** *Polerovirus* Cotton leafroll dwarf virus

**Velvet leaf *Abutilon theophrasti***: **Tospoviridae** *Orthotospovirus* Tomato spotted wilt virus

**35. Marsileaceae** order Salviniales, class Polypodiopsida/Pteridopsida, division **Pteridophyta**, kingdom Plantae

**European waterclover Four leaf clover *Marsilea quadrifolia***: **Luteoviridae** *Polerovirus* Turnip yellows virus

**36. Moraceae** order Rosales, clade Rosids, clade Eudicots, clade **Angiosperms**, kingdom Plantae

**Fig *Ficus carica***: **Betaflexiviridae** *Trichovirus* Fig latent virus 1; **Caulimoviridae** *Badnavirus* Fig badnavirus-1; **Closteroviridae** *Ampelovirus* Fig leaf mottle-associated virus 2, *Closterovirus* Fig mild mottle-associated virus; **Fimoviridae** *Emaravirus* Fig mosaic virus; **Tymoviridae** *Maculavirus;* **Pospiviroidae** *Apscaviroid* Apple dimple fruit viroid

**Mulberry *Morus sp.***: **Caulimoviridae** *Badnavirus* Mulberry badnavirus 1, **Tospoviridae** *Orthotospovirus*; *Unassigned* Mulberry mosaic dwarf-associated virus

**37. Musaceae** order Zingiberales, clade Commelinids, clade Monocots, clade **Angiosperms**, kingdom Plantae

**Banana *Musa acuminata***: **Betaflexiviridae** *Unassigned* Banana mild mosaic virus; **Caulimoviridae** *Badnavirus* Banana streak Obino l'Ewai virus Banana streak Goldfinger virus Banana streak Imove virus Banana streak Mysore virus Banana streak Vietnam virus Banana streak Cavendish virus, *Endogenous Badna*-like;

**38. Myrtaceae** order Myrtales, clade Rosids, clade Eudicots, clade **Angiosperms**, kingdom Plantae

***Eucalyptus grandis***: **Caulimoviridae** *Florendovirus* endogenous Eucalyptus grandis florendovirus 1 Eucalyptus grandis florendovirus 4, *Endogenous Petuvirus*-lke Eucalyptus grandis endogenous viral element 1

**39. Nymphaeaceae** order Nymphaeales clade **Angiosperms**, kingdom Plantae

**Water lily**: **Bromoviridae** *Cucumovirus* Cucumber mosaic virus; **Caulimoviridae** Endogenous; **Rhabdoviridae** *Cytorhabdovirus*

**40. Oleaceae** order [Lamiales](https://en.wikipedia.org/wiki/Lamiales) clade Asterids, clade Eudicots, clade **Angiosperms**, kingdom Plantae

**European privet *Ligustrum vulgare***: **Virgaviridae** *Hordeivirus* Ligustrum mosaic virus

**Japanese privet *Ligustrum japonicum***: **Unassigned (+)ssRNA** *Idaeovirus* Privet leaf blotch-associated virus

**Japanese tree lilac *Syringa reticulata***: **Alphaflexiviridae** *Potexvirus;* **Betaflexiviridae** *Carlavirus* Ligustrum virus A

**Jasmine *Jasminum sp.***: **Tombusviridae** *Pelarspovirus* Jasmine virus H

**Lilac *Syringa vulgaris***: **Caulimoviridae** *Caulimovirus,* **Geminiviridae** *Begomovirus*

**41. Paeoniaceae** order Saxifragales, clade Eudicots, clade **Angiosperms**, kingdom Plantae

**Peony *Paeonia sp.***: **Secoviridae** *Nepovirus* Cycas necrotic stunt virus; **Virgaviridae** *Tobravirus* Tobacco rattle virus; **Unassigned** Mulberry **viroid**-like RNA

**42.** **Pedaliaceae** order [Lamiales](https://en.wikipedia.org/wiki/Lamiales) clade Asterids, clade Eudicots, clade **Angiosperms**, kingdom Plantae

**Sesame *Sesamum indicum***: **Potyviridae** *Potyvirus* Bean common mosaic virus Tobacco vein banding mosaic virus Watermelon mosaic virus Zucchini yellow mosaic virus

**43. Plantaginaceae** order [Lamiales](https://en.wikipedia.org/wiki/Lamiales) clade Asterids, clade Eudicots, clade **Angiosperms**, kingdom Plantae

***Plantago lanceolata***: **Geminiviridae** *Capulavirus* Plantago lanceolata latent virus

**Snapdragon *Antirrhinum sp.***: **Secoviridae** *Nepovirus* Cycas necrotic stunt virus

**44. Poaceae** order Poales, clade Commelinids, clade Monocots, clade **Angiosperms**, kingdom Plantae

**Bamboo**: Bamboo mosaic virus, Bamboo mosaic virus **satellite** RNA

**Barley** ***Hordeum vulgare***: **Endornaviridae** *Alphaendornavirus* Hordeum vulgare endornavirus; **Luteoviridae** *Polerovirus* Barley yellow dwarf virus; **Virgaviridae** *Hordeivirus* Barley stripe mosaic virus

**Cocksfoot, orchard grass *Dactylis glomerata***: **Luteoviridae** *Polerovirus* Cereal yellow dwarf virus; **Potyviridae** *Potyvirus* Cocksfoot streak virus, *Tritimovirus* Cocksfoot steak mosaic virus

**Common reed** ***Phragmites* sp**.: **Potyviridae** *Unassigned* Common reed chlorotic stripe virus

**Fountain grass 'Rubrum' *Pennisetum advena***: **Secoviridae** *Waikavirus* Maize chlorotic dwarf virus

**Maize *Zea mays***: **Geminiviridae** *Mastrevirus* Maize streak Reunion virus; **Luteoviridae** *Polerovirus* Maize yellow dwarf virus Maize yellow mosaic virus; **Potyviridae** *Potyvirus* Sugarcane mosaic virus **Reovirirdae** *Fijivirus* Rice black streaked dwarf virus; **Secoviridae** *Waikavirus* Maize chlorotic dwarf virus; **Tombusviridae** *Machlomovirus* Maize chlorotic mottle virus

***Miscanthus sinenesis***: **Potyviridae** *Potyvirus* Sorghum mosaic virus

**Oat *Avena sativa***: **Closteroviridae** *Closterovirus* Cereal closterovirus; **Luteoviridae** *Polerovirus* Cereal yellow dwarf virus Barley yellow dwarf virus; **Phenuiviridae** *Tenuivirus* European wheat striate mosaic virus; **Reovirirdae** *Fijivirus* Oat sterile dwarf virus

**Rice *Oryza sativa***: **Caulimoviridae** *Tungrovirus* Rice tungro bacilliform virus; **Phenuiviridae** *Tenuivirus* Rice hoja blanca virus Rice stripe virus; **Rhabdoviridae** *Cytorhabdovirus* unclassified Rice stripe mosaic virus; **Reovirirdae** *Fijivirus* Southern rice black-streaked dwarf virus, *Oryzavirus* Rice ragged stunt virus, *Phytoreovirus* Rice dwarf virus; **Secoviridae** *Waikavirus* Rice tungro spherical virus

**Rye *Secale cereale***: **Luteoviridae** *Polerovirus* Barley yellow dwarf virus

**Sorghum *Sorghum bicolor***: **Caulimoviridae** *Florendovirus* endogenous Sorghum bicolor virus

**Sugarcane *Saccharum* sp**.: **Geminiviridae** *Mastrevirus* Sugarcane streak Egypt Virus Sugarcane white streak virus

**Wheat *Triticum avestum***: **Bromoviridae** *Bromovirus* Brome mosaic virus; **Closteroviridae** *Closterovirus* Cereal closterovirus; **Geminiviridae** *Mastrevirus* Wheat dwarf virus; **Luteoviridae** *Polerovirus* Barley yellow dwarf virus Wheat leaf yellowing-associated virus; **Phenuiviridae** *Tenuivirus* European wheat striate mosaic virus; **Potyviridae** *Bymovirus* Wheat yellow mosaic virus, *Poacevirus* Triticum mosaic virus*, Tritimovirus* Wheat streak mosaic virus; **Rhabdoviridae** *Cytorhabdovirus* Barley yellow striate mosaic virus; **Reovirirdae** *Fijivirus* Mal de Río Cuarto virus Oat sterile dwarf virus; **Solemoviridae** *Sobemovirus* Cynosurus mottle virus; **Virgaviridae** *Furovirus* Chinese wheat mosaic virus

**45. Ranunculaceae** order Ranunculales, clade Eudicots, clade **Angiosperms**, kingdom Plantae

**Anemone**: **Amalgaviridae** *Amalgavirus*; **Endornaviridae** *Alphaendornavirus*; **Partitiviridae** *Deltapartitivirus;* **Potyviridae** *Potyvirus;* **Tospoviridae** *Orthotospovirus* Impatiens necrotic spot virus

**Buttercup *Ranunculus* sp.**: **Potyviridae** *Potyvirus* Ranunculus leaf distortion virus Ranunculus mild mosaic virus; **Tospoviridae** *Orthotospovirus* Tomato spotted wilt virus

**46. Rosaceae** order Rosales, clade Rosids, clade Eudicots, clade **Angiosperms**, kingdom Plantae

**Apple *Malus x domestica***: **Betaflexiviridae** *Capillovirus* Apple stem grooving virus, *Foveavirus* Apple stem pitting virus, *Trichovirus* Apple chlorotic leaf spot virus; **Geminiviridae** *Unassigned* Apple geminivirus; **Secoviridae** *Cheravirus* Apple latent spherical virus; **Avsunviroidae** *Pelamoviroid* tentative Apple hammerhead viroid; **Pospiviroidae** *Apscaviroid* Apple scar skin viroid

**Apricot *Prunus* sp.**: **Betaflexiviridae** *Capillovirus* Cherry virus A; **Closteroviridae** *Velarivirus* Little cherry virus 1; **Potyviridae** *Potyvirus* Plum pox virus

**Crab apple *Malus sp***: **Betaflexiviridae** *Capillovirus* Apple stem grooving virus, *Trichovirus* Apple chlorotic leaf spot virus; **Bromoviridae** *Ilarvirus* Prunus necrotic ringspot virus

**Japanese plum *Prunus*** ***salicina***: **Potyviridae** *Potyvirus* Plum pox virus

**Peach *Prunus persica***: **Betaflexiviridae** *Trichovirus* Apple chlorotic leaf spot virus, *Unassigned* Cherry green ring mottle virus; **Bromoviridae** *Ilarvirus* Prunus necrotic ringspot virus; **Closteroviridae** *Ampelovirus* Plum bark necrosis stem pitting-associated virus; **Luteoviridae** *Unassigned* Nectarine stem pitting-associated virus; **Secoviridae** *Fabavirus* Peach leaf pitting-associated virus; **Avsunviroidae** *Pelamoviroid* Peach latent mosaic viroid, **Pospiviroidae** *Hostuviroid* Hop stunt viroid

**Pear *Pyrus* sp.**: **Betaflexiviridae** *Capillovirus* Apple stem grooving virus

***Prunus sp.***: **Avsunviroidae** *Pelamoviroid* Peach latent mosaic viroid

**Quince *Cydonia oblonga***: **Betaflexiviridae** *Foveavirus* Apple green crinkle associated virus

**Raspberry *Rubus* sp**.: **Caulimoviridae** *Badnavirus* Rubus yellow net virus; **Fimoviridae** *Emaravirus* Raspberry leaf blotch virus; **Secoviridae** *Unassigned* Black raspberry necrosis virus; **Unassigned (+)ssRNA** *Idaeovirus* Raspberry bushy dwarf virus

**Rose *Rosa* sp.**: **Betaflexiviridae** *Capillovirus* Apple stem grooving virus; **Bromoviridae** *Ilarvirus* Blackberry chlorotic ringspot virus Prunus necrotic ringspot virus; **Closteroviridae** *Closterovirus* Rose leaf rosette-associated virus

**Strawberry *Fragaria × ananassa***: **Alphaflexiviridae** *Potexvirus* Strawberry mild yellow edge virus; **Caulimoviridae** *Caulimovirus* Strawberry vein banding virus; **Closteroviridae** *Crinivirus* Strawberry crinivirus 4 Strawberry pallidosis associated virus; **Luteoviridae** *Polerovirus* Strawberry polerovirus 1; **Secoviridae** *Unassigned* Strawberry mottle virus

**Sweet cherry *Prunus avium***: **Betaflexiviridae** *Capillovirus* Cherry virus A, *Citrivirus* Citrus leaf blotch virus; **Bromoviridae** *Ilarvirus* Apple mosaic virus Prune dwarf virus Prunus necrotic ringspot virus; **Closteroviridae** *Ampelovirus* Little cherry virus 2 Plum bark necrosis stem pitting-associated virus, *Velarivirus* Little cherry virus 1; **Secoviridae** *Fabavirus* Cherry virus F; **Pospiviroidae** *Hostuviroid* Hop stunt viroid

**47. Rutaceae** order Sapindales, clade Rosids, clade Eudicots, clade **Angiosperms**, kingdom Plantae

**Alemow *Citrus macrophylla***: **Closteroviridae** *Closterovirus* Citrus tristeza virus

**Blood orange *Citrus × sinensis***: **Unassigned** **(-)ssRNA** *Phlebovirus*-like (*Ophiovirus*-like RNA2 for MP); **Pospiviroidae** *Apscaviroid* Citrus dwarfing viroid, *Hostuviroid* Hop stunt viroid, *Pospiviroid* Citrus exocortis viroid

**Citron *Citrus medica***: **Closteroviridae** *Closterovirus* Citrus tristeza virus; **Luteoviridae** *Enamovirus* Citrus vein enation virus; **Pospiviroidae** *Apscaviroid* Citrus viroid VI, *Hostuviroid* Hop stunt viroid

***Citrus* sp.**: **Caulimoviridae** Endogenous *Petuvirus*-like Citrus endogenous pararetrovirus; **Rhabdoviridae** *Dichorhavirus* Orchid fleck virus (Citrus leprosis virus nuclear type)

**Clementine *Citrus × clementina***: **Caulimoviridae** *Florendovirus* endogenous Citrus clementina virus

**Grapefruit *Citrus × paradisi***: **Closteroviridae** *Closterovirus* Citrus tristeza virus; **Pospiviroidae** *Apscaviroid* Citrus dwarfing viroid

**Lemon *Citrus limon***: **Alphaflexiviridae** *Mandarivirus* Citrus yellow vein clearing virus; **Betaflexiviridae** *Citrivirus* Citrus leaf blotch virus; **Closteroviridae** *Closterovirus* Citrus tristeza virus; **Geminiviridae** *Unassigned* Citrus chlorotic dwarf-associated virus; **Pospiviroidae** *Apscaviroid* Citrus viroid III Citrus viroid VI, *Hostuviroid* Hop stunt viroid, *Cocadviroid* Citrus bark cracking viroid, *Pospiviroid* Citrus exocortis viroid

**Mandarin orange *Citrus reticulata***: **Closteroviridae** *Closterovirus* Citrus tristeza virus

**Mexican Lime *Citrus × aurantiifolia***: **Closteroviridae** *Closterovirus* Citrus tristeza virus

**Persian Lime *Citrus × latifolia***: **Closteroviridae** *Closterovirus* Citrus tristeza virus

**Pomelo *Citrus sp.***: **Caulimoviridae** Endogenous

**Sour orange *Citrus × aurantium***: **Closteroviridae** *Closterovirus* Citrus tristeza virus

**Sweet orange *Citrus × sinensis***: **Caulimoviridae** *Florendovirus* endogenous Citrus clementina virus, Endogenous *Petuvirus*-like Citrus endogenous pararetrovirus; **Closteroviridae** *Closterovirus* Citrus tristeza virus; **Rhabdoviridae** *Dichorhavirus* Citrus leprosis virus nuclear type (Orchid fleck virus); **Tymoviridae** *Marafivirus* Citrus sudden death-associated virus; **Unassigned** **(+)ssRNA** *Cilevirus* Citrus leprosis virus cytoplasmic type 2; **Pospiviroidae** *Apscaviroid* Citrus dwarfing viroid, *Pospiviroid* Citrus exocortis viroid

**Trifoliate orange *Citrus trifoliata***: **Closteroviridae** *Closterovirus* Citrus tristeza virus

**48. Salicaceae** order Malpighiales, clade Rosids, clade Eudicots, clade **Angiosperms**, kingdom Plantae

**Poplar *Populus trichocarpa***: **Caulimoviridae** *Florendovirus* endogenous Populus trichocarpa virus, Endogenous *Caulimo-Sobemo*-like

**49. Solanaceae** order Solanales, clade Asterids, clade Eudicots, clade **Angiosperms**, kingdom Plantae

**Bell pepper *Capsicum annuum***: **Endornaviridae** *Alphaendornavirus* Bell pepper endornavirus; **Luteoviridae** *Polerovirus* Pepper leafroll chlorosis virus; **Potyviridae** *Potyvirus* Pepper veinal mottle virus; **Tospoviridae** *Orthotospovirus* Tomato spotted wilt virus **Virgaviridae** *Tobamovirus* Youcai mosaic virus

**Eggplant *Solanum melongena***: **Virgaviridae** *Tobamovirus* Tomato mottle mosaic virus

***Nicotiana clevelandii***: **Solemoviridae** *Sobemovirus* Solanum nodiflorum mottle virus Velvet tobacco mottle virus

***Nicotiana benthamiana***: **Alphaflexiviridae** *Potexvirus* Bamboo mosaic virus Potato virus X, Bamboo mosaic virus **satellite** RNA; **Bromoviridae** *Cucumovirus* Cucumber mosaic virus Cucumber mosaic virus **satellite** Y-Sat; **Closteroviridae** *Crinivirus* Lettuce infectious yellows virus; **Geminiviridae** *Begomovirus* Tomato yellow leaf curl China virus, *Betasatellite* Tomato yellow leaf curl China betasatellite; **Luteoviridae** *Polerovirus* Brassica yellows virus; **Phenuiviridae** *Tenuivirus* Rice stripe virus; **Potyviridae** *Potyvirus* Plum pox virus; **Secoviridae** *Torradovirus*-like; **Tombusviridae** *Alphanecrovirus* Corn salad necrosis virus, *Betacarmovirus* Turnip crinkle virus Beet black scorch virus, Beet black scorch virus **satellite** RNA, *Pelarspovirus* Pelargonium line pattern virus, *Tombusvirus* Cymbidium ringspot virus, *Umbravirus* Pea enation mosaic virus 2; **Tospoviridae** *Orthotospovirus* Chrysanthemum stem necrosis virus Polygonum ringspot virus Tomato spotted wilt virus; **Virgaviridae** *Furovirus* Chinese wheat mosaic virus, *Tobamovirus* Pepper mild mottle virus Youcai mosaic virus; **Pospiviroidae** Pospiviroid Potato spindle tuber viroid

**Petunia *Petunia x hybrida***: **Caulimoviridae** *Petuvirus* Petunia vein clearing virus; **Potyviridae** Potyvirus

**Pepper *Piper nigrum***: **Luteoviridae** *Polerovirus* Pepper vein yellows virus; **Virgaviridae** *Tobamovirus* Paprika mild mottle virus

**Physalis *Physalis ﬂoridiana*: Caulimoviridae** *Solendovirus* endogenous *Tobacco vein-clearing virus-*like

**Potato *Solanum tuberosum***: **Alphaflexiviridae** *Potexvirus* Potato aucuba mosaic virus Potato virus X, **Betaflexiviridae** *Carlavirus* Potato virus H Potato virus S, *Tepovirus* Potato virus T; **Bromoviridae** *Alfamovirus* Alfalfa mosaic virus; **Caulimoviridae** *Florendovirus* endogenous Solanum tuberosum virus, *Solendovirus* endogenous Tobacco vein-clearing virus-like, **Luteoviridae** *Polerovirus* Potato leafroll virus, **Potyviridae** *Potyvirus* Potato virus A Potato virus Y Potato yellow blotch virus; **Secoviridae** *Nepovirus* Potato virus B; **Tombusviridae** *Alphanecrovirus* Tobacco necrosis virus A; **Tymoviridae** *Tymovirus* Andean potato latent virus Andean potato mild mosaic virus

**Red pepper *Capsicum frutescens***: **Luteoviridae** *Polerovirus* Pepper vein yellows virus

**Tobacco *Nicotiana tabacum***: **Bromoviridae** *Alfamovirus* Alfalfa mosaic virus, *Cucumovirus* Cucumber mosaic virus, Cucumber mosaic virus *satellite* Y-Sat, *Endogenous* Cucumber mosaic virus satellite; **Caulimoviridae** *Solendovirus* Tobacco vein-clearing virus; **Luteoviridae** *Polerovirus* Brassica yellows virus Turnip yellows virus; **Potyviridae** *Potyvirus* Pepper mottle virus Potato virus A Potato virus Y Tobacco vein banding mosaic virus; **Secoviridae** *Fabavirus* Broad bean wilt virus 2; **Virgaviridae** *Tobamovirus* Tobacco mosaic virus

**Tomato *Solanum lycopersicum***: **Alphaflexiviridae** *Potexvirus* Pepino mosaic virus; **Amalgaviridae** *Amalgavirus* Southern tomato virus; **Betaflexiviridae** *Carlavirus* Potato virus H Potato virus S; **Bromoviridae** *Cucumovirus* Cucumber mosaic virus Tomato aspermy virus, *Ilarvirus* Spinach latent virus; **Caulimoviridae** *Florendovirus* endogenous Solanum lycopersicum virus*, Solendovirus* endogenous Tobacco vein-clearing virus-like; **Closteroviridae** *Crinivirus* Lettuce chlorosis virus Tomato chlorosis virus; **Geminiviridae** *Begomovirus* Tomato latent virus Tomato yellow leaf curl China virus Tomato yellow leaf curl Sardinia virus Tomato yellow leaf curl virus, *Betasatellite* Tomato yellow leaf curl China betasatellite; **Luteoviridae** *Polerovirus* Turnip yellows virus; **Potyviridae** *Potyvirus* Chilli veinal mottle virus Henbane mosaic virus Pepper mottle virus Pepper veinal mottle virus Potato virus A Potato virus Y Tobacco vein banding mosaic virus Tomato necrotic stunt virus; **Rhabdoviridae** *Cytorhabdovirus* Tomato yellow mottle-associated virus; **Tospoviridae** *Orthotospovirus* Capsicum chlorosis virus Polygonum ringspot virus Tomato spotted wilt virus Tomato zonate spot virus; **Virgaviridae** *Tobamovirus* Tobacco mosaic virus Tomato brown rugose fruit virus Tomato mosaic virus Tomato mottle mosaic virus; **Pospiviroidae** *Pospiviroid* Citrus exocortis viroid Columnea latent viroid Potato spindle tuber viroid Tomato apical stunt viroid; **Unassigned** Iflaviridae-like Tomato matilda virus

**50. Theaceae** order Ericales, clade Asterids, clade Eudicots, clade **Angiosperms**, kingdom Plantae

**Camellia *Camellia sp.***: **Geminiviridae** Unassigned Camellia chlorotic dwarf-associated virus

**51. Verbenaceae** order Lamiales, clade Asterids, clade Eudicots, clade **Angiosperms**, kingdom Plantae

**Verbena *Verbena sp.***: **Tombusviridae** *Alphacarmovirus* Angelonia flower break virus

**52. Vitaceae** order Vitales, clade Rosids, clade Eudicots, clade **Angiosperms**, kingdom Plantae

**Grapevine *Vitis vinifera, Vitis sp.***: **Betaflexiviridae** *Foveavirus* Apple green crinkle associated virus, *Trichovirus* Grapevine berry inner necrosis virus Grapevine Pinot gris virus, *Vitivirus* Grapevine virus A Grapevine virus B Grapevine virus G Grapevine virus I; **Caulimoviridae** *Badnavirus* Grapevine Roditis leaf discoloration-associated virus Grapevine vein clearing virus, *Florendovirus* endogenous Vitis vinifera A virus Vitis vinifera B virus; **Closteroviridae** *Ampelovirus* Grapevine leafroll-associated virus 1 Grapevine leafroll-associated virus 3 Grapevine leafroll-associated virus 4, *Closterovirus* Grapevine leafroll-associated virus 2, *Velarivirus* Grapevine leafroll-associated virus 7; **Geminiviridae** *Grablovirus* Grapevine red blotch virus, *Unassigned* Grapevine geminivirus A; **Secoviridae** *Fabavirus* Grapevine fabavirus *Nepovirus* Grapevine chrome mosaic virus Grapevine fanleaf virus, Grapevine fanleaf virus **satellite** RNA; **Tymoviridae** *Maculavirus* Grapevine fleck virus Grapevine red globe virus, *Marafivirus* Grapevine asteroid mosaic associated virus Grapevine rupestris vein feathering virus Grapevine Syrah virus 1; **Unassigned (+)ssRNA** *Idaeovirus* Raspberry bushy dwarf virus; **Pospiviroidae** *Apscaviroid* Australian grapevine viroid Grapevine latent viroid Grapevine yellow speckle viroid 1 Grapevine yellow speckle viroid 2, *Hostuviroid* Hop stunt viroid; **Unassigned viroid** Grapevine hammerhead viroid-like RNA; **Unassigned (+)ssRNA Satellite virus** Grapevine satellite virus (of unknown helper virus)

**53. Zingiberaceae** order Zingiberales, clade Commelinids, clade Monocots, clade **Angiosperms**, kingdom Plantae

**Alpinia *Alpinia oxyphylla***: **Potyviridae** *Macluravirus* Alpinia oxyphylla mosaic virus

**Canna *Canna* sp.**: **Potyviridae** *Potyvirus* Bean yellow mosaic virus Canna yellow streak virus*;* **Caulimoviridae** *Badnavirus*

**Ginger *Canna* sp.**: **Tombusviridae** *Panicovirus* Ginger chlorotic fleck virus
